# Fully Transparent Haptic Interface for High‐Resolution Tactile Feedback on Touchscreens

**DOI:** 10.1002/advs.202511874

**Published:** 2025-08-19

**Authors:** Boxue Shan, Yuan Guo, Yun Wang, Pengbo Zhao, Yiheng Wang, Zemin Wang, Liwen He, Yan Liu, Yibin Wang, Weidong Guo, Yuru Zhang, Zhaohe Dai, Xinge Yu, Dangxiao Wang

**Affiliations:** ^1^ State Key Laboratory of Virtual Reality Technology and Systems Beihang University Beijing 100191 China; ^2^ Robotics Institute School of Mechanical Engineering and Automation Beihang University Beijing 100191 China; ^3^ Department of Biomedical Engineering City University of Hong Kong Hong Kong SAR 999077 China; ^4^ Department of Mechanics and Engineering Science College of Engineering Peking University Beijing 100871 China

**Keywords:** 3D architecture, fully transparent, high‐resolution, morphable haptic interface, touchscreen interaction

## Abstract

Haptic technology has the potential to bring tactile richness to touchscreens on smartphones, tablets, and laptops, unlocking new dimensions for digital interaction and communication. Yet, despite notable advancements in visual resolution, the resolution of tactile pixels—referred to as “taxels”—lags significantly behind, limiting the immersive tactile feedback required for a truly enriched user experience. To bridge this gap, the study presents a transparent haptic interface with a 3D architecture that dynamically reconfigures high‐resolution taxels through a densely integrated actuator array. Each actuator can be precisely inflated through fluid pressure to deliver tactile feedback with exceptional clarity and density, surpassing both the tactile perception and two‐point discrimination thresholds of human fingertips. This haptic interface reveals transformative potential for enhancing touchscreen interactions in applications such as touch panel control, virtual exploration, and gaming, as it can be reversibly attached to various touchscreens and create nuanced topographical features that align with on‐screen visuals.

## Graphical abstract

Integrating haptic feedback into touchscreens—enabling users to feel what they see on screen—has been a longstanding goal. In this article, B. Shan et al develop a transparent morphable haptic interface that transforms touchscreens into finely detailed, programmable morphable surfaces, enabling more effective and immersive human‐computer interaction.

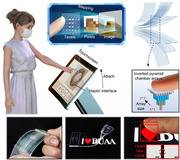

## 1 Introduction

Touchscreens are indispensable in today's electronic devices, enabling digital information display and user interaction across various applications, from social media and navigation systems to self‐service terminals and digital games.^[^ ^1^, ^2^, ^3^, ^4^, ^5^ ^]^ Integrating haptic feedback into touchscreens—enabling users to feel what they see on screen—has been a longstanding goal.^[^ ^6^, ^7^, ^8^ ^]^ Over the past decade, significant efforts have focused on developing haptic technology that can complement visual and audio stimuli.^[^ ^9^, ^10^, ^11^, ^12^ ^]^ So far, several approaches have been demonstrated, including mechanical vibration,^[^ ^13^, ^14^ ^]^ electrostatic effect,^[^ ^15^, ^16^, ^17^ ^]^ ultrasonic,^[^ ^18^, ^19^, ^20^ ^]^ electrical stimulation,^[^ ^21^, ^22^ ^]^ and shape‐morphing technology (Figure S1, Supporting Information).^[^ ^23^, ^24^, ^25^, ^26^ ^]^

Among these approaches, morphable haptic devices, which physically reshape the touchscreen surface through an array of actuators, stand out due to their ability to generate perceivable topographies synchronized with graphical content. Existing implementations utilize hydraulic,^[^ ^25^ ^]^ electromagnetic,^[^ ^26^ ^]^ electroosmotic,^[^ ^27^ ^]^ pneumatic,^[^ ^28^, ^29^ ^]^ hydrogel,^[^ ^30^ ^]^ and electrohydraulic actuation^[^ ^31^ ^]^ to create localized surface protrusions, allowing users to perceive tactile contours and raised elements aligned with on‐screen virtual buttons. This physical feedback enhances interaction speed and accuracy, especially in contexts involving distraction or visual occlusion.^[^ ^5^, ^32^ ^]^

However, practical applications of morphable haptic devices for touchscreens remain in the early stages due to fundamental limitations in spatial resolution and real‐time programmability.^[^ ^25^, ^26^, ^27^, ^28^, ^29^, ^30^, ^31^ ^]^ As a result, current implementations are mainly limited to binary on/off buttons, preconfigured tactile patterns, and spatially coarse force feedback. Moreover, transparency is also a critical factor, as integrated actuator structures in devices introduce light scattering and refraction artifacts,^[^ ^29^, ^30^, ^31^ ^]^ hindering seamless integration with high‐resolution touchscreen displays. In contrast, state‐of‐the‐art touchscreens excel in visual and interactive capabilities, offering high‐resolution graphics display, dynamic visual content, and personalized user interface (UI).^[^ ^4^, ^7^ ^]^ Therefore, the development of a transparent, high‐resolution, and dynamically programmable haptic feedback system is key to advancing touchscreen technology toward more precise interaction operation and greater immersion experience (Table S1, Supporting Information).^[^ ^6^, ^7^, ^21^ ^]^

**Figure 1:**
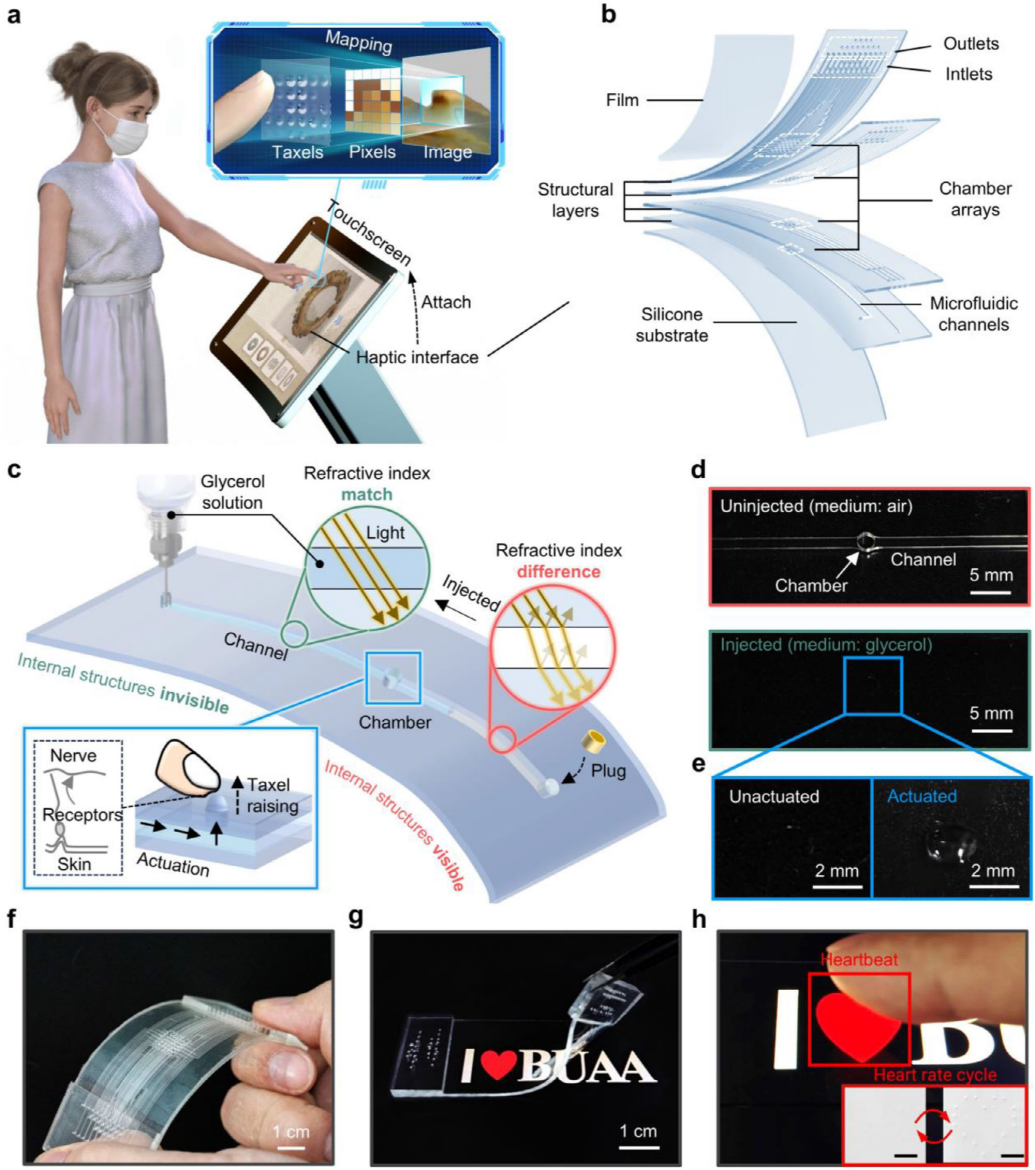
Design and architecture of the optically transparent and high spatial resolution haptic interface. a) Schematic diagram of pixel‐to‐taxel mapping based on the transparent haptic interface. b) Exploded view schematic illustration of the microfluidic‐integrated haptic device with 49 independently controlled transparent actuators, comprising chamber arrays of varying sizes, interconnected input and output channels, and a surface‐covered film. The center distance between adjacent chambers is 1.5 mm. c) Design principles of transparent microfluidic actuators, including the invisibility mechanism of internal structures and the hydraulic driving principle. d) Optical image of the actuator before and after injection. Scale bar, 5 mm. e) Optical images of the actuator in both un‐actuated and actuated states. Scale bar, 2 mm. f) Optical image of an unfilled haptic interface while being bent. g) Reversible attachment of the transparent tactile interface to the touchscreen, and h) local details of the haptic device in the actuating state. Scale bar, 3 mm. The raised taxels formulated a heart‐shaped outline that perfectly matched the image of the touchscreen. Note that, to facilitate the visualization of the transparent actuators, a thin gray coating is sprayed on the surface of the haptic device.

Here, we present an optically transparent, high‐resolution, and dynamically programmable microfluidic haptic interface that seamlessly integrates with touchscreens, simultaneously offering fine‐grained tactile feedback and maintaining superior visual clarity through its transparent architecture (**Figure** **1**a). Our novel inverted pyramid microfluidic structure design enables the dense integration of microfluidic chambers with a pitch of 1.5 mm and a density of 49 dots/cm^2^—surpassing the two‐point discrimination threshold of human fingertips (Figure S2, Supporting Information).^[^ ^33^ ^]^ By modulating the activation positions, timing, and pressure of the chamber array, our system generates precise, dynamic topographic changes synchronized with on‐screen content, delivering rich and localized mechanical stimuli. In addition, by precisely tuning the refractive index of a glycerol‐based solution to match that of the PDMS material, we achieve optical transparency and eliminate the visibility of internal microfluidic structures. This index‐matching strategy effectively minimizes light scattering and refraction within the transparent haptic interface, thereby preserving the optical clarity of the touchscreen. We envision that this transparent, millimeter‐resolution, thin, programmable, and adaptable haptic interface offers the potential to significantly enrich user interaction and elevate immersion in advanced touchscreen applications. This includes generating high‐resolution tactile patterns consistent with UI graphics and textures, as well as delivering enriched haptic feedback for sliding interactions and gaming joysticks.

### 1.1 Design and Architecture of the Haptic Interface

To enable high‐fidelity haptic feedback for touchscreens, we aim to develop an optically transparent, high‐resolution, and programmable haptic interface. This interface integrates a high‐density microfluidic actuator array capable of dynamically generating microscopic topographies in response to visual content on the touchscreen (Figure 1a). The core innovation lies in directly mapping visual pixels to tactile taxels, allowing users to both perceive displayed graphics and simultaneously experience corresponding finely detailed tactile feedback upon touch. This functionality requires that, as the visual content changes, the actuator array dynamically adjusts its activation positions, timing, and intensity, enabling the reconstruction of static tactile patterns as well as the real‐time playback of dynamic tactile animations. By bridging the gap between visual display and touch‐based interaction, this interface would empower users to interact with electronic devices more effectively and intuitively, significantly enhancing the interactive experience.

The haptic interface features a multilayered polydimethylsiloxane‐based structure (Figure 1b), comprising: i) a flexible film layer that deforms under actuation, ii) structured microfluidic layers housing the actuator array, and iii) a compliant substrate layer ensuring mechanical stability. Each layer is fabricated using soft lithography and replica molding, optimized for mechanical modulus, layer thickness, and overall dimensions (Figures S3 and S4, Supporting Information). The microfluidic architecture features four chamber array scales and two distinct channel cross‐sectional sizes (Figure 1b; Figures S5 and S6, Supporting Information), designed to accommodate high‐density integration of actuators while ensuring reliable fluid pressure transmission and actuation stability. These custom PDMS layers are assembled via precision punching and plasma bonding, forming a 3D microfluidic network (Figure S6, Supporting Information). A high‐density 7 × 7 actuator array achieves a spatial resolution of 49 taxels/cm^2^, with a pitch of 1.5 mm and individual actuator unit size of 1 mm in diameter, exceeding the fingertip's two‐point discrimination threshold. This enables the device to render intricate tactile details within a fingertip‐sized region, a critical requirement for high‐fidelity haptic representation.^[^ ^34^ ^]^

One key challenge in integrating morphable haptic interfaces onto touchscreens is preserving visual clarity while embedding actuators.^[^ ^29^, ^30^, ^31^ ^]^ Light scattering and reflection at the fluid–solid interface can significantly degrade display visibility (Video S1, Supporting Information). To overcome this, we have developed liquid‐driven haptic actuators featuring a bidirectional channel, allowing for controlled fluid replacement (Figure 1c; Figure S7, Supporting Information). By introducing a glycerol‐based solution with a finely tuned refractive index matching that of PDMS, we minimize optical discontinuities at material interfaces. This effectively eliminates unwanted light scattering, rendering the actuator's internal structure optically seamless (Figure 1d). Consequently, the device itself and the embedded actuator structure visually merge, ensuring a clear and unobstructed touchscreen display. Once filled with the refractive index‐matched liquid and hermetically sealed, the actuators can be selectively pressurized to deform the flexible film layer, forming tactile taxels (Figure 1e). By precisely modulating the driving pressure over a tunable range of 0–80 kPa, taxels can produce both: i) static deformations for rendering stationary shapes and contours and ii) dynamic tactile vibrations (0–50 Hz) for delivering rich tactile sensations (Video S1, Supporting Information).

In the absence of fluid injection, the 7 × 7 microfluidic actuator array within the haptic interface is clearly visible (Figure 1f). This haptic interface, characterized by its flexibility, lightweight design (8.74 g), and compact profile (1.5 mm thickness), exhibits excellent adaptability for attachment to touchscreens (Figures S8 and S9, Supporting Information). Once a refractive‐index‐matched glycerol solution is injected, the internal structure becomes optically continuous, effectively eliminating any visual interference (Figure 1g; Figure S7 and Videos S2 and S3, Supporting Information). Leveraging its exceptional spatial resolution and optical transparency, the haptic interface enables the precise programming of tactile feedback, dynamically synchronized with visual elements in real time. For example, taxels can be driven simultaneously or sequentially along a heart‐shaped contour on the touchscreen surface (Figure 1h; Figure S10, Supporting Information).

### 1.2 Optical Performance of the Actuator

**Figure 2:**
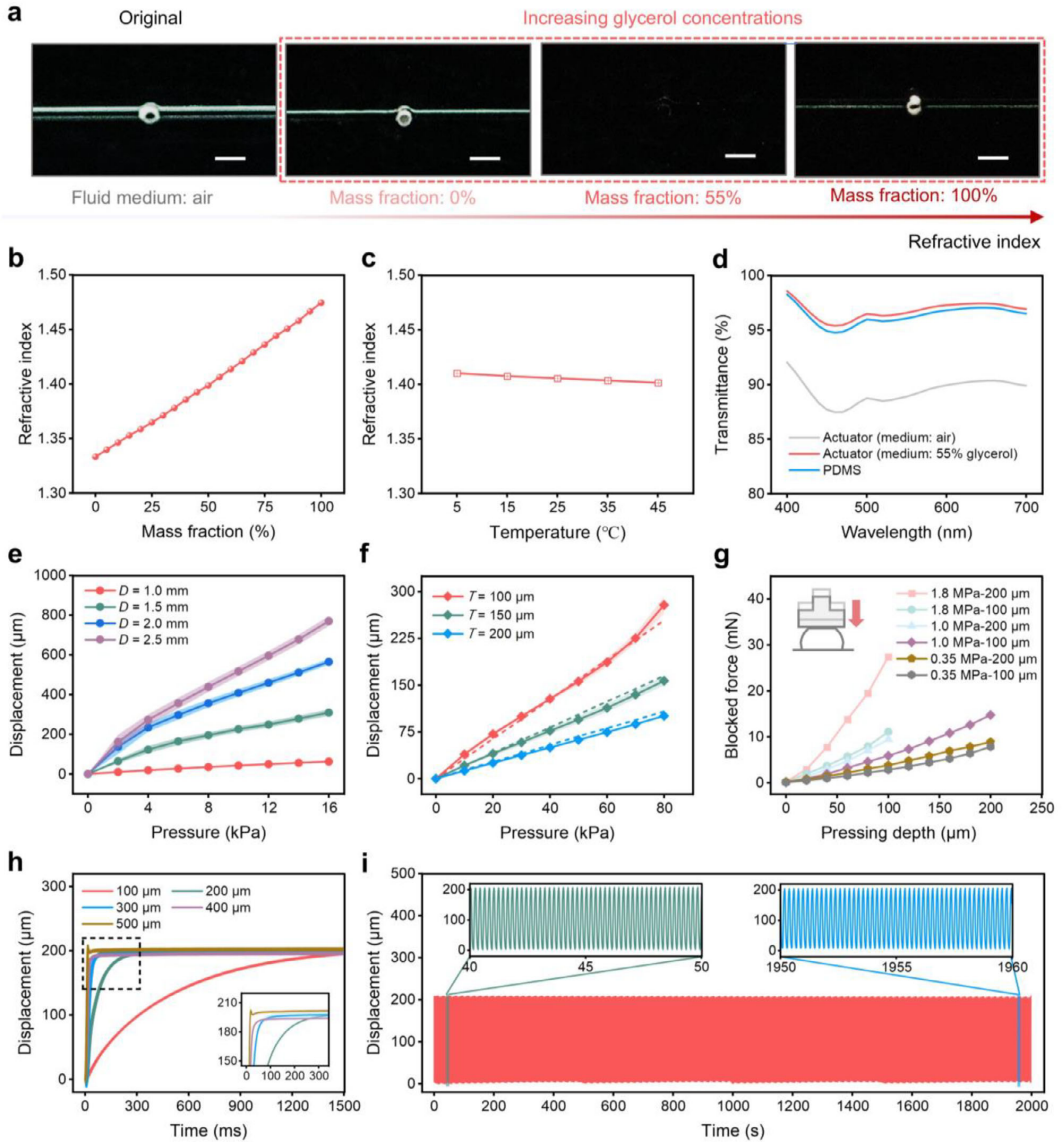
Characterization of the microfluidic actuator. a) Optical images of the actuator with different media. Scale bar, 2 mm. b) Refractive index of glycerol dissolved at the given mass fraction in water. Points represent mean values; error bars show standard deviation (s.d.); *n* = 3 independent samples. c) Refractive index of 55% mass fraction glycerol solutions at different temperatures. Points, mean; error bars, s.d.; *n* = 3 independent samples. d) Transmission spectra of the actuator filled with different media. Points, mean; error bars, s.d.; *n* = 3 independent samples. e) Average output displacement of the actuator with different chamber diameters under different driving pressures. This displacement corresponds to the maximum out‐of‐plane deformation during actuation. *D* represents the diameter of chambers. Points, mean; error bars, s.d.; *n*= 5 independent samples. f) Average output displacement of the actuator with different film thicknesses under different driving pressures. This displacement corresponds to the maximum out‐of‐plane deformation during actuation. *T* represents the thickness of films. Points, mean; error bars, s.d.; *n *= 5 independent samples. Solid lines denote experimental data and dotted lines denote simulation data. g) Blocked force changes versus pressing depth for the different films under the same driving pressure. Points, mean; error bars, s.d.; *n* = 3 independent samples. h) Cross‐sectional dimensions of the channel versus output displacement of the actuator over time. This displacement corresponds to the maximum out‐of‐plane deformation during actuation. The color represents the side length of the square cross‐section of the channel. Driving pressure: 60 kPa; acquisition frequency: 500 Hz. Points, mean; error bars, s.d.; *n* = 8 independent samples. i) Long‐term stability test of the actuator. Driving pressure: 60 kPa; acquisition frequency: 1000 Hz.

Highly transparent haptic interfaces contribute to maintaining visual clarity while enabling tactile feedback, facilitating seamless integration with touchscreen displays. However, the internal microfluidic structures of actuators can introduce optical discontinuities due to refractive index mismatches between different materials. According to Snell's law, when light transitions between two media with different refractive indices (*n*_1_ and *n*_2_​), its trajectory bends, and a portion of the light is scattered or reflected.^[^ ^35^ ^]^ This effect becomes pronounced at interfaces between PDMS (*n* ≈ 1.41) and air (*n* = 1.0), making the actuator's internal structures highly visible (**Figure** **2**a). Beyond scattering, Fresnel reflection contributes to optical loss and reduced transparency.^[^ ^36^ ^]^

To address this issue, we leverage liquid‐based refractive index tuning to minimize optical artifacts and enhance transparency. Liquids typically have a higher refractive index than gases,^[^ ^37^ ^]^ allowing them to more closely approximate or even match that of the actuator's elastomeric material. A suitable liquid medium must satisfy multiple criteria: Its refractive index should closely match or be adjustable to approximate that of PDMS, while remaining colorless, chemically stable, non‐toxic, and non‐conductive (Table S2, Supporting Information). We systematically evaluated various candidate fluids, including dimethicone oil, NaCl solution, sucrose solution, and glycerol solution. Several alternatives exhibited limitations such as swelling, precipitation, and a restricted modulation range, which compromised optical performance and long‐term stability (Figure S11, Supporting Information). Among these, glycerol‐based solutions proved to be the optimal choice, offering a precisely adjustable refractive index, excellent chemical stability, and biocompatibility.

The refractive index of glycerol solution is affected by its mass fraction and temperature. We measured twenty glycerol solutions at room temperature (25 °C), spanning mass fractions from 0% (pure water) to 100% (pure glycerol). The results show a near‐linear relationship between mass fraction and refractive index, with values ranging from 1.3332 to 1.4745 (Figure 2b). At 55% mass fraction, the refractive index closely matches PDMS, effectively rendering the actuator's internal structures optically invisible (Figure 2a). To evaluate temperature effects, we further characterized the refractive index of a 55 wt.% glycerol solution over a 5 to 45 °C range (Figure 2c). The results show minimal variation, with a maximum change of only 0.63%. Optical images of the actuator at various temperatures further confirmed that temperature variations have a negligible effect on actuator invisibility (Figure S12, Supporting Information).

Incorporating refractive index‐matched liquids significantly improves overall transmittance across the visible spectrum. As shown in Figure 2d, light transmittance was measured between 400–700 nm in both unfilled (air) and liquid‐injected (55 wt.% glycerol solution) states. The unfilled actuator (air medium) exhibits an average transmittance of ≈87%, with noticeable scattering and reflection. After injecting a 55 wt.% glycerol solution, transmittance exceeds 95%, closely matching that of pure PDMS, with a maximum deviation of only 0.63%. The enhanced transparency stems from the suppression of Fresnel reflections and interfacial scattering, preserving the clarity of touchscreen displays. Experimental results confirm that refractive index matching not only improves optical transparency but also maintains mechanical compliance. This seamless integration prevents visual artifacts and color distortion, ensuring that the actuator remains nearly invisible in its static state after liquid injection (Figure S7, Supporting Information).

### 1.3 Haptic Feedback Reproduced by the Microfluidic Actuator

The morphable haptic interface generates localized skin deformations through the morphological changes of actuators, stimulating different types of mechanoreceptors in the fingertips.^[^ ^23^, ^24^, ^25^, ^26^ ^]^ The deformation profile and temporal characteristics determine the primary mechanoreceptor activation: slow‐adapting (SA) receptors (e.g., Merkel cells and Ruffini endings) respond to sustained pressure and stretch, whereas fast‐adapting (FA) receptors (e.g., Meissner and Pacinian corpuscles) detect dynamic stimuli such as vibrations.^[^ ^38^ ^]^ To ensure perceptible haptic feedback, the microfluidic actuators must generate sufficient output displacement and blocked force. These parameters depend on key design factors, including chamber diameter, membrane thickness, and material elasticity, which were systematically analyzed through finite element analysis (FEA) and experimental validation (Figure S13, Supporting Information).

The output displacement of the actuator, which represents the maximum out‐of‐plane deformation during actuation, is primarily influenced by the input pressure, chamber diameter, membrane thickness, and elastic modulus of the material. This displacement is measured at the central point of the actuator's surface, as it best reflects the tactile response experienced by users during interaction. FEA simulations and experimental results confirm that increasing the chamber diameter enhances the actuator's output displacement (Figure 2e; Figure S14, Supporting Information). However, larger diameters reduce the packing density of the actuator array, limiting spatial resolution and information expressiveness of the haptic display. To balance these trade‐offs, we adopt a chamber with a diameter of 1 mm to retain the potential for a high‐density integrated actuator array. Furthermore, the mechanical properties of the membrane play a crucial role in determining actuator displacement (Figure S15, Supporting Information). As shown in Figure 2f, decreasing the membrane thickness increases the output displacement of the actuator (Figure S15a, Supporting Information), while lowering the elastic modulus reduces the required input pressure for achieving the same displacement (Figure S15b, Supporting Information). However, due to the damping effect of the skin, the actuator must also maintain sufficient stiffness to effectively transfer displacement into skin deformation, ensuring mechanoreceptor activation.^[^ ^39^, ^40^ ^]^

Apart from output displacement, blocked force is a crucial parameter that determines the actuator's effectiveness in delivering tactile stimuli.^[^ ^23^ ^]^ The blocked force depends on the stiffness of the membrane, which is governed by its thickness and elastic modulus.^[^ ^41^ ^]^ Experimental results reveal that, for the same output displacement, increasing the film thickness and elastic modulus enhances the blocked force, as higher stiffness requires greater pressure to achieve the desired deformation (Figure 2g). For actuators with a 100‐µm‐thick film and a modulus of 1.0 MPa, the blocked force progressively increases from 0 to 15.1 mN as the pressing depth rises from 0 to 200 µm (Figure 2g; Figure S16, Supporting Information). Notably, even at a moderate pressing depth, the actuator generates forces exceeding the absolute detection threshold of human tactile perception. To further validate its perceptual effectiveness, a psychophysical experiment was conducted with a group of volunteers (Figure S17, Supporting Information). The results showed that the minimum input pressure required to elicit detectable haptic feedback was 16.32 kPa. When the input pressure reached 50 kPa, all participants reported perceiving noticeable haptic feedback, corresponding to an actuator displacement of ≈156 µm and a blocked force of 11 mN.

The actuator's response time is influenced by the cross‐sectional dimensions of the microfluidic channels, as they determine the flow resistance and pressure propagation speed. As shown in Figure 2h, increasing the cross‐sectional dimensions reduces the time required to reach peak displacement. The color indicates the side length of the square channel cross‐section. A small hysteresis is observed in the loading and unloading process, and the microfluidic actuator can fully be restored to the initial state after unloading (Figure S18, Supporting Information). Figure S19 (Supporting Information) shows the output displacement of the microfluidic actuator at different actuation frequencies. As the actuation frequency increases, the output displacement decreases linearly, with the peak displacement reducing from 173.47 µm at 5 Hz to 5.82 µm at 50 Hz. Furthermore, the actuator reliably returns to its original state after unloading, exhibiting negligible plastic deformation even after 10000 actuation cycles (Figure 2i), demonstrating its excellent stability and robustness.

### 1.4 Spatial Layout and Characterizations for the Haptic Interface

**Figure 3:**
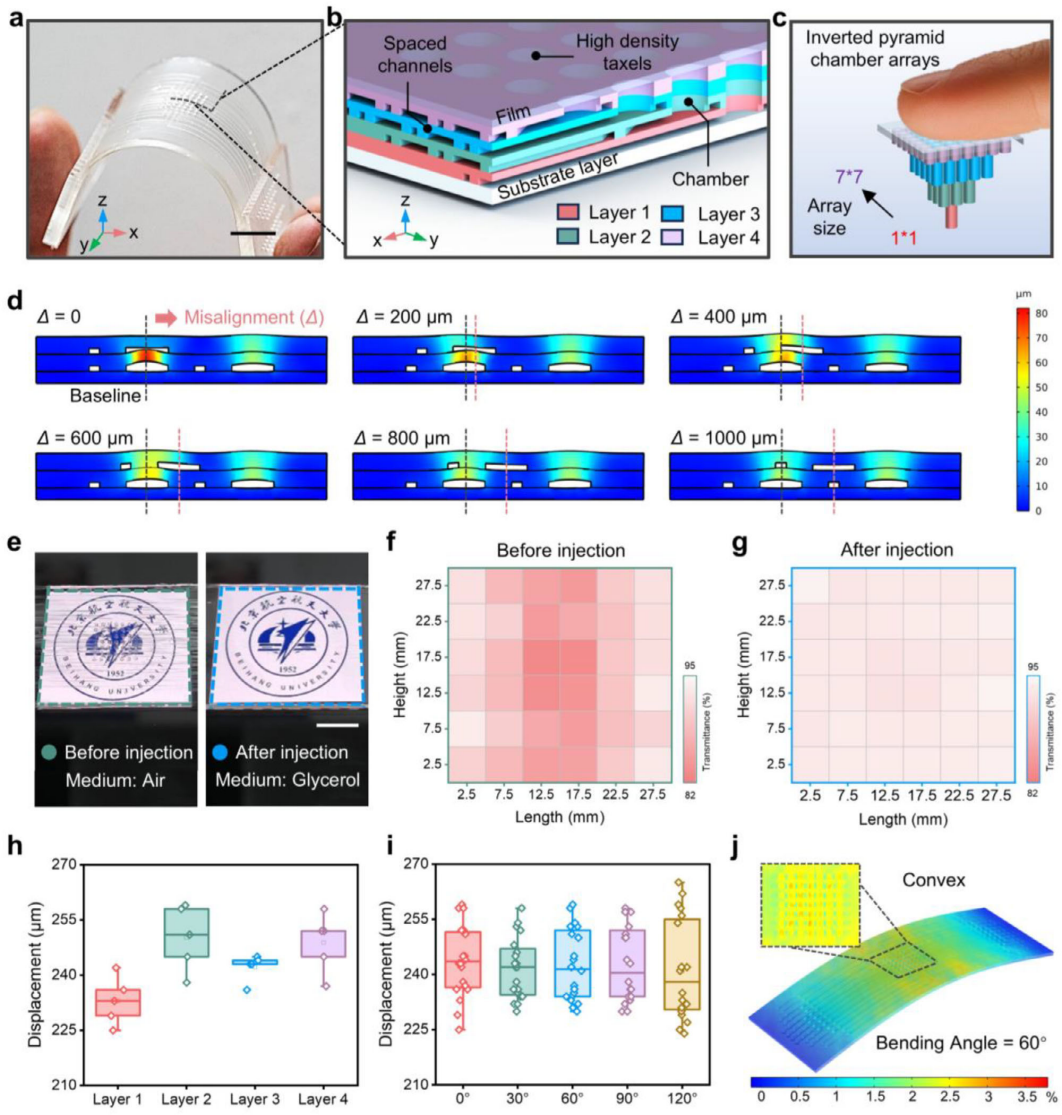
Design and characterization of the transparent microfluidic actuator array. a) Optical images of haptic interface with a 7 × 7 actuator array in the liquid‐unfilled state. Scale bar, 1 cm. b) Cross‐section view schematic illustration of the haptic interface, showing the high‐density chamber array and layered‐spaced arrangement channel system. c) Schematic diagram of a high‐density inverted pyramid distributed chamber array, chamber density: 60 units/cm^2^. d) Finite element modeling of haptic interface under different misalignment distances. Channel interference is minimized at *Δ* = 1000 µm. e) Optical image comparison of the haptic interface adhered to the touchscreen before and after injection. Scale bar, 5 mm. f,g) Transmittance distribution of the haptic interface with a 7 × 7 actuator array, (f) before and (g) after filling. Light wavelength: 400–700 nm. h) Output displacement of actuators at different layers of haptic interface. Square, mean; center line, median; box limits, upper and lower quartiles; whiskers, 1.5× interquartile range; points, outliers; *n*  =  5 independent samples. i) Relationship between the curvature of the haptic interface and the output displacement of actuators. Square, mean; center line, median; box limits, upper and lower quartiles; whiskers, 1.5×interquartile range; points, outliers; *n*  =  20 independent samples. j) The finite element analysis of surface film deformation in a haptic interface under 60° bending indicates that the maximum pre‐strain reaches 3.8%.

To render high‐fidelity haptic feedback for detailed images, we develop a high spatial resolution haptic interface composed of an array of microfluidic actuators (**Figure** **3**a). The device integrates multiple structural layers, a base layer, and a thin film, forming a scalable architecture for high‐density haptic actuation. Each structure layer contains chamber arrays with varying scales, with channels connected only to the outermost chamber (Figure 3b). By vertically stacking these layers, an inverted pyramid‐shaped chamber array and an interconnected multi‐layered channel network are established (Figure 3c; Figure S6, Supporting Information). This spatial layout enables independent and high‐density connection between chambers and channels, overcoming the constraints of conventional microfluidic designs. Unlike traditional spatial layouts, where channels and chambers are conventionally restricted to being co‐planar,^[^ ^25^, ^28^, ^29^, ^42^, ^43^, ^44^ ^]^ our approach positions them in spatially parallel layers, allowing channels to be routed efficiently without reducing chamber density (Figure S20, Supporting Information). This design significantly enhances the spatial resolution of the haptic interface while maintaining compact integration (Table S3, Supporting Information). The resulting haptic interface achieves an actuator density of 49 units/cm^2^, with a pitch of 1.5 mm and individual actuator diameter of 1 mm, exceeding the two‐point discrimination threshold of the human fingertip^[^ ^31^ ^]^ (Figure S21, Supporting Information), ensuring fine‐grained haptic rendering.

Despite its advantages, the pressure differentials between adjacent channels in multi‐layered structures may induce undesired deformation, potentially leading to crosstalk between actuators in different layers. To mitigate this effect, we implemented a misaligned channel distribution strategy, where channels in adjacent layers are deliberately offset (Figure 3b,d). Finite Element Analysis (FEA) results indicate that channel deformation decreases as the misalignment distance increases, reaching a minimum at 1000 µm (Figure 3d; Figure S22, Supporting Information). Additionally, we assessed the influence of channel height on deformation across different structural layers (Figure S23, Supporting Information). While increasing the channel height from 50 to 200 µm enhances fluid transport efficiency, it also results in greater channel deformation. Considering the trade‐off between dynamic response and structural stability, a channel height of 100 µm was selected, ensuring both efficient fluid transport and minimal structural interference. Under these optimized conditions, the microfluidic actuators exhibit rapid and stable actuation performance across varying driving frequencies and input pressures (Figure S24, Supporting Information).

To achieve independent manipulation of actuators in real‐time, a miniaturized pressure control system was developed. The system (schematic in Figure S25, Supporting Information) enables precise control of a 7 × 7 actuator array, facilitating rapid and responsive haptic feedback synchronized with visual content. The Atmega 328p microcontroller processes sensor inputs and regulates components such as pumps and valves (Figure S26, Supporting Information). Commands are transmitted from a PC or smartphone via a serial interface, with the MCU modulating the input pressure through MOSFET‐controlled pneumatic channels, thereby actuating individual microfluidic chambers with high precision. To evaluate the audible noise generated by the solenoid valves, we measured the sound pressure levels in a quiet indoor environment (Figure S27, Supporting Information). The ambient noise level was 45.51 ± 0.65 dBA, which increased to 54.57 ± 1.04 dBA upon valve activation. These results indicate that the sound produced by the solenoids is clearly perceptible but remains within an acceptable range for typical indoor use.

We then evaluated the optical transparency and actuation consistency of the microfluidic actuators across different structural layers. To assess the feasibility and effectiveness of glycerin solution as a medium for multilayer structures, we analyzed the transparency of the haptic interface before and after it was filled with a 55 wt.% glycerol solution (Figure 3e; Figure S7, Supporting Information). Prior to injection, internal chamber structures were visibly discernible, leading to visual artifacts that compromise display quality. Upon filling, the refractive index matching effect rendered the interface optically transparent, enabling clear visualization of touchscreen content. To quantify transmission uniformity, we divided the interface into 16 virtual regions and measured the transmittance (Figure 3f,g). Results indicate that the average transmittance reached 93.26%, significantly improving uniformity compared to the unfilled state. To evaluate the uniformity of the tactile interface, actuators within and across structural layers exhibited minimal differences in displacement and output force under identical input pressure, and further assessment of response time revealed similar actuation dynamics (Figure 3h; Figure S28, Supporting Information). Additionally, actuators with a diameter of 1 mm induced negligible optical distortion at 70 kPa input pressure, ensuring that touchscreen visuals remain undistorted during actuation (Figure S29, Supporting Information).

Beyond flat surfaces, the flexibility of the haptic interface allows it to conform to curved displays, broadening its applicability in next‐generation interactive devices. As shown in Figure S30 (Supporting Information), we analyzed the relationship between bending angle and actuator displacement to assess actuation consistency across different bending conditions (Figure 3i), showing that the actuator displacement varies only slightly across different angles. FEA simulations further indicate that, at a bending angle of 60°, the interface seamlessly adapts to surfaces of varying curvature, with a maximum unactuated‐state film deformation of 3.8% (Figure 3j). These results confirm that output displacement remains stable across various curved surfaces, demonstrating the interface's reliability under deformation. Finally, a user study was conducted to evaluate the impact of the haptic interface on interactive touchscreen operations, specifically tapping and swiping. As shown in Figure S31 (Supporting Information), success rates for both gestures remained above 95% and 92%, respectively, regardless of whether the haptic interface was present. These findings confirm that the haptic interface maintains high operational accuracy, ensuring seamless integration with conventional touchscreen interactions while providing rich tactile feedback.

### 1.5 Enhancing Touchscreen Interaction Immersion

**Figure 4:**
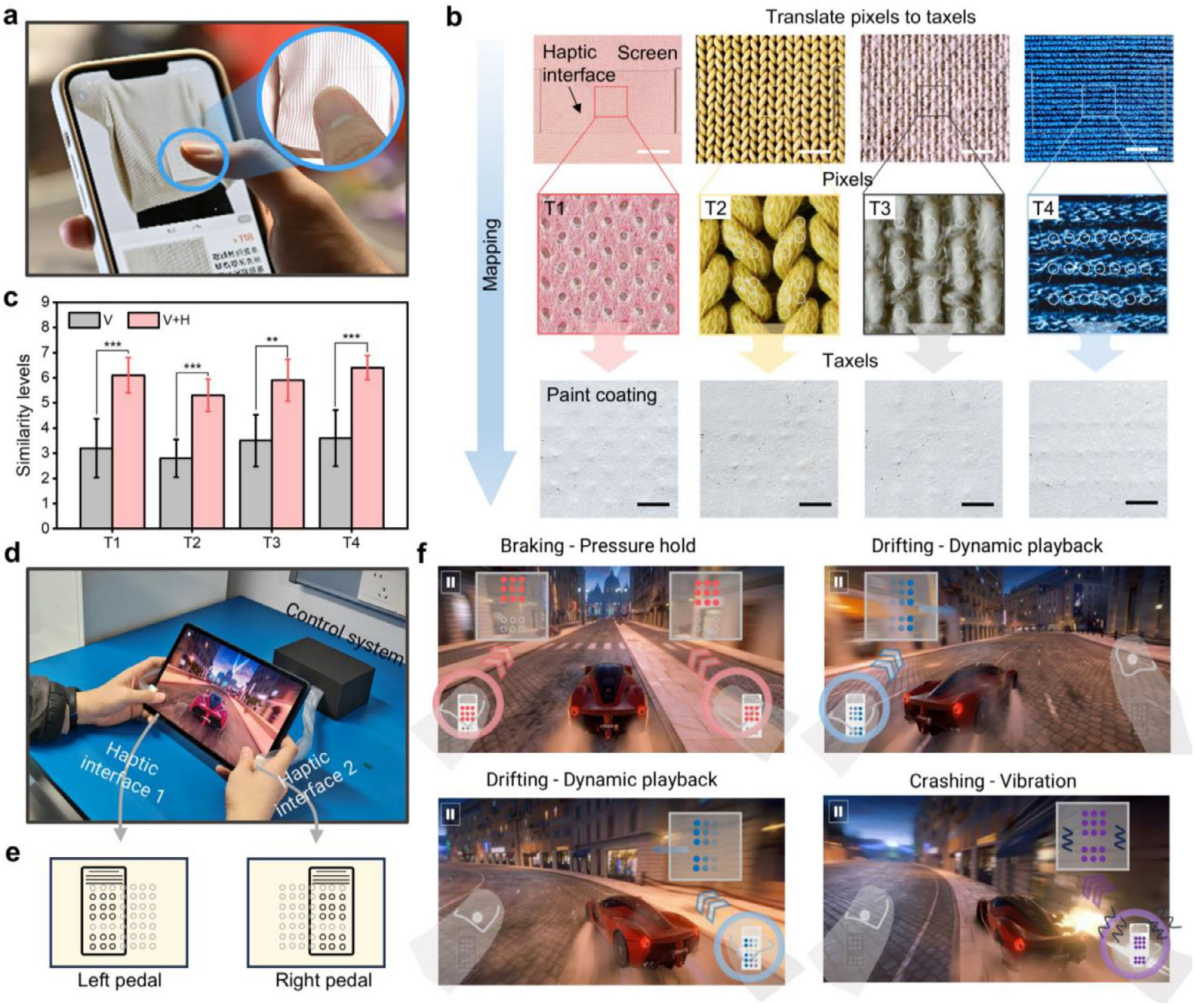
Example of applications of the haptic interface for highly immersive touchscreen interaction. a) Haptic interface for mobile online shopping. b) Demonstration of the pixel‐to‐taxel process for fabric textures. To facilitate the visualization of the transparent actuators, a thin gray coating is sprayed on the surface of the haptic device. Scale bar, 2 mm. c) Results of user study when distinguishing different fabric textures with only visual feedback and both visual feedback and haptic feedback. Bar height, mean; error bars, s.d.; *n*  = 10 independent samples. Statistical significance was assessed using paired sample *t*‐tests. d) demonstration of the haptic interface applied to touchscreen interaction in a racing game. Two independent haptic interfaces are attached to the screen, providing tactile feedback corresponding to in‐game controls. e) Schematic representation of the left and right pedal haptic interfaces, simulating brake, drift, and crash feedback. f) Various haptic feedback effects correspond to different driving scenarios: “Braking – Pressure hold” simulates resistance during braking, “Drifting–Dynamic playback” provides continuous feedback during drifts, and “Crashing–Vibration” generates impact sensations upon collision.

Blending visual and haptic feedback in touchscreen interactions significantly enhances realism and immersion in virtual environments.^[^ ^6^, ^7^ ^]^ A high‐resolution, programmable haptic interface can deliver rich tactile information that aligns with the visual content, whether it's fine textures or subtle feedback in dynamic interactions. We demonstrate this capability in an online shopping scenario, where four representative fabrics (knitted, mesh, woven, and ribbed) are rendered with lifelike textures through localized haptic actuation (**Figure** **4**a,b; Video S4, Supporting Information). By spatially mapping key image features to actuator deformations, users can explore texture details directly on the screen. To evaluate the fidelity of this haptic rendering, we conducted a user study comparing visual‐only and combined visual‐haptic conditions. As shown in Figure 4c, the integration of haptic feedback significantly improved users’ perceived similarity to real textures. These results highlight the potential of high‐resolution haptic interfaces in enhancing multisensory realism for virtual shopping experiences.

Beyond static texture rendering, this interface enables real‐time haptic augmentation of dynamic control interfaces. We implemented this in a gaming scenario by integrating tactile cues into a virtual joystick, where actuator‐driven pressure and vibration modulations provide intuitive feedback for in‐game actions (Figure 4d). For example, when braking, pressure feedback is generated beneath the interactive interface, and when a crash occurs, vibration is applied to the corresponding side of the foot pedal (Figure 4e,f; Figure S32a,b, Supporting Information). A user experiment evaluating the realism of haptic feedback (Figure S32c, Supporting Information). Participants were asked to perform a series of game actions with haptic feedback, and assess their immersion and sense of realism through a questionnaire (Table S4, Supporting Information). The results showed that when the game was provided with three types of tactile feedback, participants’ sense of immersion significantly increased. These findings suggest that haptic‐enhanced touchscreen interactions can enrich digital experiences beyond traditional audiovisual interfaces, offering greater engagement and functional benefits.

### 1.6 Improving Touchscreen Interaction Accuracy

Traditional morphable haptic devices primarily simulate simple touchscreen interactions, such as the tactile feedback of virtual buttons.^[^ ^25^, ^26^, ^27^, ^28^, ^29^, ^30^, ^31^ ^]^ In contrast, our device leverages high‐resolution and programmable haptic feedback to dynamically reshape the localized surface topography of touchscreens (e.g., automotive control panels, smart home interfaces), enabling real‐time tactile rendering of UI symbolic elements and providing precise tactile cues for essential touchscreen interactions such as sliding and rotating. The haptic interface with these features provides spatial orientation, attention guidance, and sensory substitution, making it particularly advantageous in visually demanding scenarios, such as driving or operating under strong ambient lighting.^[^ ^5^, ^32^, ^44^, ^45^ ^]^

**Figure 5:**
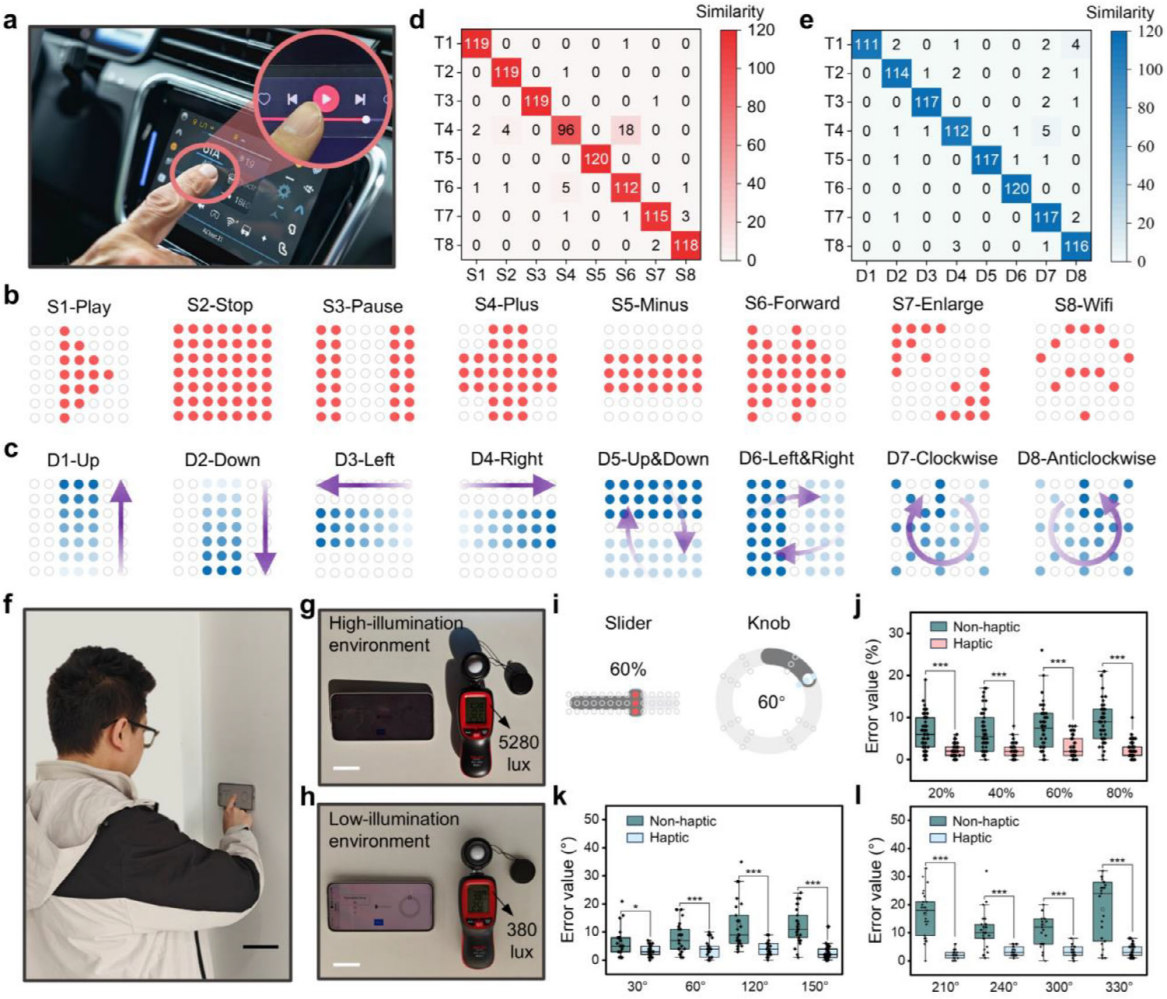
High‐resolution, programmable haptic feedback for precise interaction guidance. a) Haptic interface for a touchscreen in an intelligent car. b) Static multimedia patterns, and c) dynamic operation animation of car panels in a user study. d) Confusion matrix of participant performance on eight static multimedia patterns and e) eight dynamic operation animations. f) Photograph of a typical user interaction with a touch panel, adjusting a slider or knob via touchscreen control. Scale bar, 10 cm. g) Optical image of the touchscreen interface under high illumination (5280 lux), showing increased glare and reduced display clarity due to light scattering. h) Optical image under low illumination (380 lux), where screen content remains distinct with minimal visual artifacts. Scale bars, 3 cm. i) Schematic of touchscreen interaction tasks, including a linear slider (0–100%) and a rotary knob (0°–360°). The high‐resolution, programmable haptic interface delivers precise tactile cues, mapping interaction positions to corresponding haptic feedback locations. The figure highlights the haptic feedback positions corresponding to a 60% slider progress and a 60° knob rotation. j) Progress rate of the slider task at different target values (20%, 40%, 60%, 80%) under non‐haptic and haptic feedback conditions. Square, mean; center line, median; box limits, upper and lower quartiles; whiskers, 1.5×interquartile range; points, outliers; *n*  = 50 independent samples. Statistical significance was assessed using paired sample t‐tests. Rotation angles achieved in the knob task under k) target values (30°, 60°, 120°, 150°) and l) target values (210°, 240°, 300°, 330°) with and without haptic feedback. Four cardinal directions (90°, 180°, 270°, 360°) are omitted. Square, mean; center line, median; box limits, upper and lower quartiles; whiskers, 1.5×interquartile range; points, outliers; *n*  =  25 independent samples. Statistical significance was assessed using paired sample *t*‐tests.

To evaluate its effectiveness, we conducted a two‐part user perception study focusing on static multimedia icons and dynamic operation animations, both commonly used in smart car interfaces (**Figure** **5**a). To minimize potential confounding factors, all participants were blindfolded and wore headphones to block visual and auditory cues during the experiment. Given the visual overload drivers experience at high speeds, integrating haptic feedback can significantly enhance operational accuracy and safety.^[^ ^5^ ^]^ In the first study, we mapped eight commonly used touchscreen icons to haptic patterns (Figure 5b; Figure S33 and Video S5, Supporting Information). The confusion matrix (Figure 5d) revealed an average recognition accuracy exceeding 80%, with “Plus” and “Forward” being the most frequently confused, likely due to their similar geometric features. In the second study, we designed eight dynamic haptic animations, including four “directional cues,” two “state transitions,” and two “rotational gestures” (Figure 5c; Figure S34, Video S6, Supporting Information). Recognition accuracy for these dynamic animations exceeded 92% (Figure 5e), demonstrating that dynamic tactile cues at the fingertips allow users to recognize directions or states.

In high‐light environments, visual information can be compromised by glare or reflections, reducing interaction accuracy, which is a common issue for household touch panels (Figure 5f–h). To address this challenge, we evaluated the effectiveness of a high‐resolution, programmable haptic interface in assisting interactive control tasks involving sliders and rotary knobs under such conditions. Participants performed these tasks under both haptic and non‐haptic conditions in a high‐illumination environment (5280 lux), simulating scenarios with limited visual cues (Figure 5g). The interactive tasks included adjusting a linear slider within a 0–100% range and rotating a knob to a specified angle within a 0°–360° range (Figure 5i). The haptic interface provided precise spatial tactile cues, mapping the position of interactive components to corresponding haptic feedback regions. Experimental results showed that, compared to the non‐haptic condition, haptic feedback significantly improved the completion rate of the slider task (Figure 5j) and enhanced angular accuracy in the rotary knob task (Figure 5k,l). Furthermore, during repeated interactions, haptic feedback effectively reduced operational variance, enabling users to reach target values more consistently while minimizing adjustment errors and unnecessary interactions. These findings demonstrate that high‐resolution, programmable haptic interfaces can provide precise feedback mechanisms for sliders and rotary knobs, maintaining efficient interaction performance even in visually constrained or complex lighting conditions. This highlights the potential of haptic feedback technology for applications in smart home interfaces, in‐vehicle touch controls, and other scenarios requiring high‐precision interactions.

## 2 Conclusion

Through the integration of transparency, high resolution, and dynamic programmability, the morphable haptic interface introduced here transforms touchscreen interactions. This system establishes a foundation for the harmonious integration of fine‐grained tactile feedback into interactive displays, enabling precise, localized modulation of surface topography without compromising visual clarity. Leveraging a multilayer transparent microfluidic actuator array, it delivers high‐resolution tactile feedback while maintaining uniform optical transmission across the screen. Its independently addressable actuators enable scalable, high‐fidelity tactile rendering of localized surface features in real time. Furthermore, the system dynamically encodes tactile stimuli, adjusting actuation sequences to distinguish spatially and temporally varying patterns, thereby enhancing perceptual differentiation in interactive tasks. These capabilities collectively expand the scope of haptic interactions on touchscreen platforms. By enabling real‐time programmable tactile guidance, interactive texture rendering, and immersive control feedback, this technology establishes a scalable approach for seamlessly integrating high‐resolution haptics into touch‐based interfaces, unlocking new possibilities in automotive interfaces, social media, virtual environments, and beyond (Figure S35, Supporting Information).

## 3 Experimental Section

### Fabrication of Microchannel Structural Layers

The microchannel structural layers were fabricated using soft lithography (Figure S3, Supporting Information). A 5‐inch silicon wafer was first cleaned in a 3:1 solution of H_2_SO_4_ and H_2_O_2_ for 20 minutes, rinsed with deionized water, dried with nitrogen, and heated to 120 °C for 20 minutes. SU‐8 photoresist was spin‐coated onto the wafer to achieve a 100‐µm‐thick layer, using a spin sequence of 500 rpm for 5 s followed by 2200 rpm for 20 s. After a sequential pre‐baking process at 65 and 95 °C, the wafer was exposed to UV light through a reverse mask (10 mJ cm^−^^2^ for 24 s), followed by post‐exposure baking and development in an SU‐8 developer. A final hard bake at 160 °C enhanced pattern durability and adhesion. To create the PDMS microchannel layers, the base and curing agent were mixed at a 10:1 ratio, degassed, and poured into the mold. After an additional degassing step, the PDMS was cured at 85 °C for 20 minutes and then carefully peeled off to reveal the patterned microchannels. The PDMS layers were then trimmed to the specified design dimensions using a precision cutter, yielding four structural layers, each with a thickness of 300 µm (Figure S4, Supporting Information).

### Fabrication of Actuator Array

As illustrated in Figure S5 (Supporting Information), actuator cavities, fluid inlets, and exhaust ports were created in structural layer 1 by perforation using a 1‐mm punch. Structural layers 1 and 2 were ultrasonically cleaned in anhydrous ethanol for 10 minutes, dried, and pre‐aligned without contact. The layers were then plasma‐activated at 700 W for 60 seconds, aligned, and pressed together, followed by thermal curing at 85 °C for 120 minutes to form a permanent bond, creating composite layers 1–2. This process was repeated iteratively: perforation of layers 1–2, bonding to layer 3 (forming layer 1‐2‐3), additional perforation, and bonding to layer 4 (forming layer 1‐2‐3‐4). The final composite structure was bonded to a 200‐µm‐thick PDMS substrate (prepared with a 5:1 base‐to‐curing‐agent ratio), ensuring complete sealing of all 49 microchannels. To finalize the actuator array, a 100‐µm‐thick PDMS film (20:1 ratio) was spin‐coated and bonded onto the upper surface of the structural layers, enclosing the exposed gas chambers.

### Injection and Encapsulation of Liquid Media

A 55 wt.% glycerin aqueous solution was slowly injected through the inlet using a flat‐tip syringe while allowing air to escape through the outlet (Video S1, Supporting Information). To prevent air bubble entrapment within the actuator, the injection rate was carefully reduced as the solution entered the driver. Once the solution began to overflow from the outlet, the injection was halted, and the microfluidic outlet was sealed with a rubber stopper. This process was repeated for all 49 actuators, ensuring complete replacement of the liquid medium and yielding an optically transparent haptic interface (Figure S8, Supporting Information).

### Optical Characteristics

The refractive index of the fluid medium was measured using an Abbe refractometer (2WAJ, Shanghai Optical Instrument) connected to a thermostatic water bath to ensure a stable temperature. Glycerol solutions with mass fractions ranging from 0% to 100% (in 5% increments) were tested at room temperature (25 °C). For the 55% glycerol solution, refractive indices were recorded at five different temperatures (5 to 45 °C, in 10 °C increments), with a 10‐minute stabilization period before each measurement. Each condition was tested independently three times, and the mean refractive index with variance was documented. Additionally, light transmission of the actuators and the transparent haptic interface—both before and after glycerol solution injection—was analyzed using a colorimetric haze meter (LS155, Linshang Technology). The spectral transmittance was measured across the visible wavelength range (400–700 nm). To evaluate uniformity, the central region of the transparent haptic interface containing the actuator array was divided into a 6 × 6 grid of 5 mm × 5 mm areas, and the spectral transmittance of each area was recorded before and after fluid injection.

### Haptic Feedback Characteristics

The actuator's output displacement was measured using a non‐contact spectral confocal displacement sensor (LTC 4000F, Hongchuan Technology) mounted on a vertically adjustable stand, with a maximum sampling frequency of 10 kHz. The actuator was fixed on a precision XYZ micro‐positioning stage (Hengyang Optical), and a microfluidic pressure control system was used to apply specific air pressures. Real‐time measurements of both static and high‐frequency displacement responses of the actuator's film were recorded under varying driving pressures, which were adjusted based on different PDMS ratios and film thicknesses. Blocked force measurements were conducted using a contact‐based method. A parallel cantilever beam force sensor (AR‐WM10S, Arizon Technology) was mounted on a vertical stand, and the Z‐axis position of the micro‐positioning stage was adjusted to bring the actuator into contact with the sensor. After calibrating the actuator film's contact displacement, a predefined driving pressure was applied using the microfluidic pressure control system. The blocked force under different pressures was measured in real time and recorded via a pressure acquisition card with a maximum sampling frequency of 1 kHz.

### Mechanical Simulation of Films

The study conducted finite element analysis using the commercial software COMSOL Multiphysics to study the mechanical characteristics of the actuator film under increasing pressure. The strain distribution of the film under different diameters (1–3 mm), thicknesses (100, 150, 200 µm), and modulus was discussed. The PDMS film was modeled using tetrahedral elements with a minimum element size of 100 µm to ensure mesh convergence and simulation accuracy. In the finite element analysis, PDMS was specified as a hyperelastic material using the Mooney–Rivlin energy potential model, while other materials were designated as exhibiting elastic behavior. The elastic modulus (*E*) and Poisson's ratio (*ν*) used in the simulations are as follows: for PDMS (10:1), *E*_PDMS(10:1)_ = 1.8 MPa and *ν*_PDMS(10:1)_ = 0.46; for PDMS (20:1), *E*_PDMS(20:1)_ = 1 MPa and *ν*_PDMS(20:1)_ = 0.48; for PDMS (30:1), E_PDMS(30:1)_ = 0.35 MPa and *ν*_PDMS(30:1)_ = 0.49.

### Absolute Threshold of Driving Pressure

A pressure absolute threshold experiment was conducted using the proposed haptic interface to understand if participants could perceive the tactile stimuli generated by the drivers (Figure S19, Supporting Information). Eleven participants (seven males and four females, aged between 23 and 28) from Beihang University were recruited to participate in this tactile test, all of whom were right‐handed. The study was approved by the Ethics Committee of Beihang University for Biological and Medical Research (Approval No. BM20230035), and written informed consent was obtained from all participants prior to their involvement. The experimental setup was fixed on the tabletop, and participants placed their index fingers in the texture presentation area. Participants were allowed to explore by sliding their fingers during the experiment. The psychophysical experiment employed the method of constant stimuli, generating thresholds at 79.4% of the psychometric function. Each participant was required to participate in two sets of trials, each consisting of ≈25 trials. After each set of trials, participants were asked to rest for two minutes. Throughout the entire experiment, participants were blindfolded to prevent interference from visual cues. Larger step sizes were used in the initial reversals to quickly approach the estimated threshold level. After more than eight reversals, smaller step sizes were used to improve the accuracy of threshold estimation until pressure values converged (*T*_i_ ≤ 1 kPa) or reached 50 trials. If convergence was not achieved after 50 trials, the number of trials was increased accordingly until convergence was reached. Finally, the pressure values at each reversal were found. To eliminate the influence of unstable early reversals, the pressure values of the last 8 reversals were averaged to obtain an average threshold pressure value.

### Mechanical Simulation of Channels

The study used COMSOL Multiphysics for finite element analysis to study the interference between adjacent channels by varying the misalignment distance and height of the channels. The channel was modeled using tetrahedral elements with a minimum element size of 100 µm to ensure mesh convergence and simulation accuracy. In the simulation, the elastic modulus (*E*) and Poisson's ratio (*ν*) used for PDMS were as follows: for PDMS (5:1), *E*_PDMS(5:1)_ = 4 MPa and *ν*_PDMS(5:1)_ = 0.45; for PDMS (10:1), *E*_PDMS(10:1)_ = 1.8 MPa and *ν*_PDMS(10:1)_ = 0.46.

### Control System

The 7 × 7 actuator array is independently controlled by a hydraulic pressure system comprising a control circuit, an air pump, an air tank, an air pressure sensor, and solenoid valves (two‐position three‐way valves). Pressurized air is delivered to the microfluidic inlets of the actuators, where the inlet fluid is displaced by air pressure while the outlet remains sealed with a rubber plug, inducing membrane deformation for independent actuation. The system is governed by an MCU (Atmega328p‐AU), which regulates the air pump (KVP04‐1.1‐12, Shanghai Kamor Fluid Technology) via a MOSFET (AO3400) for inflation and speed control through PWM modulation. A gauge pressure sensor (GZP6847A101KPP50K, 0–40 kPa, ±1% Span accuracy) monitors the tank pressure, transmitting a voltage signal that is processed by the MCU. To manage the large number of solenoid valves, six I/O expanders (TCA9554PWR) communicate with the MCU via the I^2^C protocol, providing 48 control signals for the 51 solenoid valves through MOSFETs (NDC7002N). Specifically, two solenoid valves (S070C‐6BG‐32) regulate the tank's inlet and exhaust, while 49 solenoid valves (S070M‐6BG‐32) control independent air pressure outputs. The system allows external control via a host PC, which can set output pressure and activate solenoid operations through a serial port. Additionally, the MCU interfaces with smartphones via Bluetooth (WH‐BLE103A). A closed‐loop pressure control mechanism is maintained, dynamically adjusting the system based on real‐time feedback from the pressure sensor.

### Audible Noise Test

To evaluate the audible noise generated by solenoid valves, a digital sound level meter (Deli 333211, China) was placed 20 cm in front of the valve. The test was conducted in a quiet indoor environment with ambient noise levels below 50 dBA. Noise levels were recorded at 2 Hz over a 15‐second duration. During the first and last 5 seconds, the valve remained closed to capture background noise. In the middle 5 seconds, the solenoid valve was driven with a 10 Hz on‐off cycle, exceeding the acoustic sampling frequency. A total of 30 data points were collected, and the experiment was repeated to ensure reliability.

### User Study of Progress Bar Interaction

To evaluate the role of high‐resolution haptic feedback in fine‐tuning adjustments under limited visual cues, an experiment was conducted using a customized mobile UI interface and two tailored haptic interfaces. Five volunteers (three males and two females, aged 23–29) participated in the study. To eliminate visual and auditory cues, participants were blindfolded and wore headphones throughout the experiment. The study was approved by the Ethics Committee of Beihang University for Biological and Medical Research (Approval No. BM20230035), and written informed consent was obtained from all participants prior to their involvement. A 3 × 15 haptic actuator array was attached to the linear progress bar, while a circular actuator array was applied to the circular progress bar. The experiment was conducted indoors under direct sunlight, where only the blurred outline of the progress bar was visible on the screen. Participants were instructed to slide the progress bar button from an initial position to a target position and then report the actual final position and the time required to complete the task. Each progress bar was tested under two conditions: without haptic feedback (haptic interface attached but not actuated) and with haptic feedback (actuators at the target position activated). For the linear progress bar, four target positions (20%, 40%, 60%, and 80%) were randomly selected, each repeated 10 times in a randomized order, resulting in a total of 50 trials per condition. For the circular progress bar, eight target positions (30°, 60°, 120°, 150°, 210°, 240°, 300°, and 330°) were randomly selected, each repeated five times in a randomized order, also totaling 25 trials per condition. By comparing the experimental results between the two conditions, the effectiveness of high‐resolution haptic feedback in enhancing fine‐tuning accuracy and control in visually challenging environments was assessed.

### User Study of Tactile Pattern Discrimination

In the experiments, a 7 × 7 haptic interface array was used to assess participants’ ability to recognize various static and dynamic tactile patterns. Twelve volunteers (eight males and four females, aged 23–28) took part in this study. To eliminate visual and auditory cues, participants were blindfolded and wore headphones throughout the experiment. The study was approved by the Ethics Committee of Beihang University for Biological and Medical Research (Approval No. BM20230035), and written informed consent was obtained from all participants prior to their involvement. To eliminate visual and auditory cues, participants were blindfolded and wore headphones throughout the experiment. In the static tactile pattern recognition test, individual actuators in the array produced eight common multimedia tactile patterns (Figure 5b,c), each presented 20 times in random order for a total of 160 presentations. Participants had 3 s to identify the tactile pattern they perceived. By comparing the presented patterns with the responses, the transparent haptic interface's performance in delivering static tactile feedback was evaluated. For the dynamic tactile animation recognition test, pre‐programmed sequences were applied to the actuator array to generate eight distinct dynamic tactile animations, each driven at 3.33 Hz with a 3‐second playback duration. Each animation was presented 20 times in random order, totaling 160 presentations. After each playback, participants reported the category of the tactile animation they felt. This data helped assess the interface's ability to convey dynamic tactile feedback effectively.

### Statistical Analysis

All data were analyzed using Origin 2021 software. All results were shown as means ± SEM. The statistical methods used are indicated in the figure legends. Significance was determined as *p < 0.05, **p < 0.01, ***p < 0.001, and ****p < 0.0001. The experiments were repeated independently multiple times, as indicated in the figure legends.

## Conflict of Interest

The authors declare no conflict of interest.

## Author Contributions

B.S., Y.G., and Y.W. contributed equally to this work. Z.D., X.Y., and D.W. supervised this study. B.S. and Y. G. conceived the idea and directed this project. B.S., P.Z., Y.W., and Y.L. conducted the main experiments. B.S., Y.G., and P.Z. designed the microfluidic haptic interface and developed the refractive index‐matching technique. Y.W. and L.H. carried out experiments on user study. Z.D., B.S., and Y.W. analyzed the data and developed the theoretical model for the actuator. B.S. and Y.G. drafted the manuscript. Y.W., W.G., Y.Z., Z.D., X.Y., and D.W. reviewed and revised the manuscript.

## Supporting information



Supporting Information

Supplemental Video 1

Supplemental Video 2

Supplemental Video 3

Supplemental Video 4

Supplemental Video 5

Supplemental Video 6

## Data Availability

Data sharing is not applicable to this article as no new data were created or analyzed in this study.
