# Supplementary material for: Fully Transparent Haptic Interface for High‐Resolution Tactile Feedback on Touchscreens
Source: Adv Sci (Weinh). 2025 Aug 19;12(42):e11874. doi: 10.1002/advs.202511874 (PMC12622511; doi:10.1002/advs.202511874)
Supplement: Supplementary file 1 — Supporting Information [file ADVS-12-e11874-s001.docx]

Supplementary Information

**Fully Transparent Haptic Interface for High-Resolution Tactile Feedback**

**on Touchscreens**

Boxue Shan^1,2,†^, Yuan Guo^3,†^, Yun Wang^1,†^, Pengbo Zhao^1,2^, Yiheng Wang^1,2^, Zemin Wang^1,2^, Liwen He^2^, Yan Liu^2^, Yibin Wang^2^, Weidong Guo^1,2^, Yuru Zhang^1,2^, Zhaohe Dai^4,*^, Xinge Yu^3,*^ and Dangxiao Wang^1,2,*^

^1^State Key Laboratory of Virtual Reality Technology and Systems, Beihang University, Beijing, China

^2^Robotics Institute, School of Mechanical Engineering and Automation, Beihang University, Beijing, China.

^3^Department of Biomedical Engineering, City University of Hong Kong, Hong Kong SAR 999077, China.

^4^Department of Mechanics and Engineering Science, College of Engineering, Peking University, Beijing, China.

†These authors contributed equally to this work.

*Corresponding authors. Email: daizh@pku.edu.cn, xingeyu@cityu.edu.hk, hapticwang@buaa.edu.cn

**This PDF file includes:**

Supplementary Texts 1 to 3

Supplementary Figs. 1 to 35

Supplementary Tables 1 to 4

**Other Supplementary Materials for this manuscript include the following:**

Supplementary Videos 1 to 6

Supplementary Text 1

Comparison of spatial resolution and optical properties of our work and recently reported morphable haptic interfaces.

Morphable haptic devices have seen significant advancements in recent years, particularly in the development of actuator mechanisms capable of generating diverse tactile stimuli^1-7^. However, existing morphable haptic devices remain constrained by fundamental trade-offs between spatial resolution, visual transparency, and integration fidelity with interactive displays. These limitations hinder their ability to provide high-fidelity, dynamically programmable tactile feedback while maintaining seamless visual interaction on touchscreens. Here, we introduce a microfluidic-based haptic interface that not only achieves high-resolution tactile feedback aligned with fine image details but also ensures optical transparency, overcoming key challenges in haptic-visual integration.

Morphable haptic devices create physical surface deformations to modulate tactile perception, offering a direct and programmable method for generating high-fidelity haptic feedback. However, as summarized in Supplementary Table 1, existing implementations face critical challenges, including limited taxel density, restricted spatial resolution, and unwanted optical obstructions when integrated with displays. Our microfluidic haptic interface addresses these constraints by embedding dozens of densely packed actuators within a fingertip-sized area, significantly increasing the spatial resolution of tactile feedback. Compared to previously reported fluidic haptic systems (Supplementary Table 3), our design miniaturizes individual actuators and optimizes the fluidic network architecture, reducing the overall footprint without compromising actuation performance. Despite the challenges associated with high-density actuator integration, our system mitigates performance degradation through an optimized routing strategy, ensuring rapid, robust, and tunable tactile feedback.

Another key innovation of our approach lies in its ability to preserve optical clarity while achieving high-resolution haptic actuation. Unlike conventional haptic devices that introduce light scattering, refraction artifacts, or structural occlusion, we leverage transparent elastomeric materials (e.g., PDMS) and a glycerol-based fluid medium with precisely tuned refractive index matching to minimize optical distortions. This strategy significantly reduces scattering and reflection, enabling seamless haptic-visual integration with high-resolution touchscreen displays.

In summary, while earlier work has made notable strides in developing various forms of haptic feedback, our system uniquely combines the advantages of high-resolution tactile feedback, transparency, and performance stability, positioning it as an ideal candidate for next-generation haptic interfaces that demand both visual and tactile integration.

Supplementary Text 2

Theoretical model of actuator output displacement based on linear elasticity theory.

1. Assumptions and Simplifications

Before delving into the detailed analysis of film deformation under pressure, it is crucial to outline the assumptions and simplifications that underpin this model:

Thin Film Approximation: The thickness t of the film is significantly smaller than its radius of curvature R (i.e., $t\ll R$). This allows us to neglect higher-order terms in the equations.

Linear Elasticity: The strain $\epsilon_{E}$must remain within a small limit, specifically $\epsilon_{E}\leq0.25$, to ensure linear behavior.

Isotropic Material: The material properties such as Young’s modulus $E$ and Poisson’s ratio $v$ are assumed to be constant throughout the material.

Small Deflections: The analysis neglects geometric nonlinearity, focusing instead on small deflections that simplify the governing equations.

Neglect Higher-Order Terms: We limit our focus to linear effects, ensuring that the analysis remains tractable.

2. Linearized Theory

**Stretching**

The axial strain induced by the film’s expansion can be approximated as:

$$\begin{aligned} \epsilon_{E}\approx\frac{h^{2}}{R^{2}}\leq0.25\#\text{(1)} \end{aligned}$$

This relationship indicates how the film’s height $h$ influences the overall strain in response to applied pressure.

**Bending**

The bending of the film contributes to the overall deformation, characterized by:

$$\begin{aligned} B=\frac{{Et}^{3}}{{12(1-v}^{2})}\leq0.25\#\text{(2)} \end{aligned}$$

This expression relates the bending stiffness $B$ to the film’s thickness $t$ and material properties. The pressure $P$ experienced by the film can be expressed as:

$$\begin{aligned} P=64B\frac{h}{R^{4}}+A\left( v \right)Et\frac{h^{3}}{R^{2}}\#\text{(3)} \end{aligned}$$

Here, $A(v)$ accounts for the influence of Poisson’s ratio on the bending response, and the two terms reflect the contributions from both bending and stretching.

**Pressure-Deformation Relation**

Integrating the previous considerations leads us to a critical relationship for pressure-induced deformation:

$$\begin{aligned} \frac{PR^{4}}{t^{4}}=\frac{32}{3}E\left( \frac{h}{t} \right)+4.74E\left( \frac{h}{t} \right)^{3}\#\text{(4)} \end{aligned}$$

This equation connects the applied pressure $P$ with the deformation characteristics of the thin film, emphasizing the dependence on both height-to-thickness ratio $\frac{h}{t}$ and material stiffness.

3. Shear Effects

Shear forces also play a significant role in the deformation behavior of the film.

**Shear Correction**

The contribution from shear to the film height h can be expressed as:

$$\begin{aligned} h\approx\frac{PR^{4}}{64B}\left( 1+\frac{4t^{2}}{vR^{2}} \right)\#\text{(5)} \end{aligned}$$

This relation captures the interplay between pressure and shear deformation, adjusting for the effects of Poisson’s ratio.

**Refined Pressure-Deformation Relation**

Incorporating shear effects leads to a refined expression for pressure:

$$\begin{aligned} P\approx\frac{64B\frac{h}{R^{4}}}{1+\frac{8t^{2}}{R^{2}}}+4.74Et\frac{h^{3}}{R^{2}}\#\text{(6)} \end{aligned}$$

This equation further clarifies how shear modifies the pressure-response relationship, highlighting the dependency on both film geometry and material properties.

**Final Expression**

Ultimately, the complete pressure-deformation relationship can be summarized as:

$$\begin{aligned} \frac{PR^{4}}{t^{4}}=\frac{32E}{3\left( 1+\frac{8t^{2}}{R^{2}} \right)}\left( \frac{h}{t} \right)+4.74E\left( \frac{h}{t} \right)^{3}\#\text{(7)} \end{aligned}$$

Supplementary Text 3

Comparison with recently reported microfluidic haptic system structural designs.

Existing fluid-driven tactile displays utilizing microscopic morphological changes can be classified based on the relative arrangement of chambers and channels into two categories: chamber-channel homogeneous distribution and chamber-channel layered distribution (Supplementary Table 3). The chamber-channel homogeneous distribution structure, characterized by the interleaved arrangement of air chambers and channels within a single layer, enhances the compactness of the system (Supplementary Fig. 20a)^5,8,9^. However, the interference between air chambers and channels in this single layer limits the maximum alignment density of the air chamber arrays, leading to a lower spatial resolution (4-20 mm). Conversely, the chamber-channel layered distribution structure mitigates the spatial congestion caused by the co-location of channels and chambers within a single layer (Supplementary Fig. 20b)^10-12^, thus providing an advantage in terms of spatial resolution.

Currently, most fluid-driven tactile displays exhibit a spatial resolution equal to or exceeding 2.5 mm (Supplementary Table 3), which still represents a significant gap compared to the spatial tactile resolution of the human fingertip. This disparity primarily arises from the wiring challenges posed by the high-density arrangement of actuators. Although the chamber-channel layered distribution structure alleviates spatial interference between air chambers and channels, mutual interference among channels remains significant.

We evaluated and compared the effect of channel cross-sectional width on the integration density of microfluidic actuators under three routing methods and established the following formula: equation.

For chamber-channel homogeneous distribution:

$$\begin{aligned} D_{a}= R+\left( N+1 \right)w+2d_{c}+{\left( N-1 \right)d}_{l}\#\text{(8)} \end{aligned}$$

For chamber-channel layered distribution:

$$\begin{aligned} D_{b}=Max\left[ R+d_{c},\left( N+1 \right)\left( w+d_{l} \right) \right]\#\text{(9)} \end{aligned}$$

For inverted pyramid-shaped chamber arrays and 3D misaligned pipe connections (our work):

$$\begin{aligned} D_{c}=Max\left[ R+d_{c},w+d_{l} \right]\#\text{(10)} \end{aligned}$$

where $D_{i}$ refers to the center distance between adjacent chambers, $R$ refers to the radius of the chamber, $N$ refers to the number of channels between adjacent chambers, $w$ refers to the width of the channel, $d_{c}$ refers to the wall thickness between chambers or between chambers and channels, and $d_{l}$ refers to the wall thickness between channels.

As shown in Supplementary Fig. 20c, we propose a layered routing approach for the fluidic channels that significantly enhances the chamber arrangement density while maintaining separation (Supplementary Figs. 20d, 20e). Moreover, the staggered configuration of the channels significantly reduces interference between them (Supplementary Fig. 22), enabling the high-density integration of chamber arrays at a spatial resolution of 1.5 mm within a 1.5 mm thick tactile interface, while achieving one-to-one interconnections between chambers and channels. This independent pressure delivery capability of the chambers is crucial for the programmable control and customizable display of tactile interface shapes.


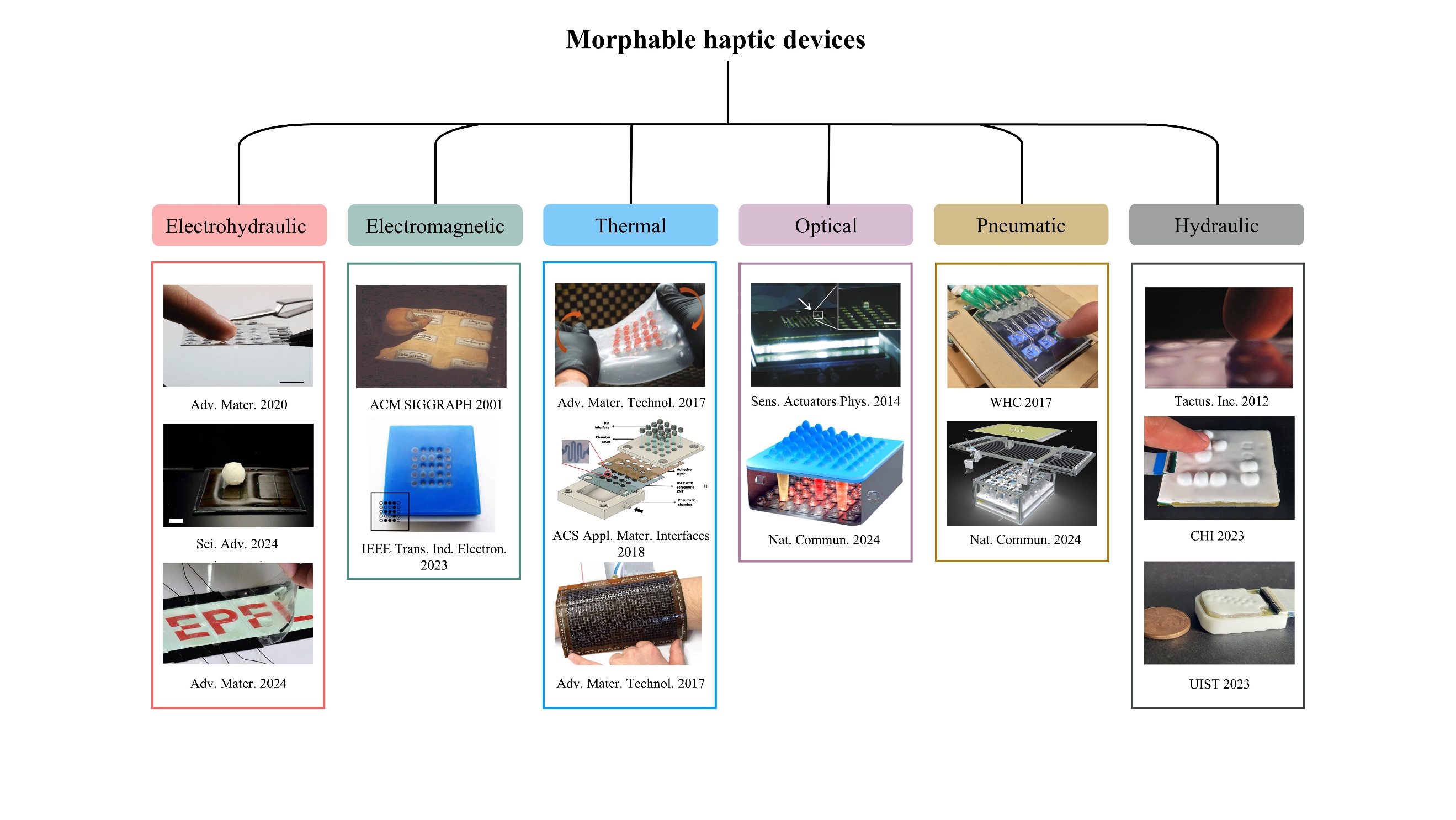


Supplementary Fig. 1 | Recent advances in morphable haptic devices. These haptic interfaces are based on a variety of actuation principles, including electrohydraulic, electromagnetic, thermal, optical, pneumatic, and hydraulic. All source images used in this figure have been obtained with the necessary copyright permissions from the original publishers.


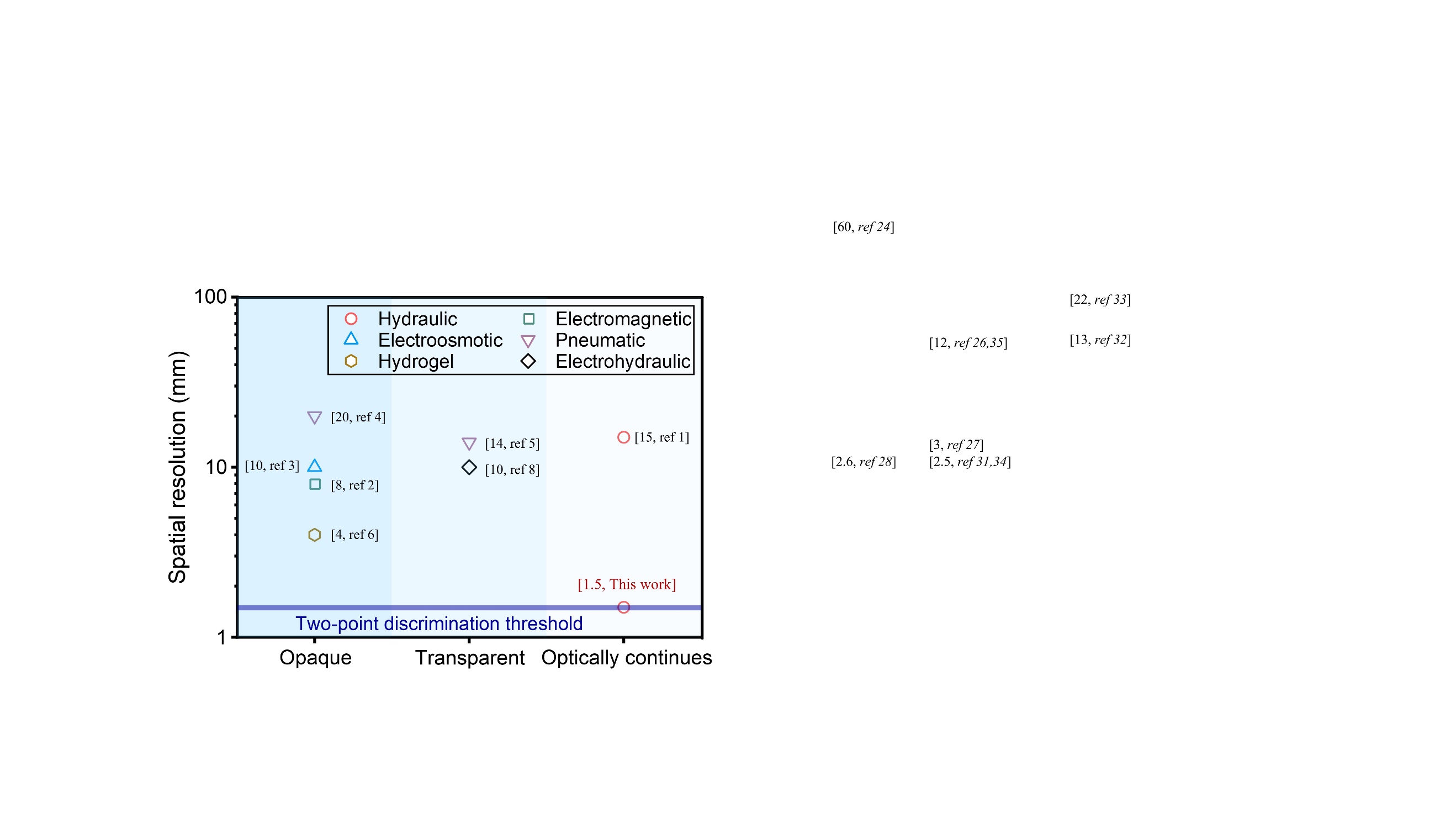


Supplementary Fig. 2 | Performance comparison of morphable haptic devices for touchscreens. The horizontal axis represents the optical performance and the vertical axis represents the spatial resolution. Spatial resolution was estimated based on the center-to-center distance (pitch) between adjacent actuators. The invisible internal structure (optically continues) makes the haptic interface very suitable for integration with visual display devices. The blue strip represents the two-point resolution threshold, which is the minimum distance between two points in space that the fingertip can distinguish (~1.5 mm).


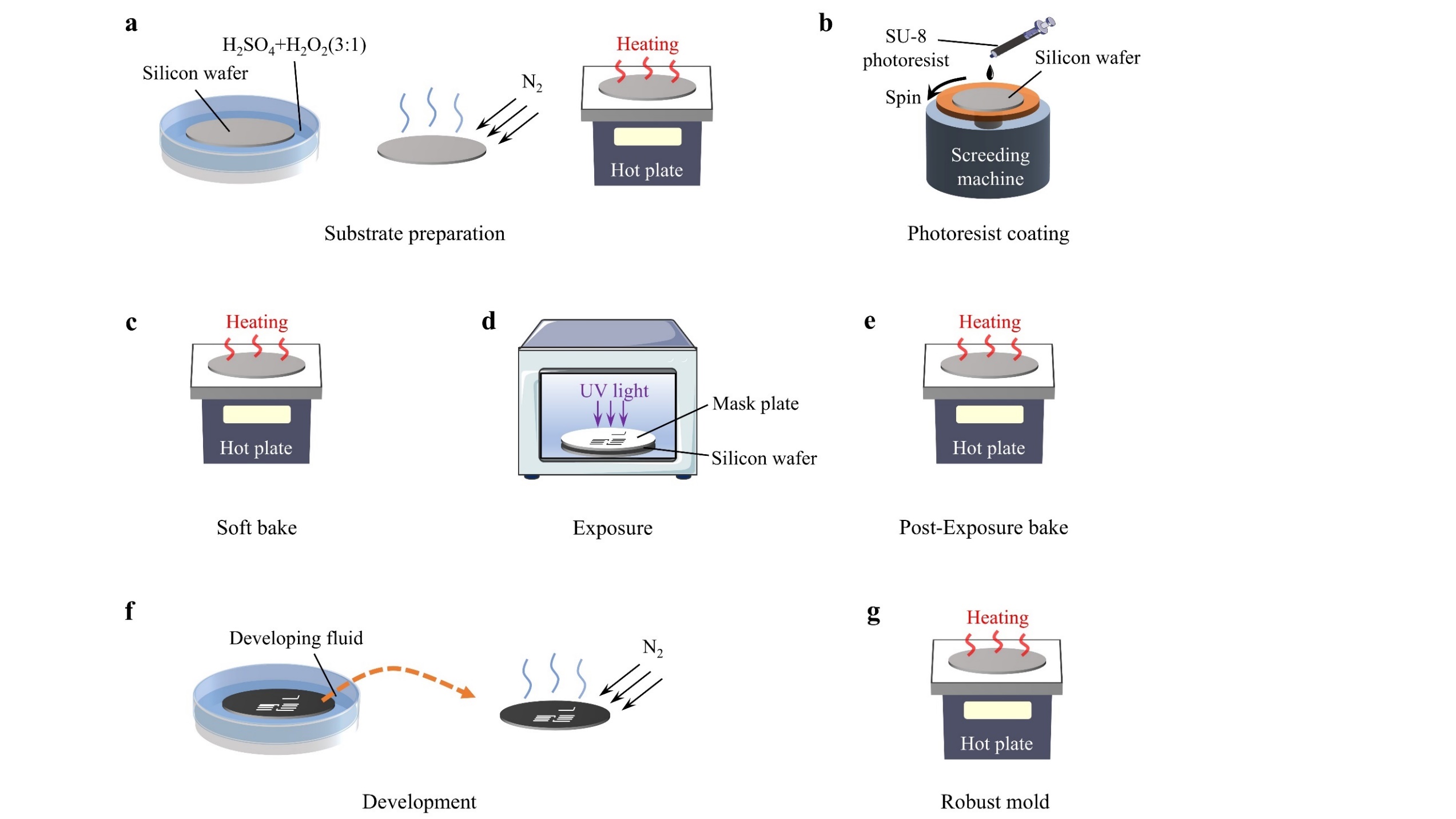


Supplementary Fig. 3 | Fabrication process of the microchannel mold. The main process includes a, substrate preparation. b, photoresist coating. c, soft baking. d, exposure. e, post-exposure baking. f, development. g, solid mold.


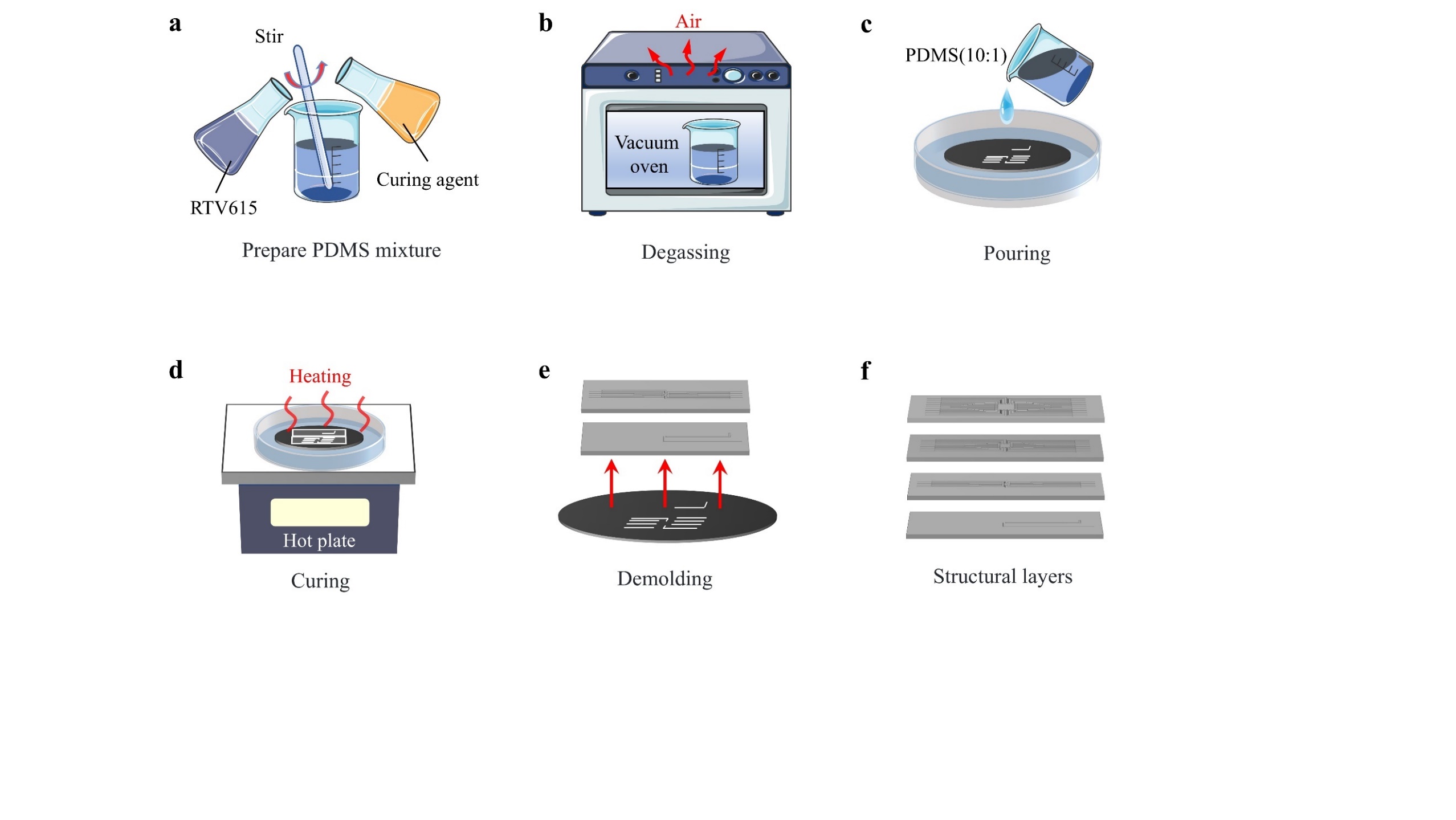


Supplementary Fig. 4 | Fabrication process of microchannel structure layers. The main process includes a, prepare PDMS mixture. b, degassing. c, pouring. d, curing. e, demolding. f, cutting.


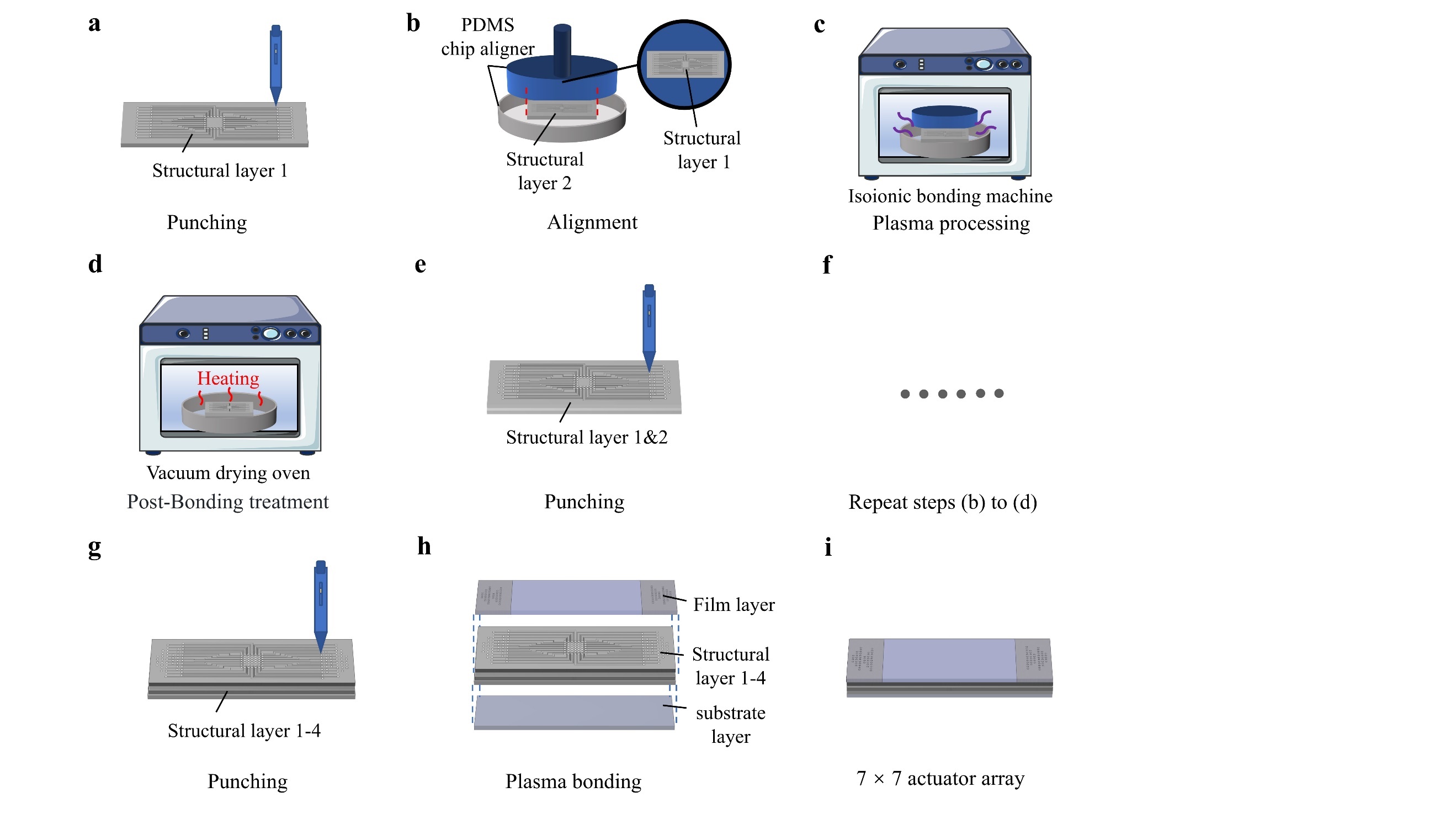


Supplementary Fig. 5 | Fabrication process of the haptic interface with multilayer structures. The main process includes ordered (a, e, g) punching and (c, f, h) bonding.


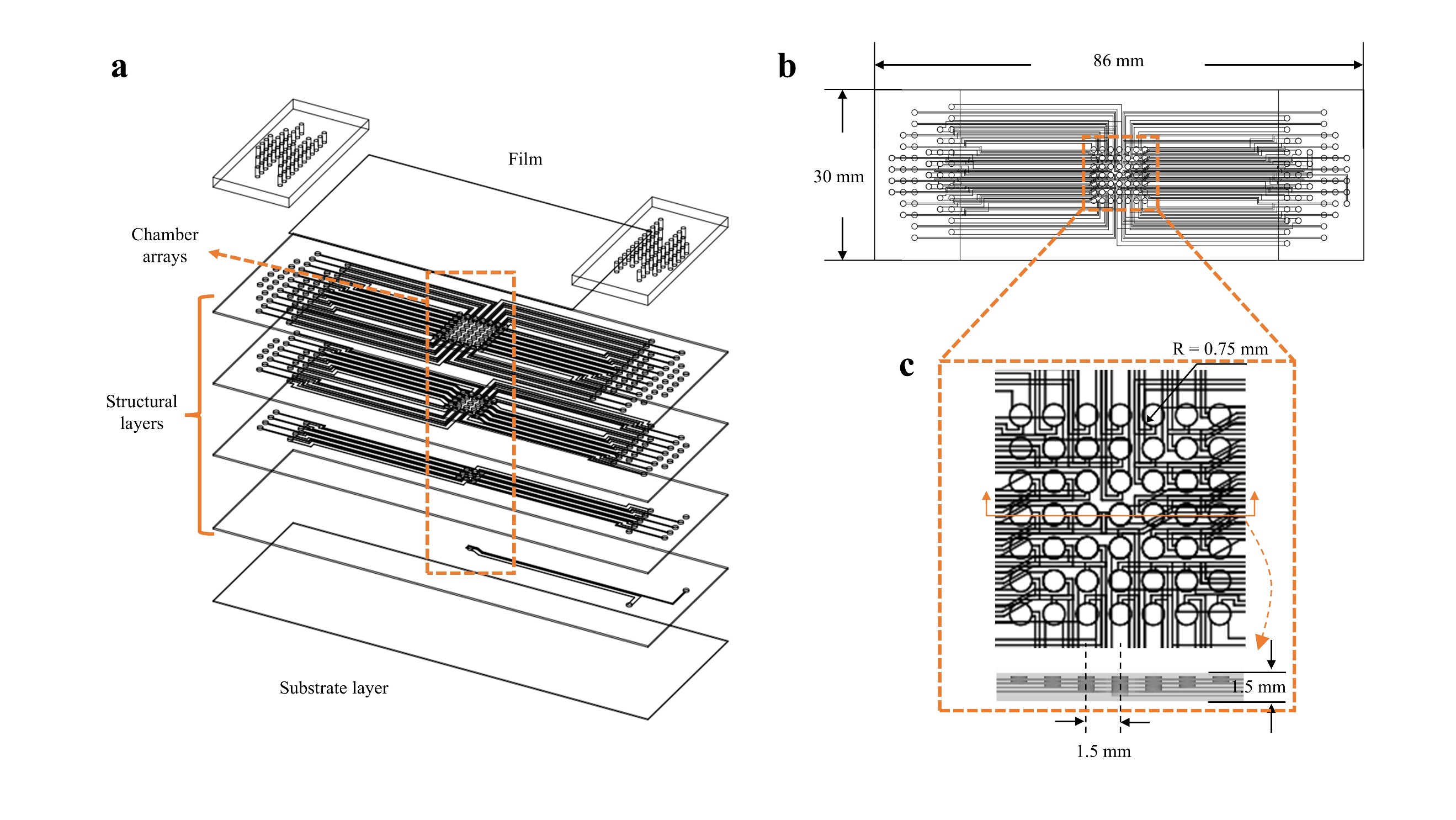


Supplementary Fig. 6 | Structural design of the high-resolution morphable haptic interface. a, Exploded-view schematic of the multilayer structure, comprising chamber arrays, structural layers, and a substrate layer. The core structure integrates chambers of varying sizes with a microfluidic channel system, enabling independent pneumatic actuation. b, Top-down view of the microfluidic channel layout, showing an 86 mm × 30 mm device footprint. The interconnected channel network is designed for efficient pressure distribution across the chamber arrays. c, Magnified view of the core microfluidic network, highlighting an inverted pyramid chamber array with a chamber radius of 0.75 mm and a misaligned channel system. This design enhances spatial resolution and minimizes unintended pressure crosstalk.


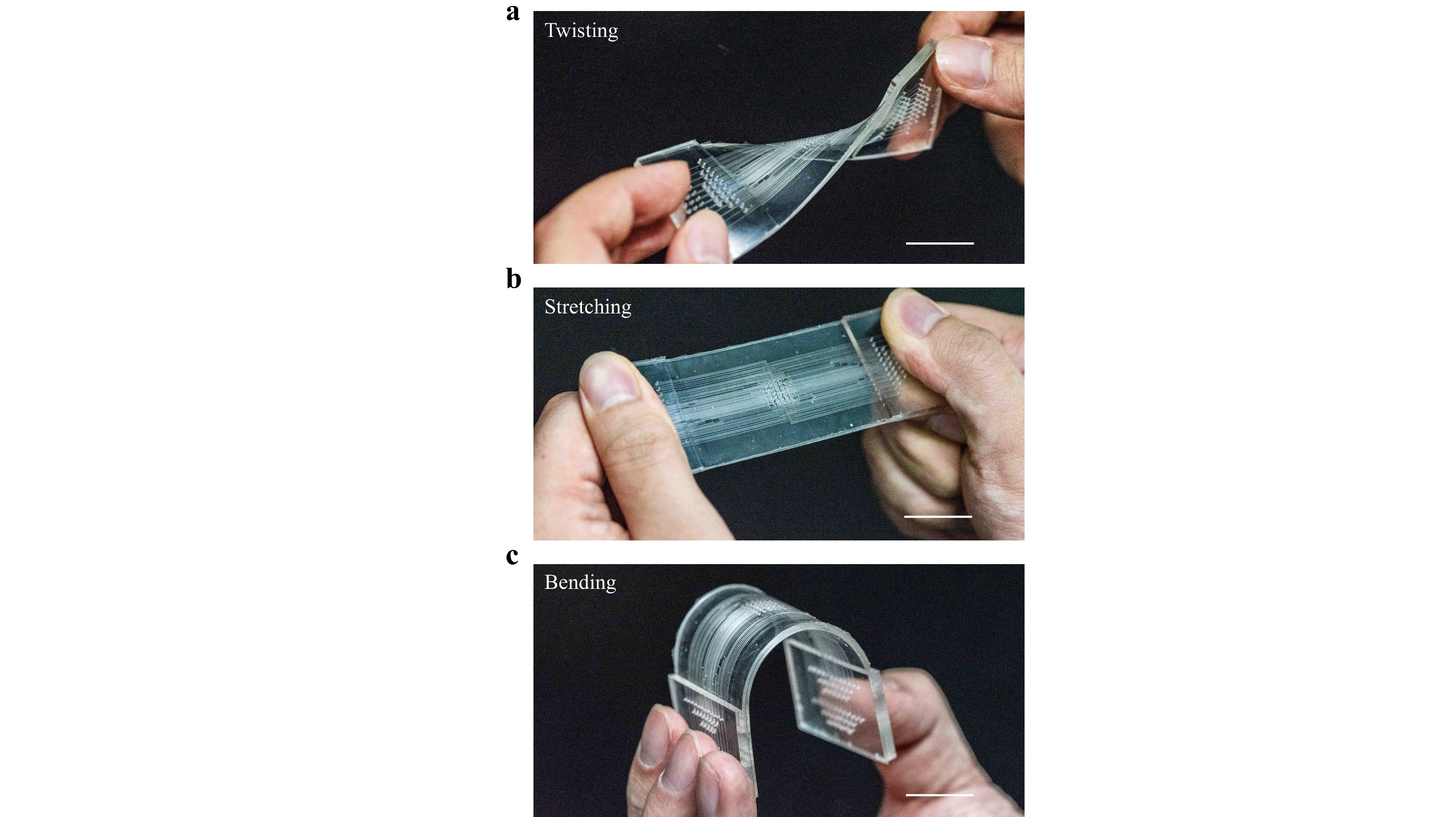


Supplementary Fig. 7 | Optical images of the haptic interface under a, twisting, b, stretching, and c, bending. Scale bar, 2 cm.


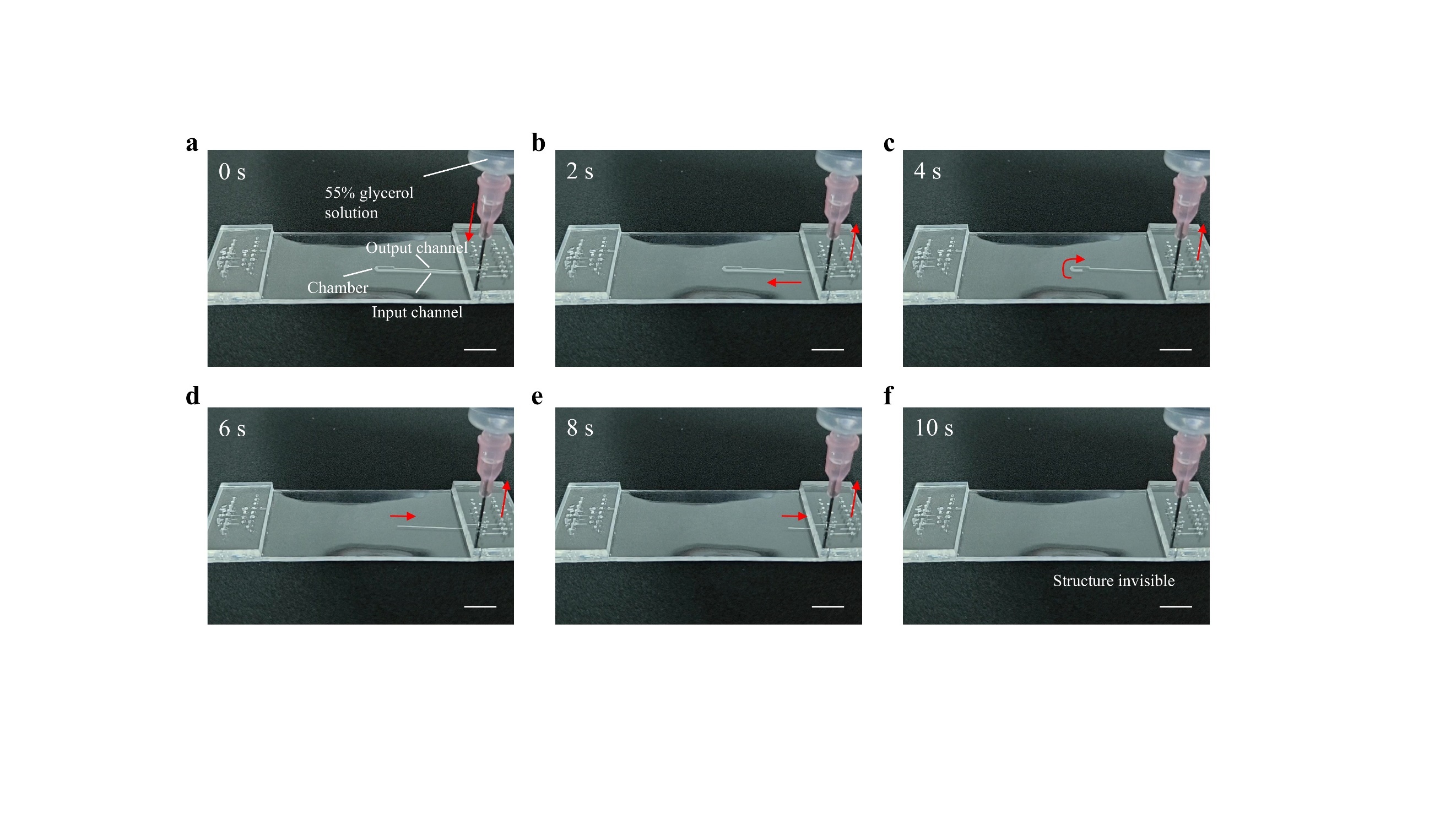


Supplementary Fig. 8 | Optical transparency modulation via microfluidic infusion. a, Initial state of the haptic interface, showing a 7×7 microfluidic chamber array with input and output channels. The first 48 chambers have already been infused with a 55 wt% glycerol solution, leaving only the highlighted chamber for injection, which is the only visible structure at this stage. (b–e) Sequential infusion of the glycerol solution over 2–8 s, progressively filling the chamber and replacing the air within the microfluidic network. The fluid front advances through the input channel, displacing the initial air content. f, At 10 s, the chamber is fully infused, rendering the internal microstructures optically invisible due to the matched refractive index between the glycerol solution and the surrounding material. Scale bars, 5 mm.


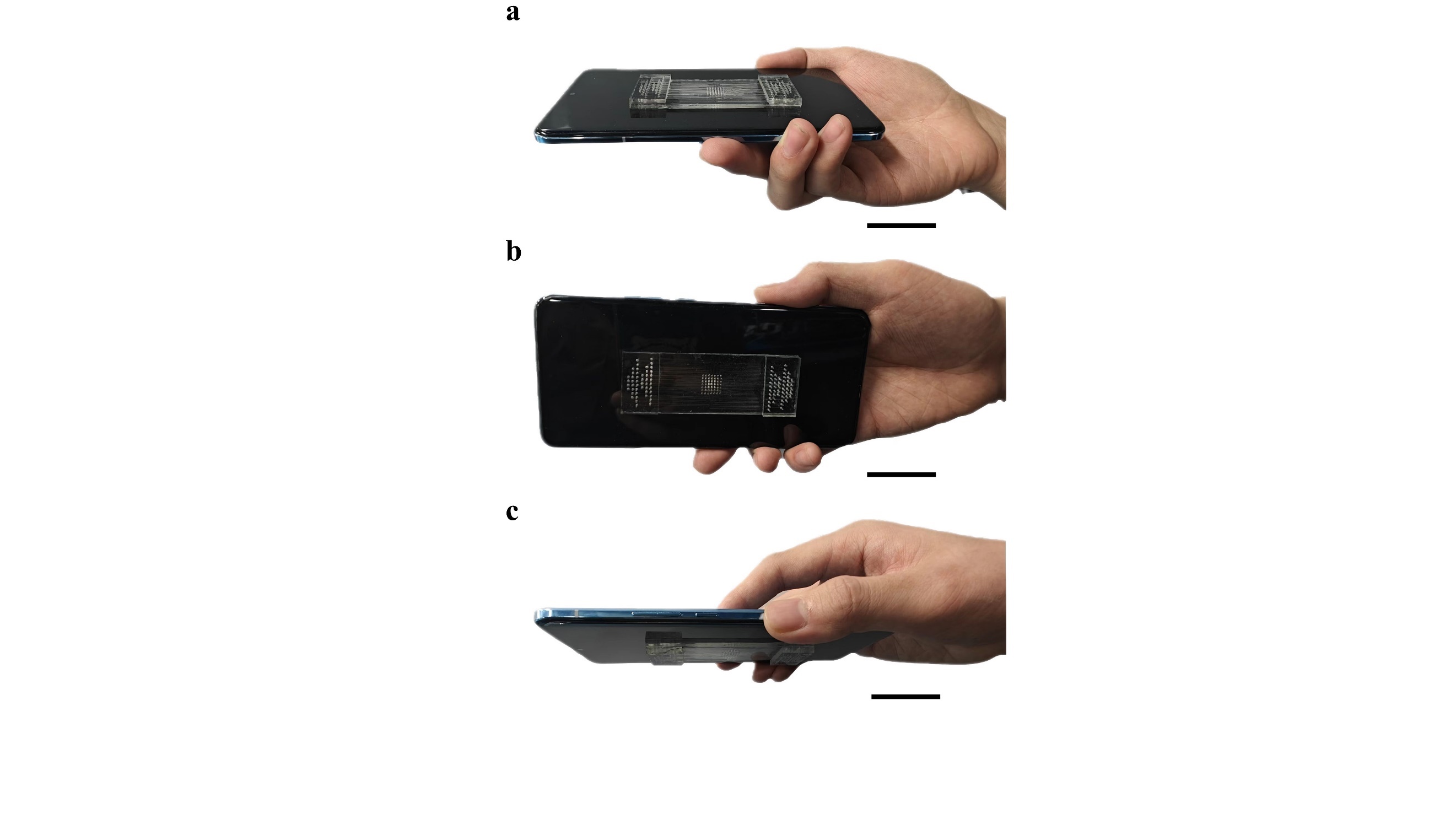


Supplementary Fig. 9 | Optical image of the haptic interface naturally attached to the smartphone touchscreen. a, Horizontal placement. b, Vertical placement. c, Inverted placement. Scale bar, 3 mm.


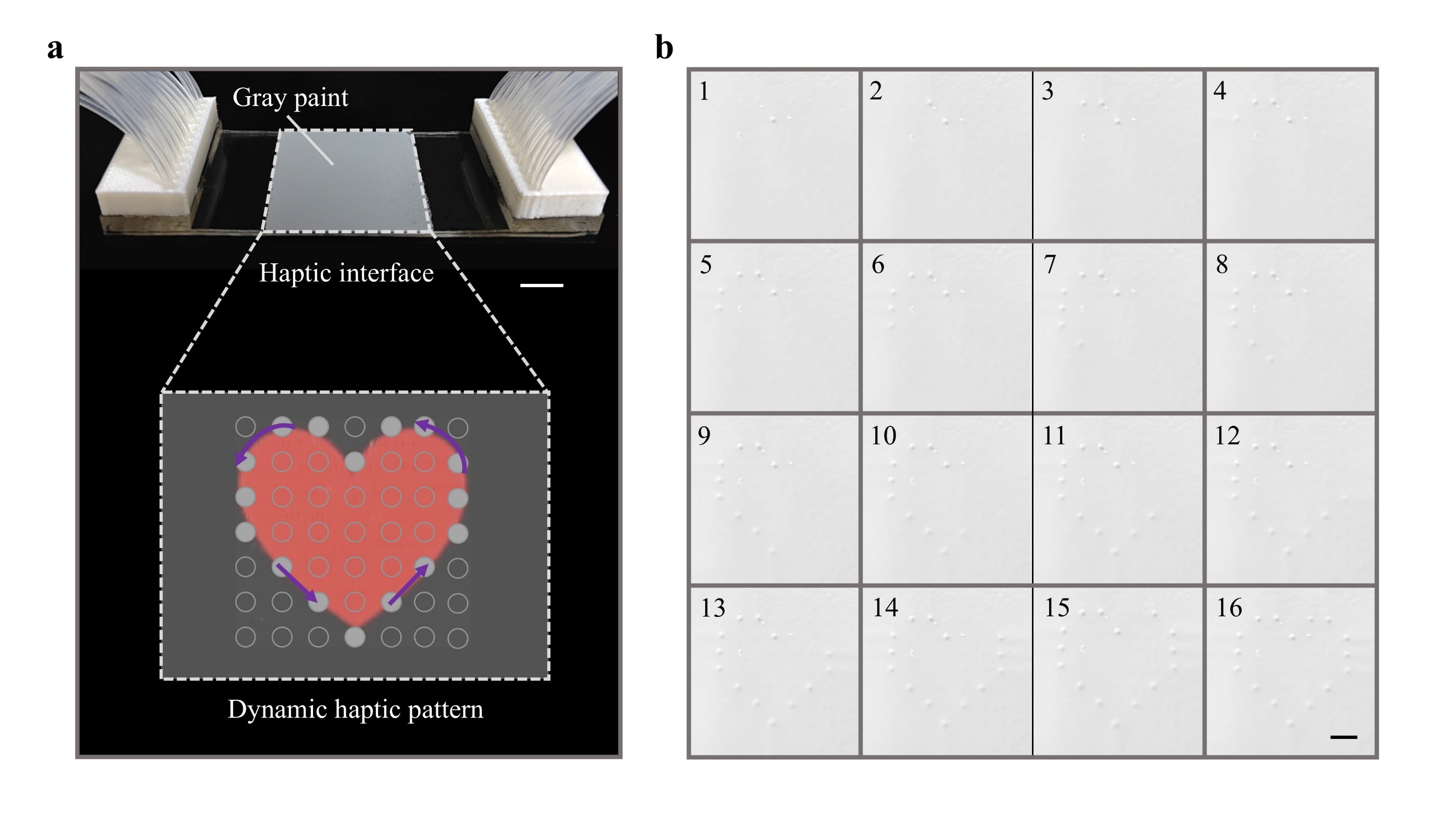


Supplementary Fig. 10 | Dynamic haptic animation of a heart shape through a haptic interface. a, A haptic device integrated with a 7×7 microfluidic actuator array, designed for high-resolution tactile feedback. The image shows the haptic interface after liquid injection, with a gray coating applied to enhance the visibility of the otherwise transparent domes (taxels). A heart-shaped pattern is positioned beneath the interface, demonstrating the system’s ability to generate precise, programmable tactile structures. Scale bar, 2 mm. b, The raised taxels reconstruct a heart-shaped outline that closely aligns with the underlying image, showcasing the system’s capacity for spatially accurate haptic rendering. The driving interval between adjacent taxels is 0.2 s, allowing for sequential activation and dynamic animation effects. Scale bar, 2 mm.


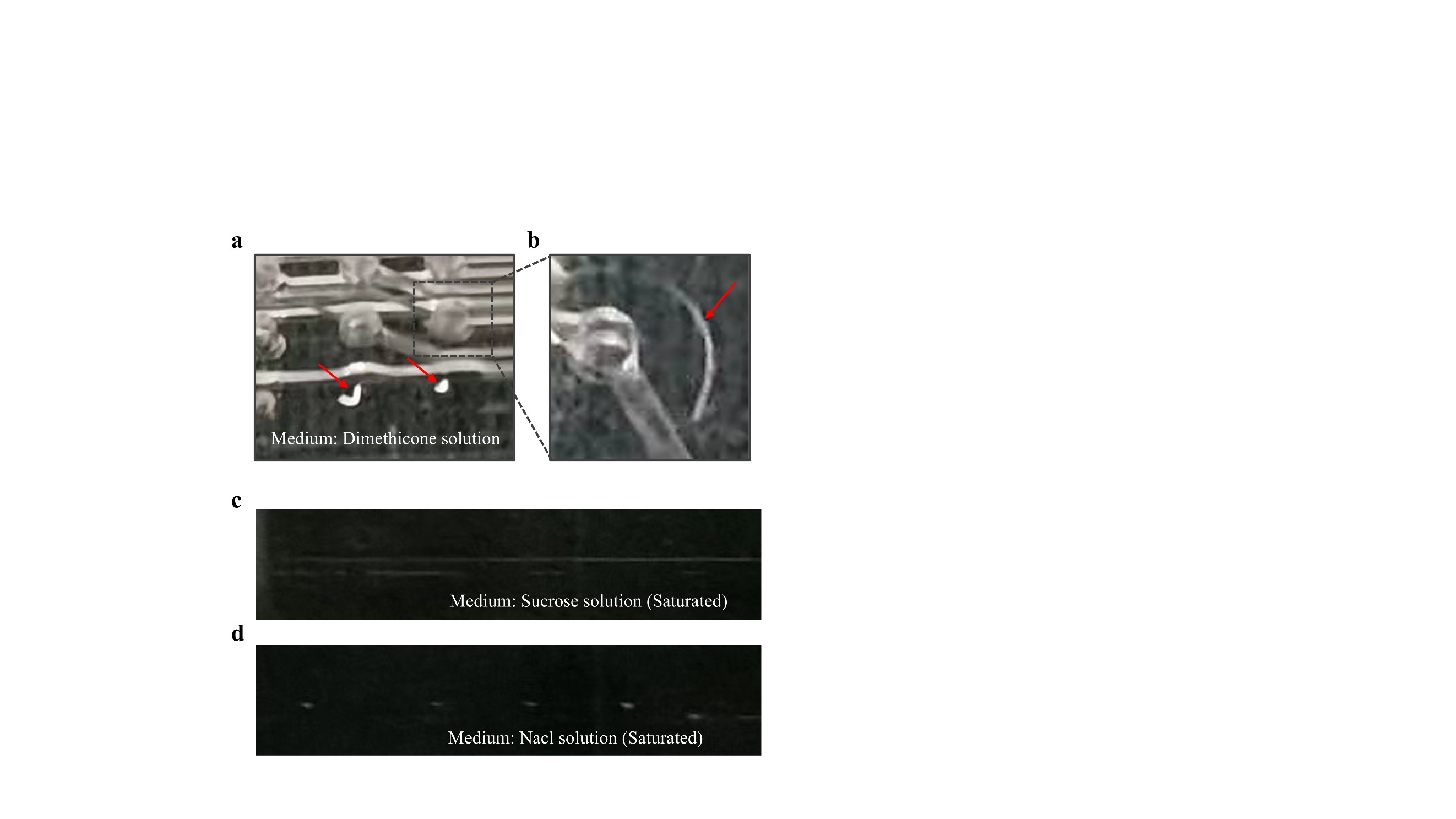


Supplementary Fig. 11 | Failure modes of the other three fluid media. Dimethicone solution causes swelling to occur in both the a, film and b, bonding regions. c, The channel becomes visible due to the lower refractive index of the sucrose solution compared to PDMS (n ≈ 1.41). d, NaCl crystallization precipitating at room temperature.


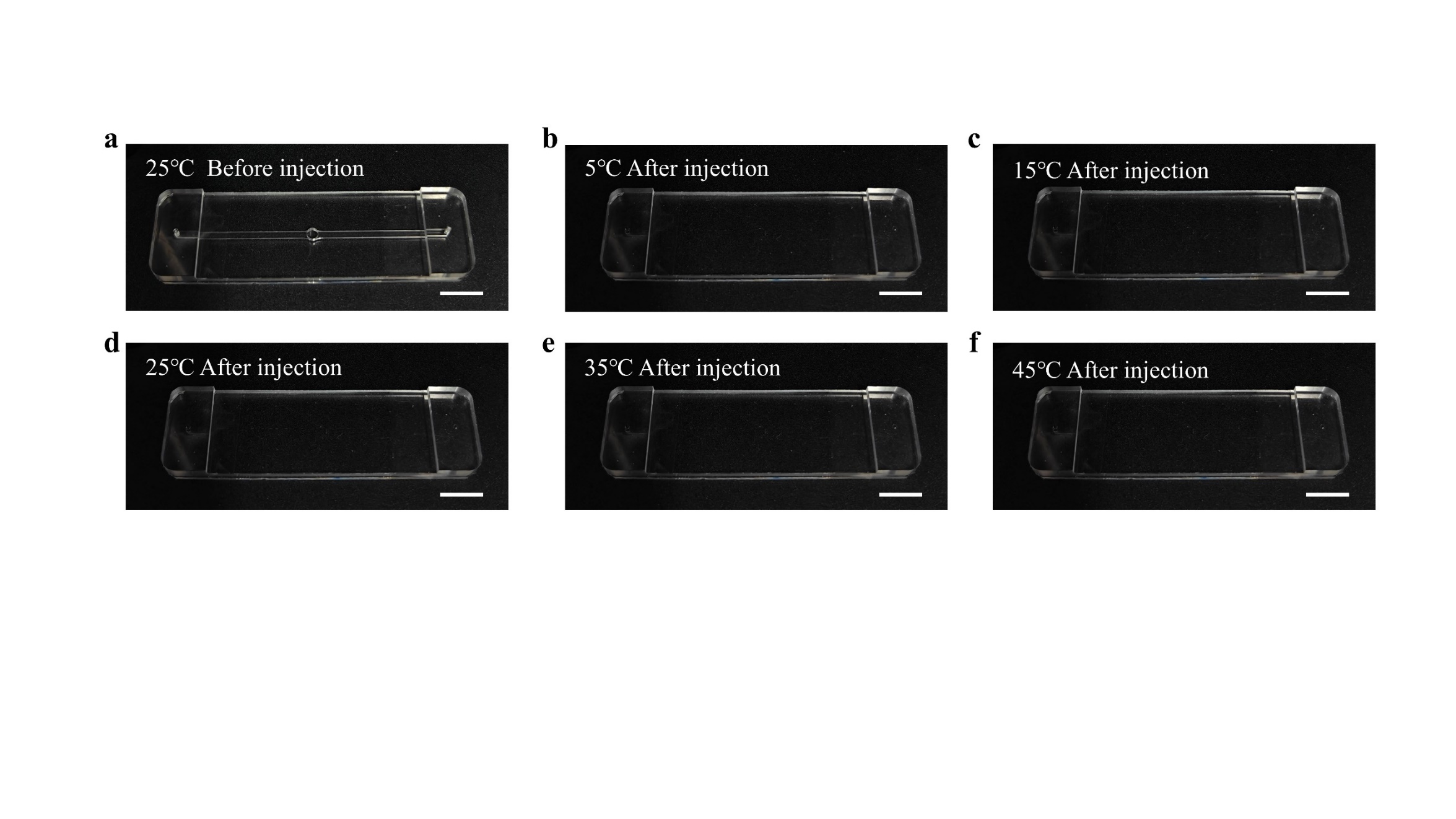


Supplementary Fig. 12 | Optical images of the actuator at different temperatures. The ambient temperature is set between 5°C and 45°C (a-f) and the injected liquid is a 55% mass fraction glycerol solution. Scale bar, 8 mm.


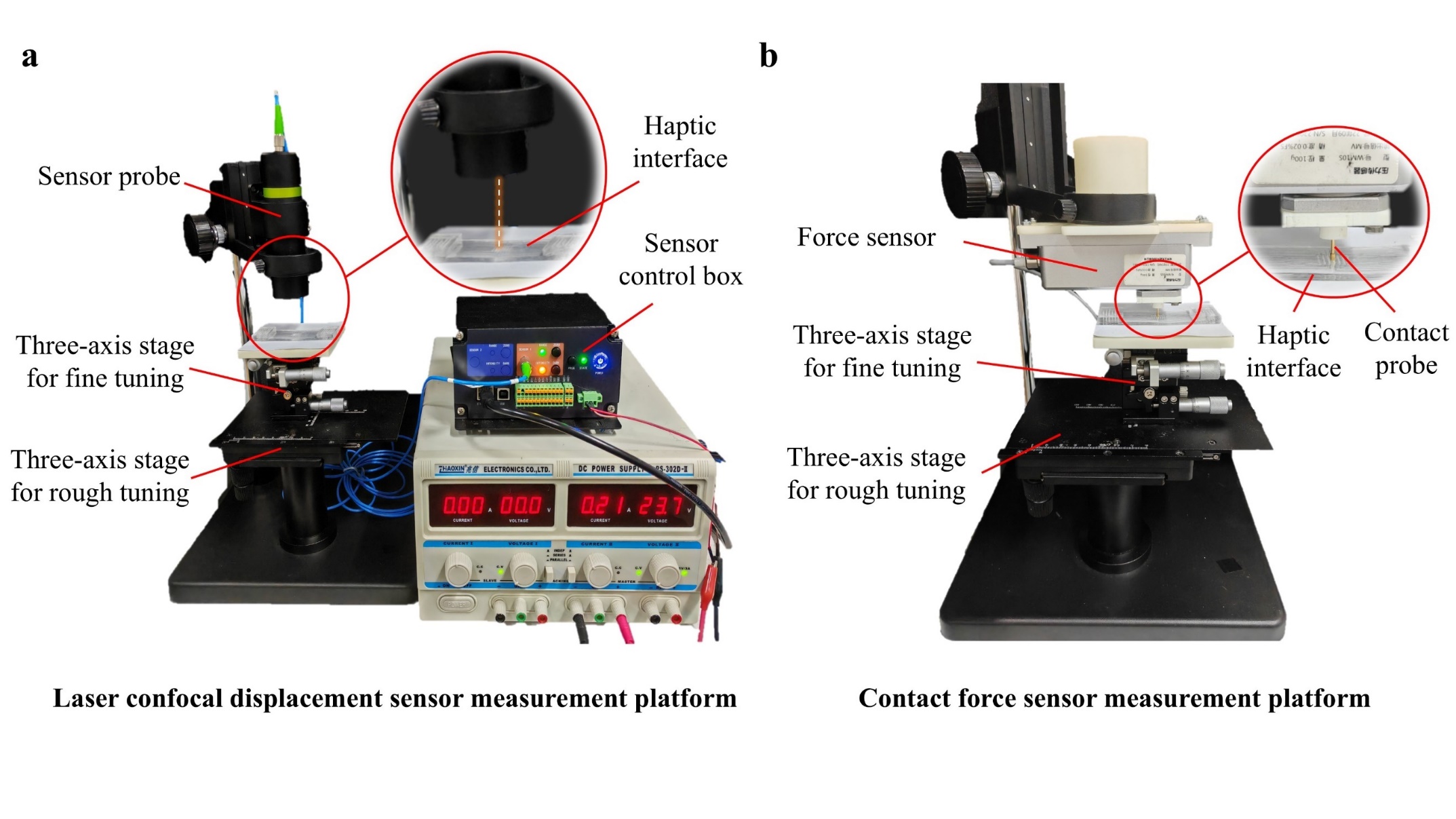


Supplementary Fig. 13 | Experimental setups for testing the output displacement and force of microfluidic actuators. a, A spectral confocal displacement sensor (LTC 4000F, Hongchuan Technology) and b, a parallel cantilever beam force sensor (AR-WM10S, Arizon Technology) are above the haptic device to record the output displacement and force of the actuator, maximum sampling frequency: 1000Hz.


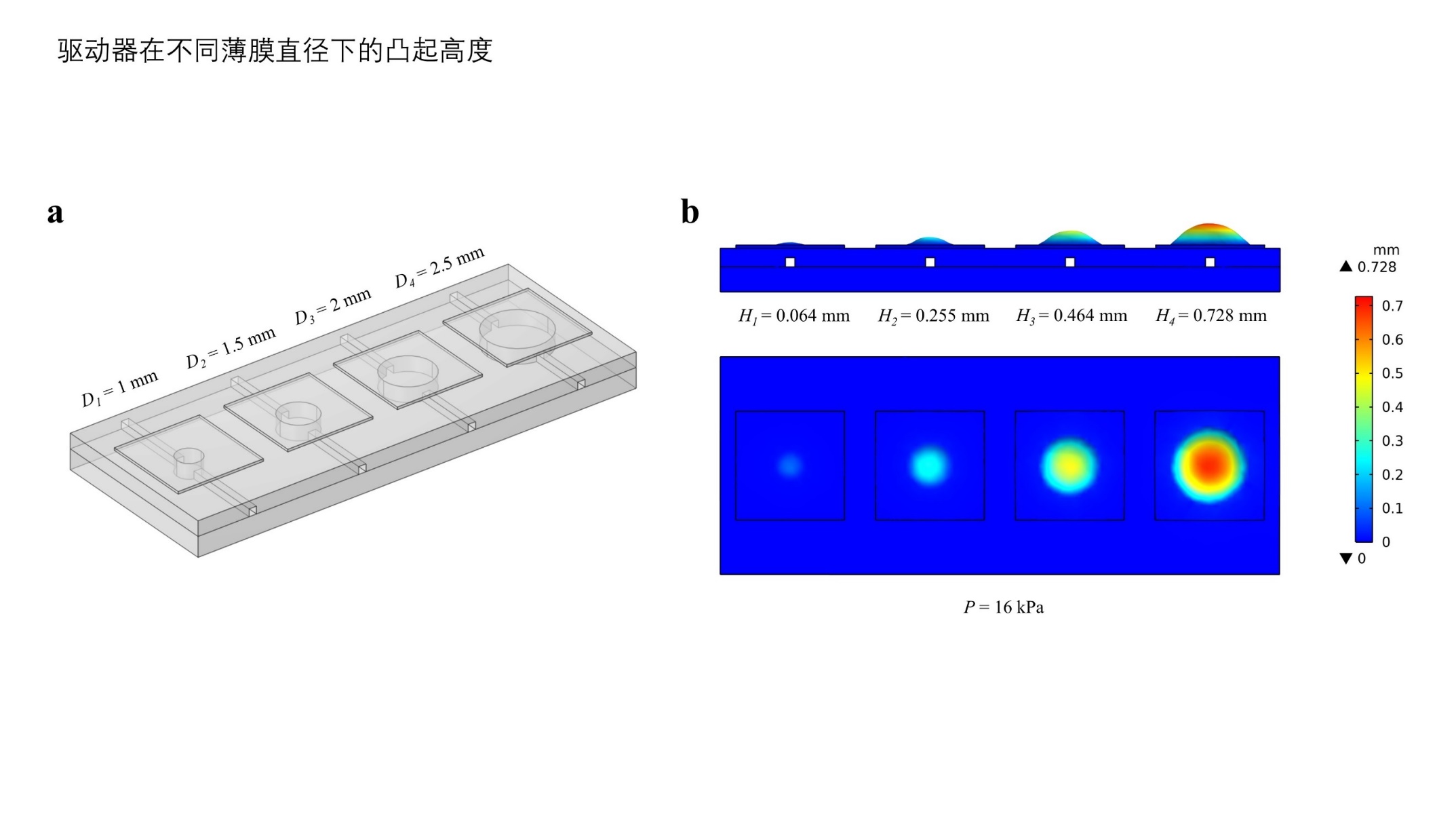


Supplementary Fig. 14 | Finite element simulation of actuators with different chamber diameters. a, The schematic diagram of the simulation model where the diameter of chambers (*D*) is set to 1 mm, 1.5 mm, 2mm, and 2.5 mm, respectively, the parameters of the membrane above the chamber include a thickness of 100 μm and a modulus of 1.0 MPa. b, Schematic diagram of the simulation results of the actuator displacement when the input pressure (*P*) is set to 16 kPa, in which *H_1_* = 0.064 mm, *H_2_* = 0.255 mm, *H_3_* = 0.464 mm, and *H_4_* = 0.728 mm.


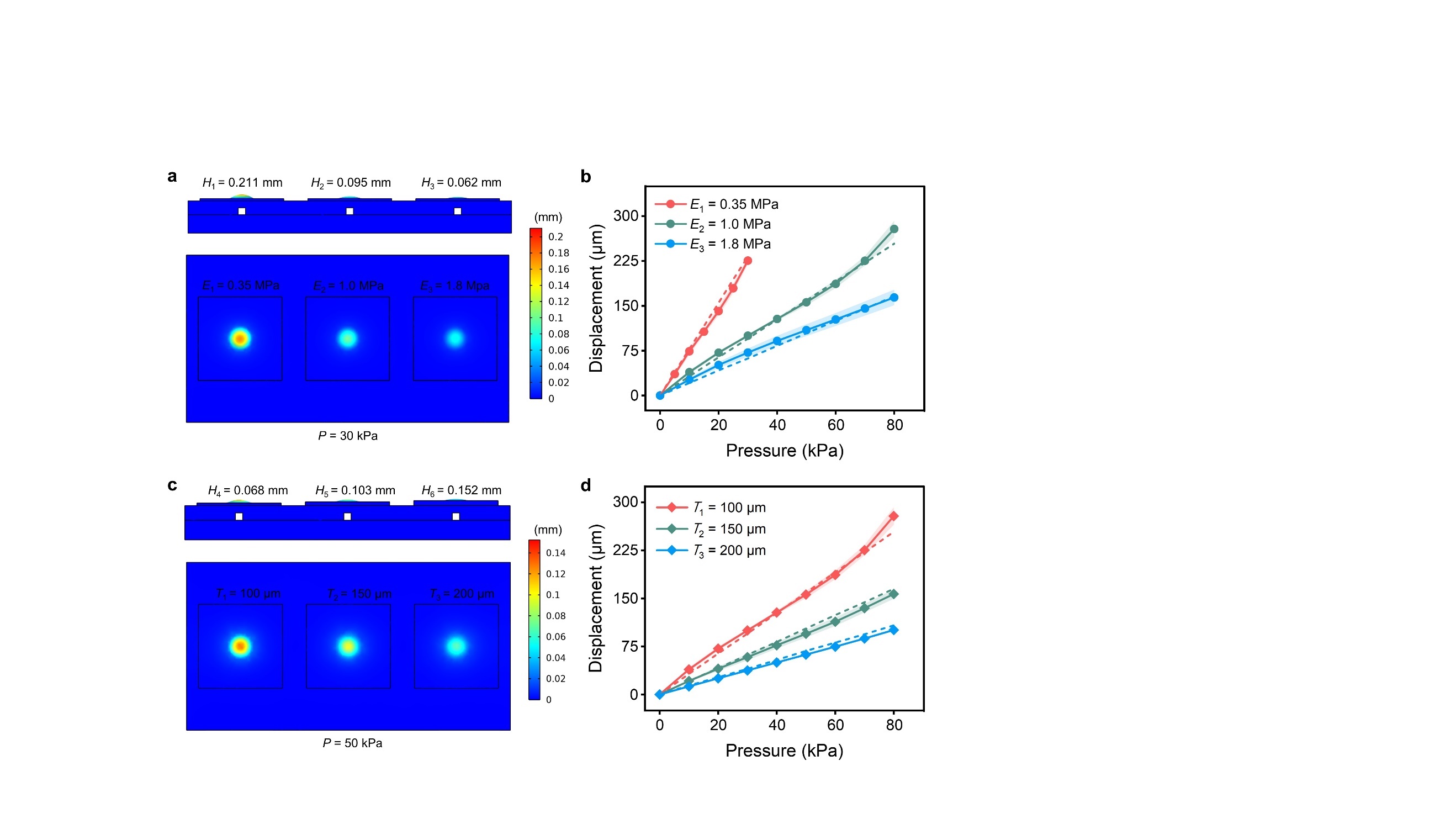


Supplementary Fig. 15 | Output displacement of the actuator with different film thickness modulus. a, Schematic diagram of the simulation results of the actuator displacement when the input pressure (*P*) is set to 50 kPa, in which *H_1_* = 0.068 mm, *H_2_* = 0.103 mm, *H_3_* = 0.152 mm. The thickness of films (*T*) is set to 100 μm, 150 μm, and 200 μm and a modulus of 1.0 MPa, respectively, and the diameter of the chamber is set to 1 mm. b, The simulation results of the actuator displacement under different input pressure (*P*). Note: Data corresponding to E = 0.35 MPa are not presented due to membrane rupture observed during testing. Points, mean; error bars, s.d.; *n* = 5 independent samples. Solid lines, experimental data; dotted lines, simulation data. c, Schematic diagram of the simulation results of the actuator displacement when the input pressure (*P*) is set to 30 kPa, in which *H_1_* = 0.211 mm, *H_2_* = 0.095 mm, *H_3_* = 0.062 mm. The modulus of films (*E*) is set to 0.35 MPa, 1.0 MPa, 1.8 MPa, and a thickness of 100μm, respectively, and the diameter of the chamber is set to 1 mm. d, Average output displacement of the actuator with different film modulus under different input pressures. For films with a lower elastic modulus (*E_1_* = 0.35 MPa), there is a risk of rupture when the input pressure exceeds 30 kPa. Points, mean; error bars, s.d.; *n* = 5 independent samples. Solid lines, experimental data; dotted lines, simulation data.


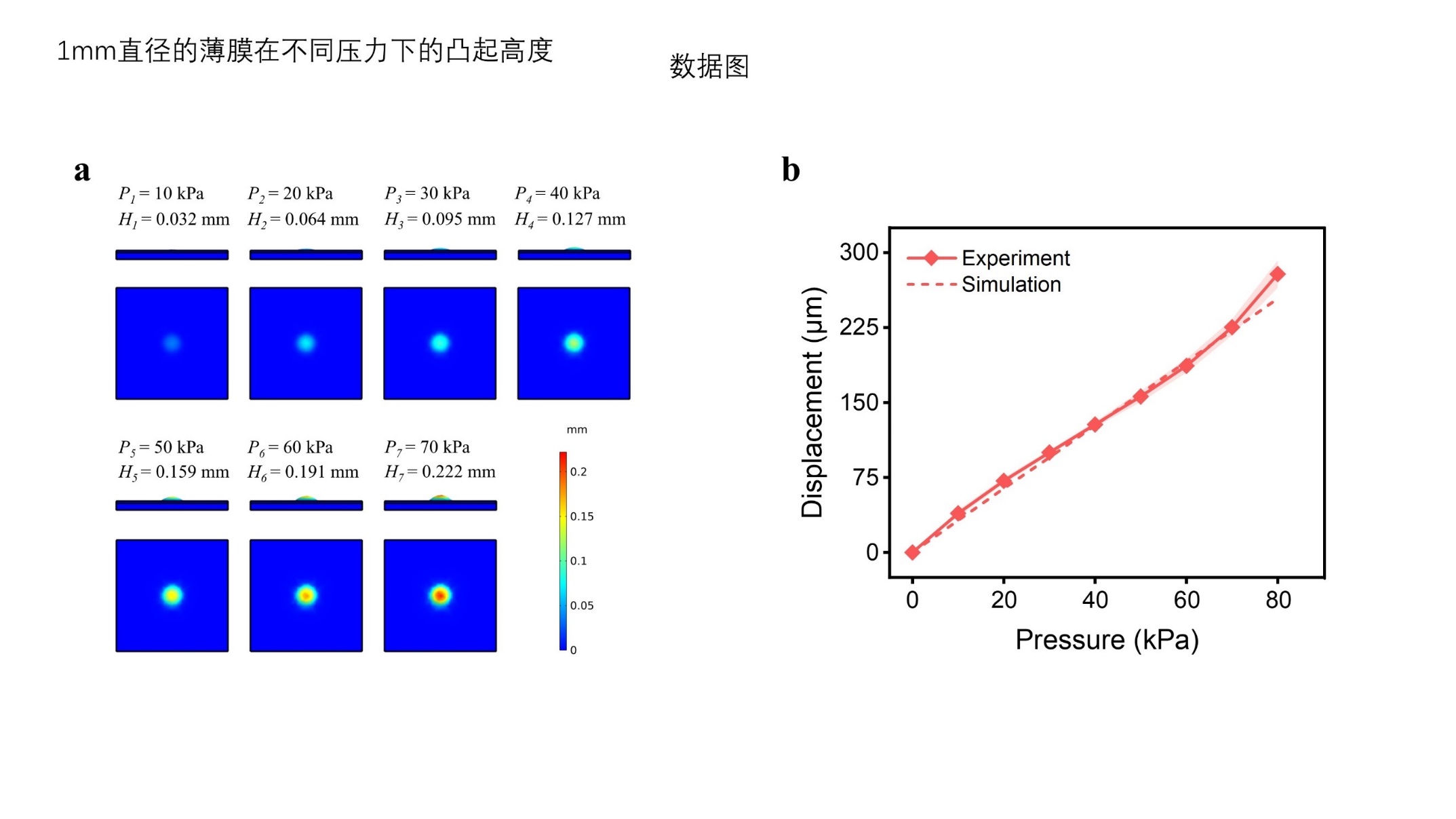


Supplementary Fig. 16 | Output displacement of the actuator as a function of the input pressure (data points). a, Schematic diagram of the simulation results of the actuator displacement under different input pressures. The modulus of films is set to 1.0 MPa and a thickness of 100μm, respectively, and the diameter of the chamber is set to 1 mm. b, Average output displacement of the actuator under different input pressures. Points, mean; error bars, s.d.; *n* = 5 independent samples. Solid lines, experimental data; dotted lines, simulation data.


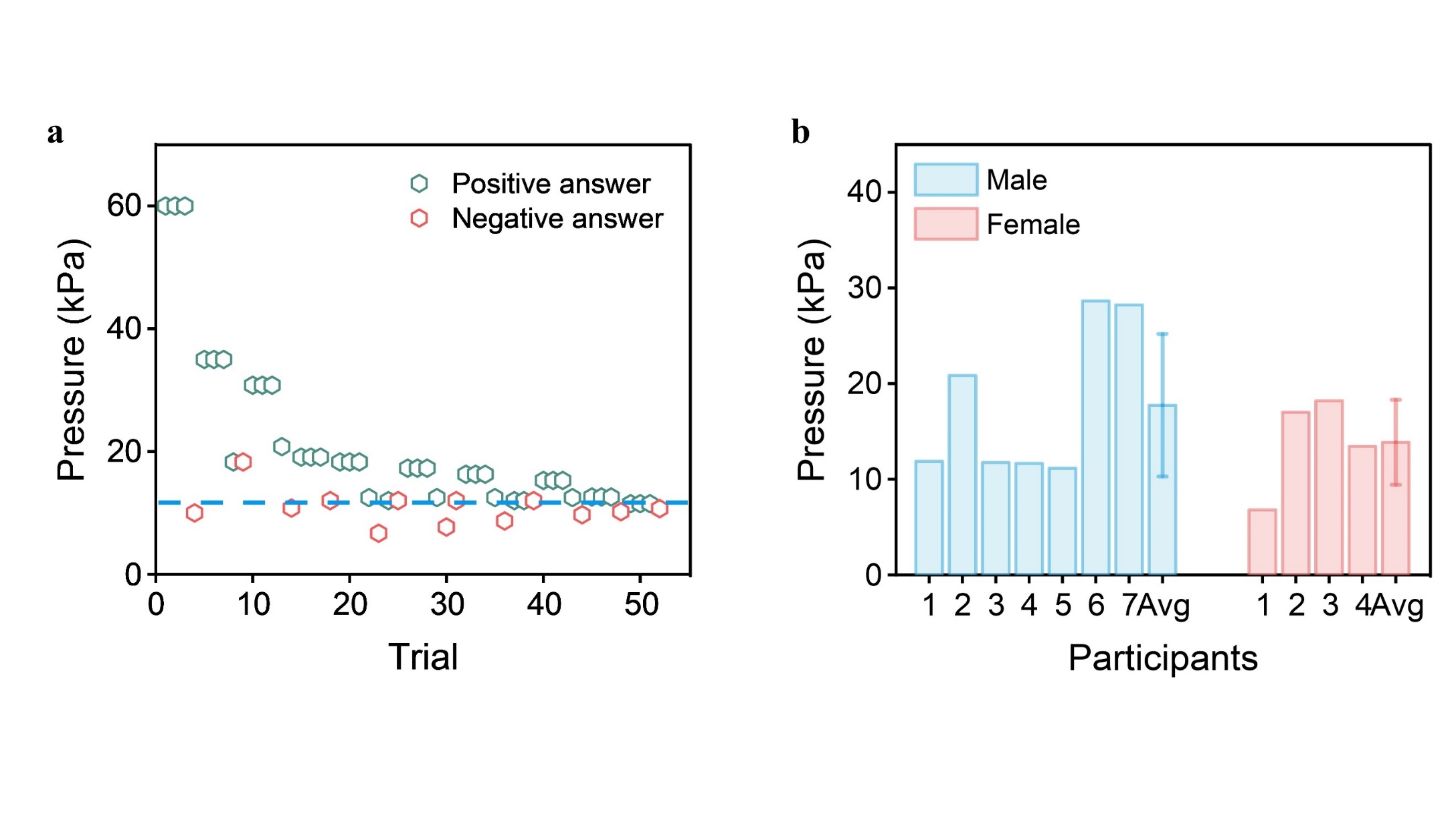


Supplementary Fig. 17 | User study on absolute thresholds of input pressure. a, Experimental result of the first participant, larger step sizes were used in the initial reversals to quickly approach the estimated threshold level. After more than 8 reversals, smaller step sizes were used to improve the accuracy of threshold estimation until pressure values converged (*T*_i_ ≤ 1 kPa) or reached 50 trials. If convergence was not achieved after 50 trials, the number of trials was increased accordingly until convergence was reached. b, Absolute threshold of input pressure for a total of 11 participants (7 males, 4 females). Bar height, mean; error bars, s.d.; *n* = 50 independent samples.


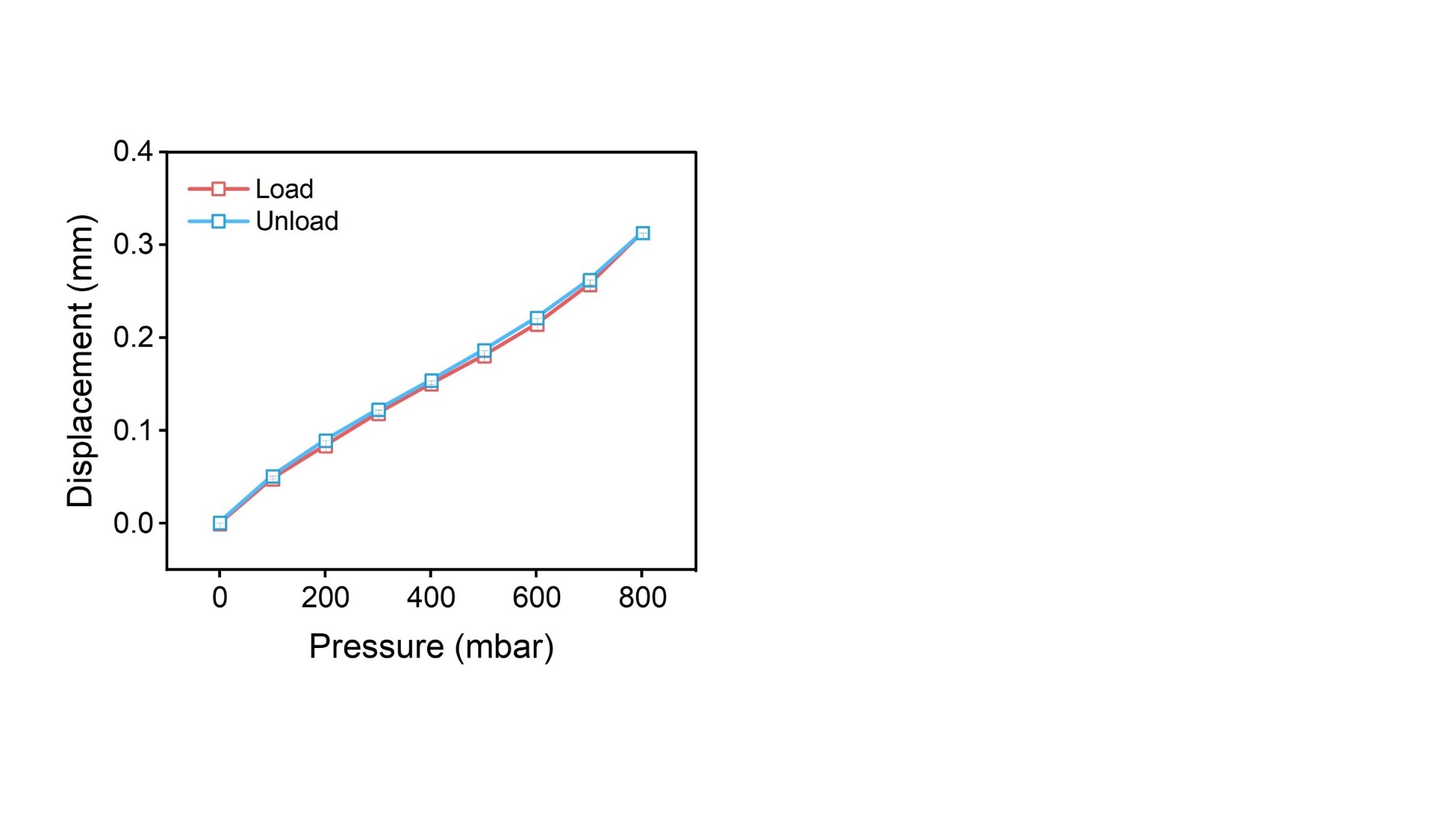


Supplementary Fig. 18 | Hysteresis performance of the actuator. Supplementary Figure shows the relationship between input pressure and displacement of the actuator. A step pressure signal is input to the actuator through the pressure control system. The pressure range is 0-80 kPa and the pressure step is 10 kPa. We record the loading process (red) and the unloading process (blue) of the actuator through a spectral confocal displacement sensor (LTC 4000F, Hongchuan Technology). The actuator exhibits reliable cycling performance with minimal hysteresis and no residual strain after unloading. Points, mean; error bars, s.d.; *n* = 5 independent samples.


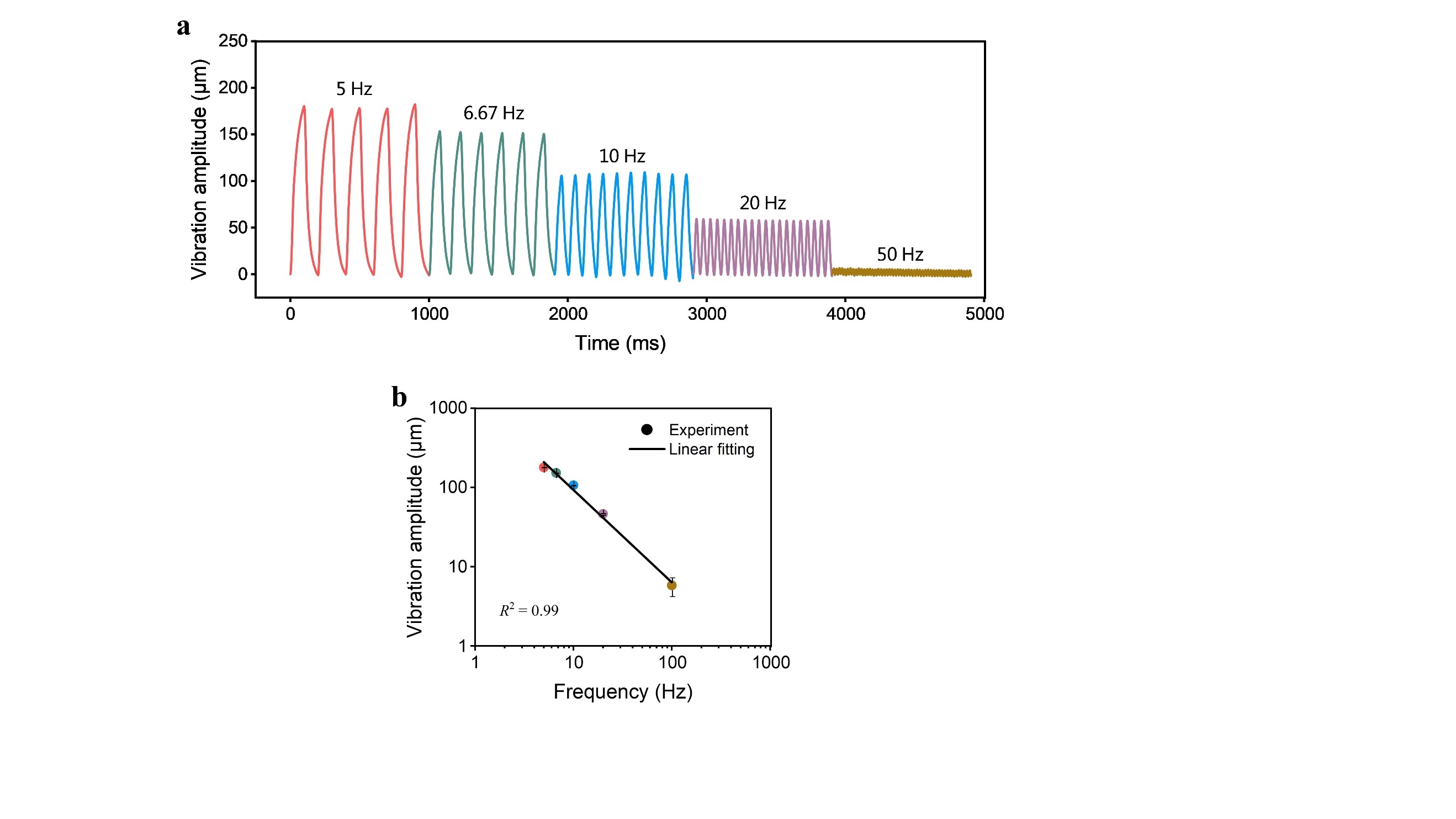


Supplementary Fig. 19 | Dynamic performance of the actuator at different actuation frequencies. a, Time domain responses of amplitude at different frequencies (i.e., 5Hz, 6.67Hz, 10Hz, 20Hz, and 50Hz). b, The linear relationship between the actuation frequency and the vibration amplitude of the actuator. Points, mean; error bars, s.d.; *n* = 5 independent samples.


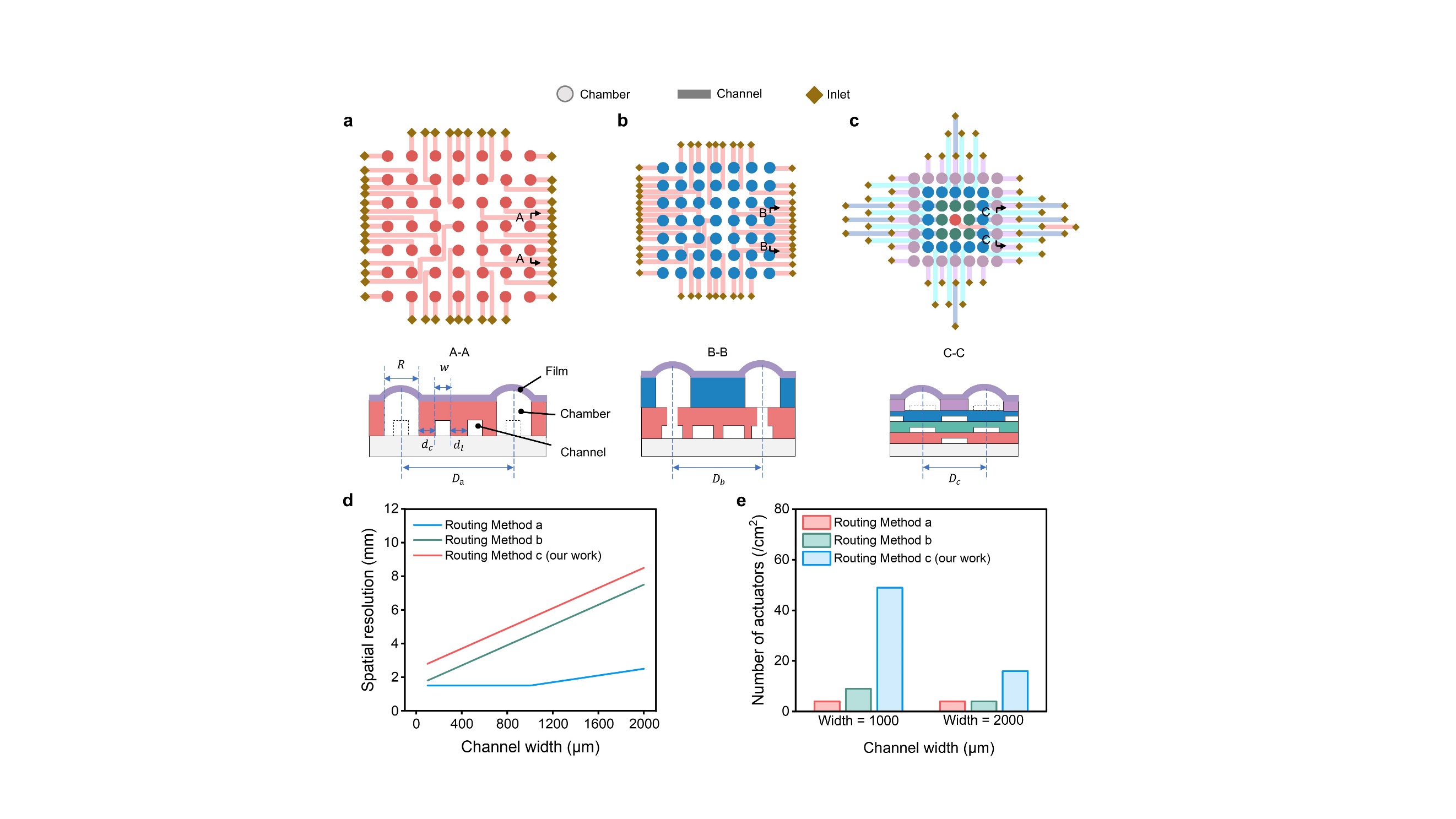


Supplementary Fig. 20 | Comparison of different chamber-channel distribution architectures and their effects on spatial resolution and actuator density. a, A single-layer design in which chambers and channels are co-planar, leading to a low-density configuration. b, A dual-layer structure that separates chambers and channels into different layers, achieving a medium-density arrangement. c, A multilayer stacked channel network with a hierarchical routing scheme, enabling a high-density configuration (our work). d, Spatial resolution as a function of channel width for the three routing methods, showing that our multilayer approach achieves the highest resolution. e, Actuator density per square centimeter for different routing methods under two channel width conditions (1000 µm and 2000 µm), demonstrating the superior scalability of our approach. Parameters set: *R* = 1 mm, *d*_c_ = *d*_l_ =0.5 mm, *N* = 2.


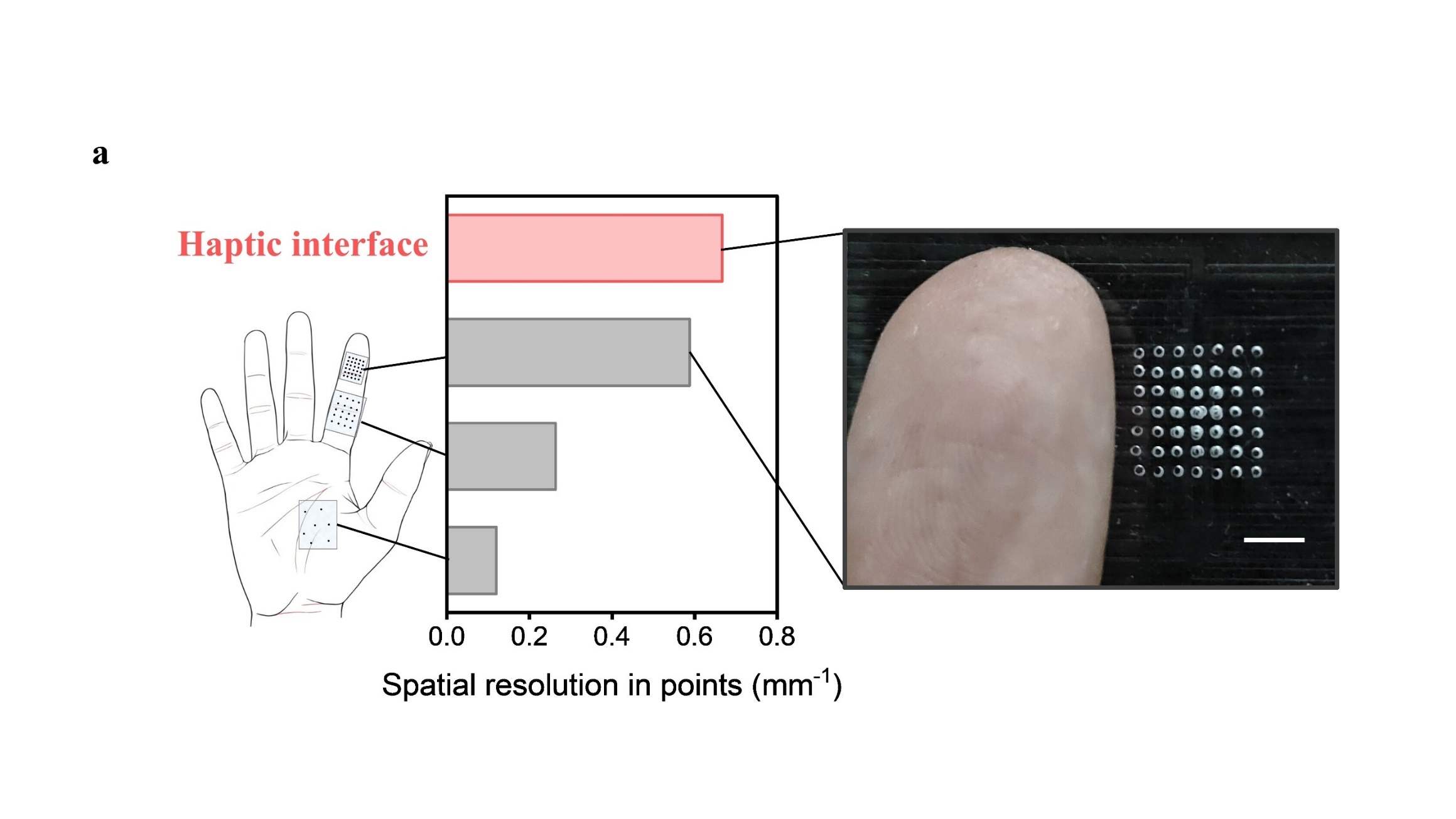


Supplementary Fig. 21 | Spatial resolution of the haptic interface and different parts of the hand. The right optical image shows a comparison between a fingertip and the 7 × 7 microfluidic chamber array, where the device can generate 49 independent tactile stimuli at different positions within an area the size of the fingertip, offering high-resolution haptic feedback. Scale bar, 5mm.


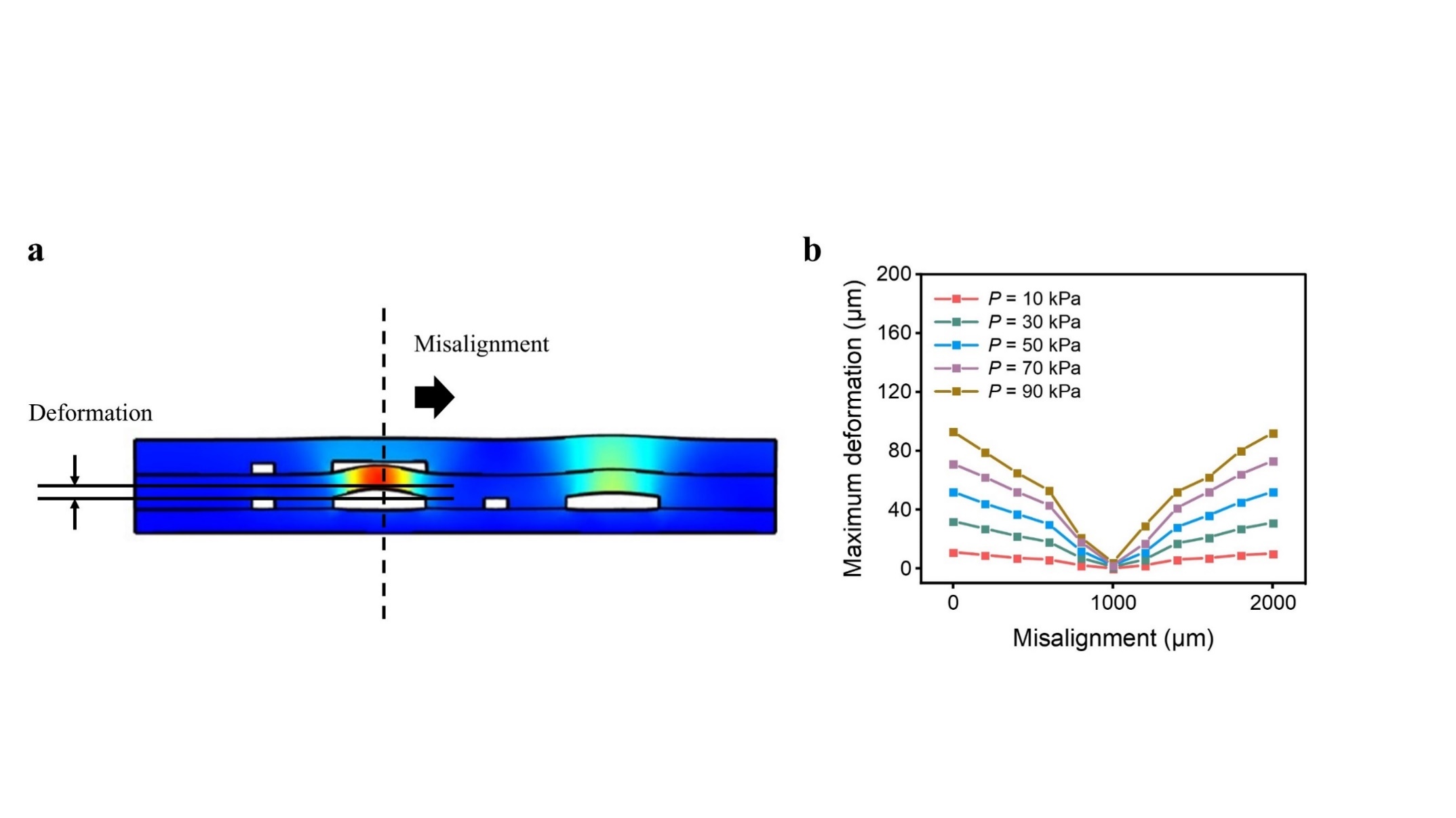


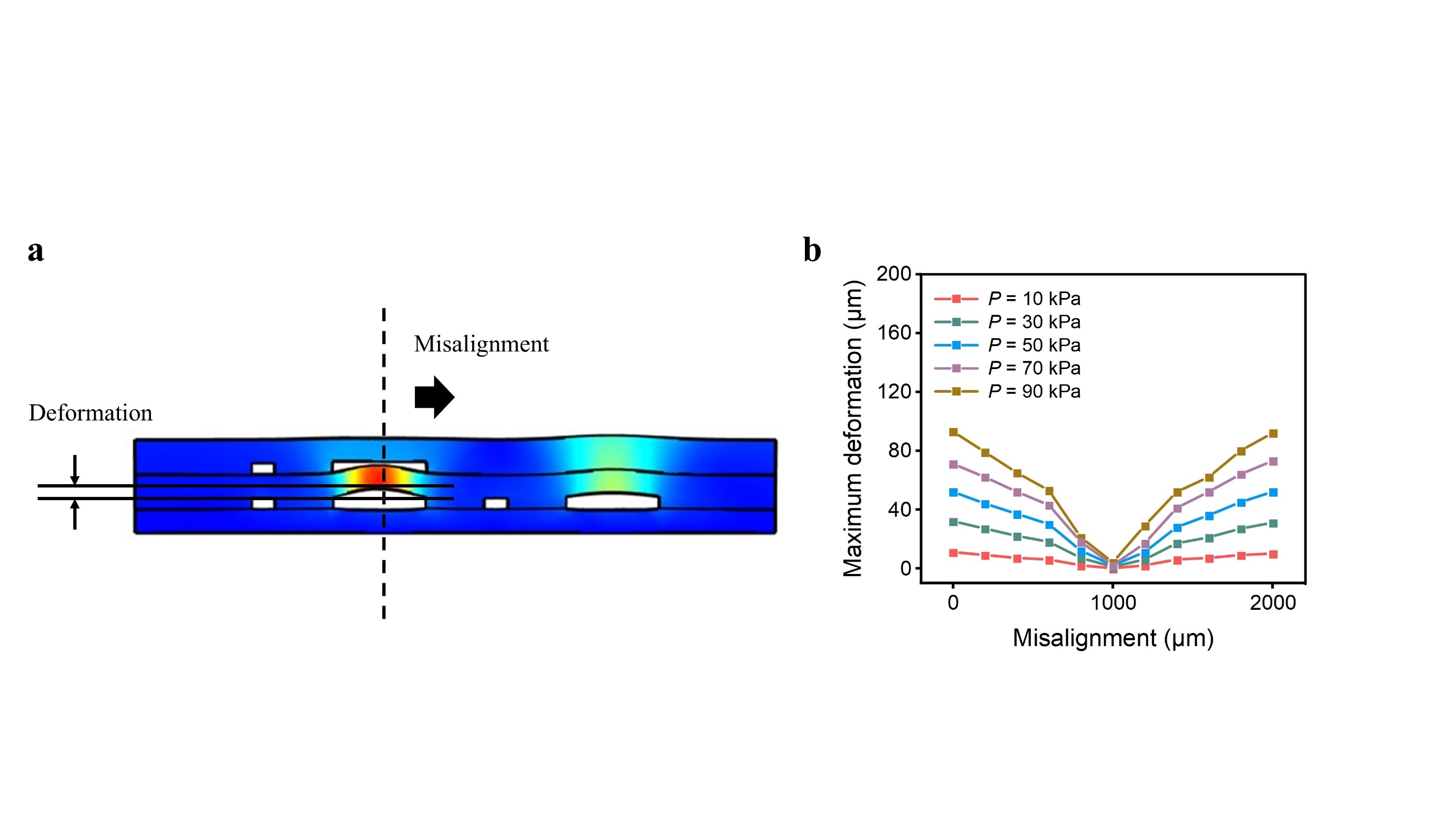


Supplementary Fig. 22 | The simulation results of the relationship between misalignment distances and the maximum deformations of the channels. The misalignment of upper channels is set to 0–2000 μm, respectively, the width of the channel is 800 μm and the height of 100 μm. The results show that the misaligned arrangement of channels can effectively reduce the interference of channels between layers, and the optimal value is obtained at 1000 μm.


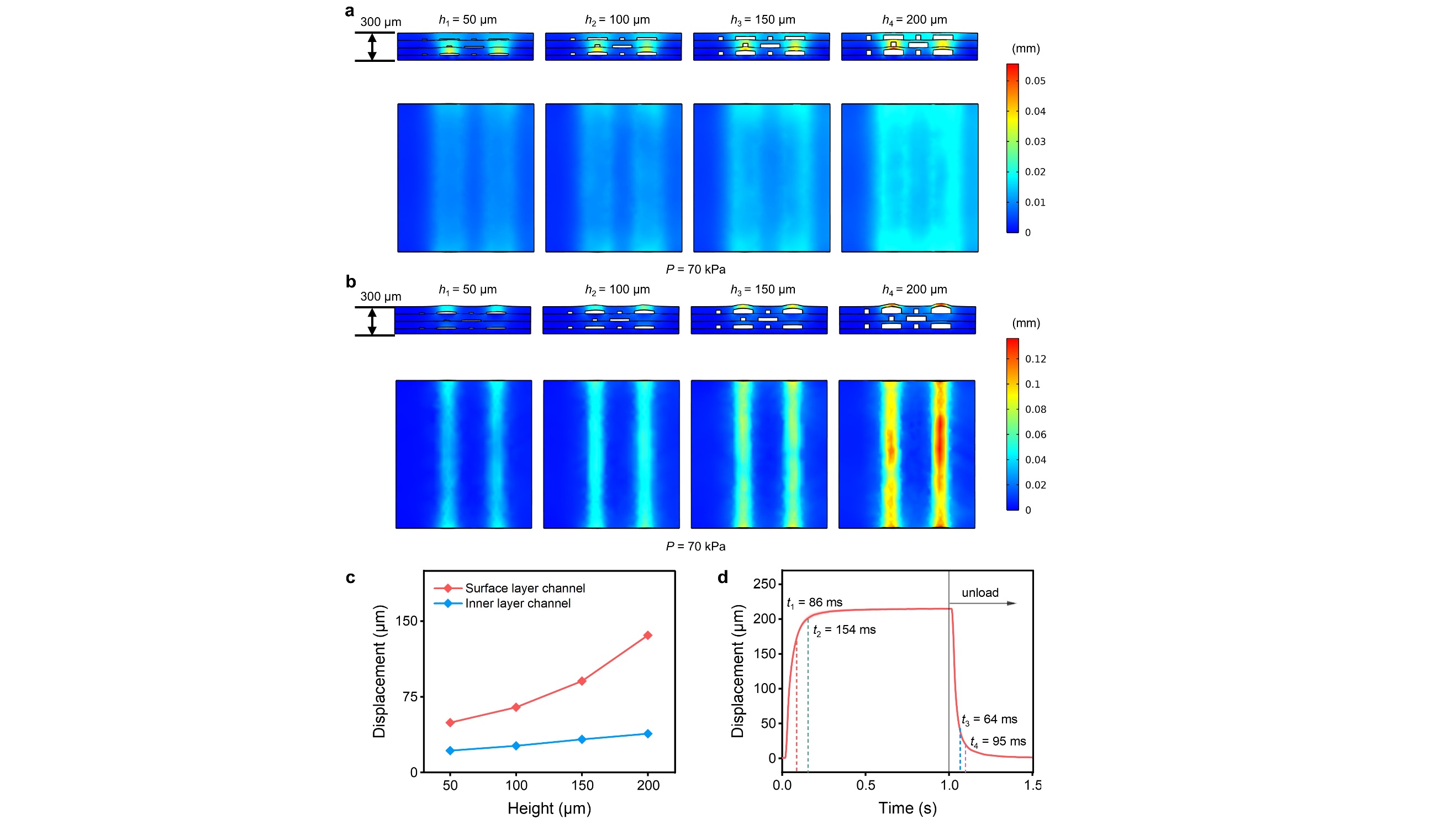


Supplementary Fig. 23 | Influence of channel height on the deformation characteristics of the haptic interface. a, Displacement distribution of the inner-layer channel under a driving pressure of 70 kPa, with varying chamber heights (h_1_ = 50 μm, h_2_ = 100 μm, h_3_ = 150 μm, h_4_ = 200 μm) and a fixed channel width of 800 μm. b, Corresponding displacement distribution for the surface-layer channel under the same driving conditions. The channel width is fixed at 800 μm. c, Simulated relationship between chamber height and maximum displacement, showing a greater deformation response in the surface-layer channel compared to the inner-layer channel. d, Time-dependent displacement of the actuator under a pressure pulse, measured at an optimized channel height of 100 μm and a width of 800 μm. The actuator reaches 80% and 90% of its maximum displacement in t_1_ =86 ms and t_2_ = 154 ms, respectively, during the loading phase. In the unloading phase, the displacement decreases to 20% and 10% of its maximum value in t_3_ = 64 ms and t_4_ = 95 ms, respectively. Lines, mean; error bars, s.d.; n=5 independent samples.


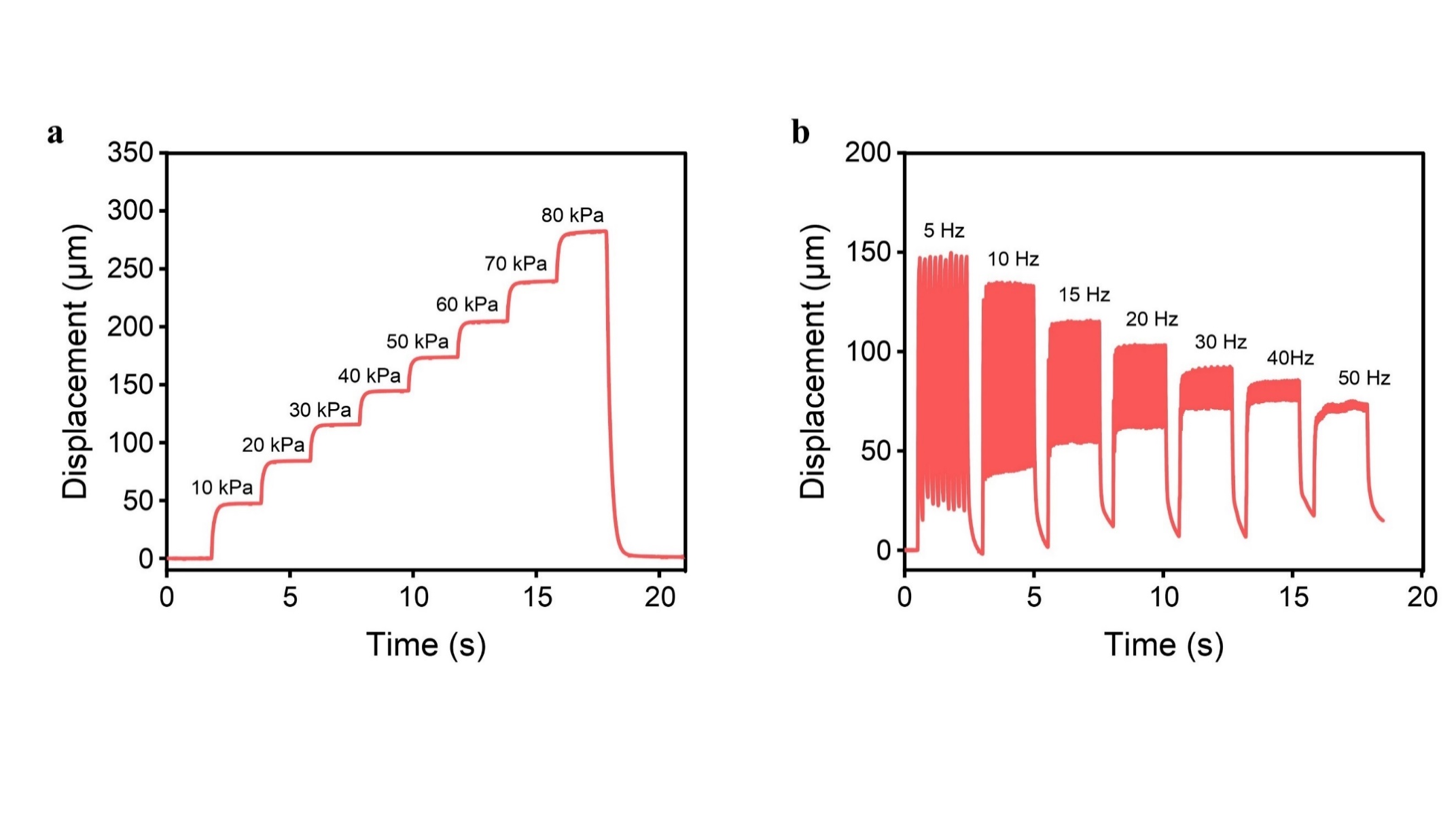


Supplementary Fig. 24 | Dynamic performance of the actuator array with 100 × 800um channel cross-section. a, Output displacement of the actuator array at different pressures. The output displacement of the actuator increases almost linearly with increasing pressure, exceeding 280 μm at an input pressure of 80 kPa. Pressure range: 0 kPa-80 kPa; step size 10 kPa; time interval: 2 s. b, Output displacement of the actuator array at different frequencies. Driving frequency: 5 Hz-50 Hz; sampling frequency: 1000 Hz; time interval: 2s.


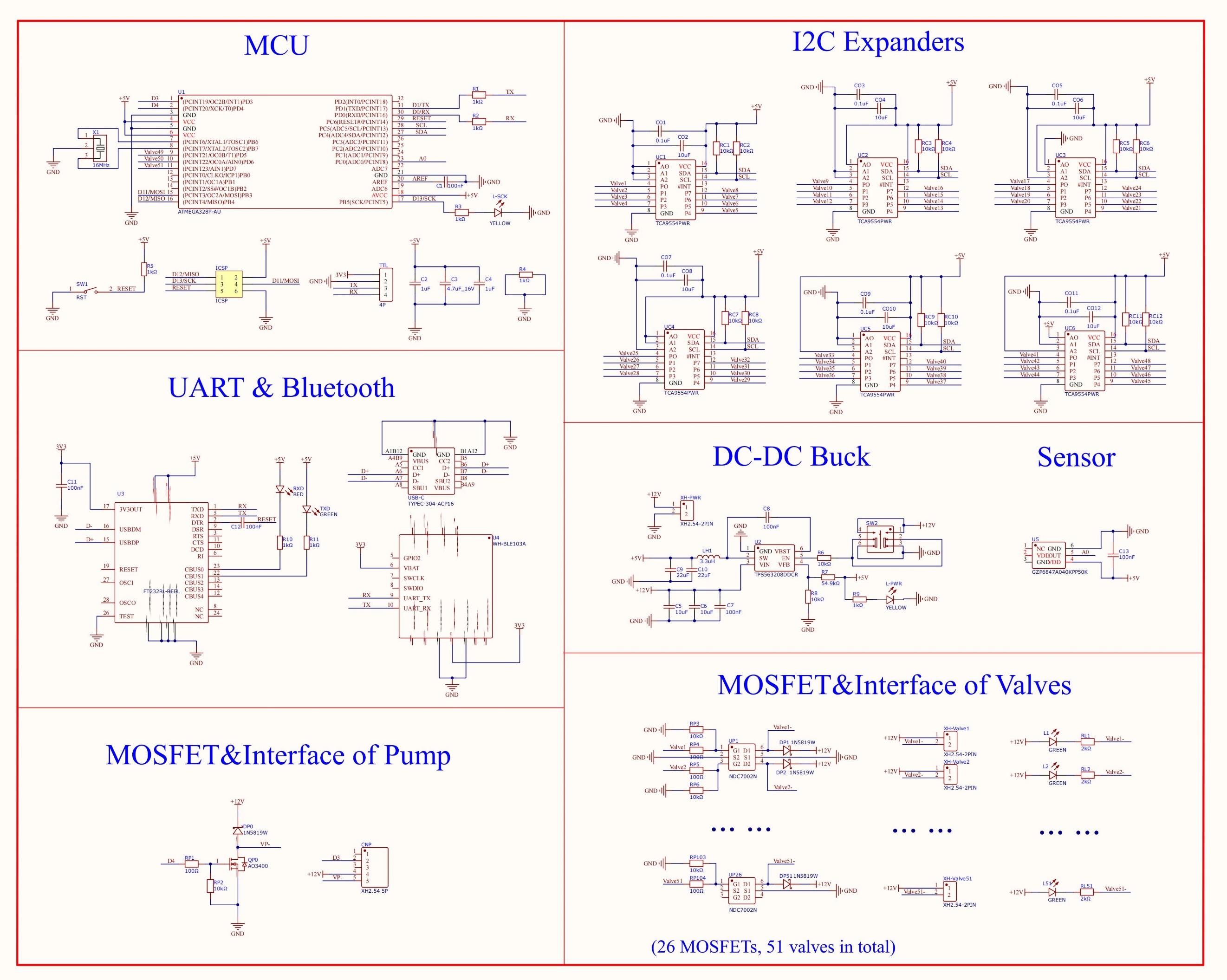


Supplementary Fig. 25 | Schematic design of the independent control system for 7 × 7 actuator array.


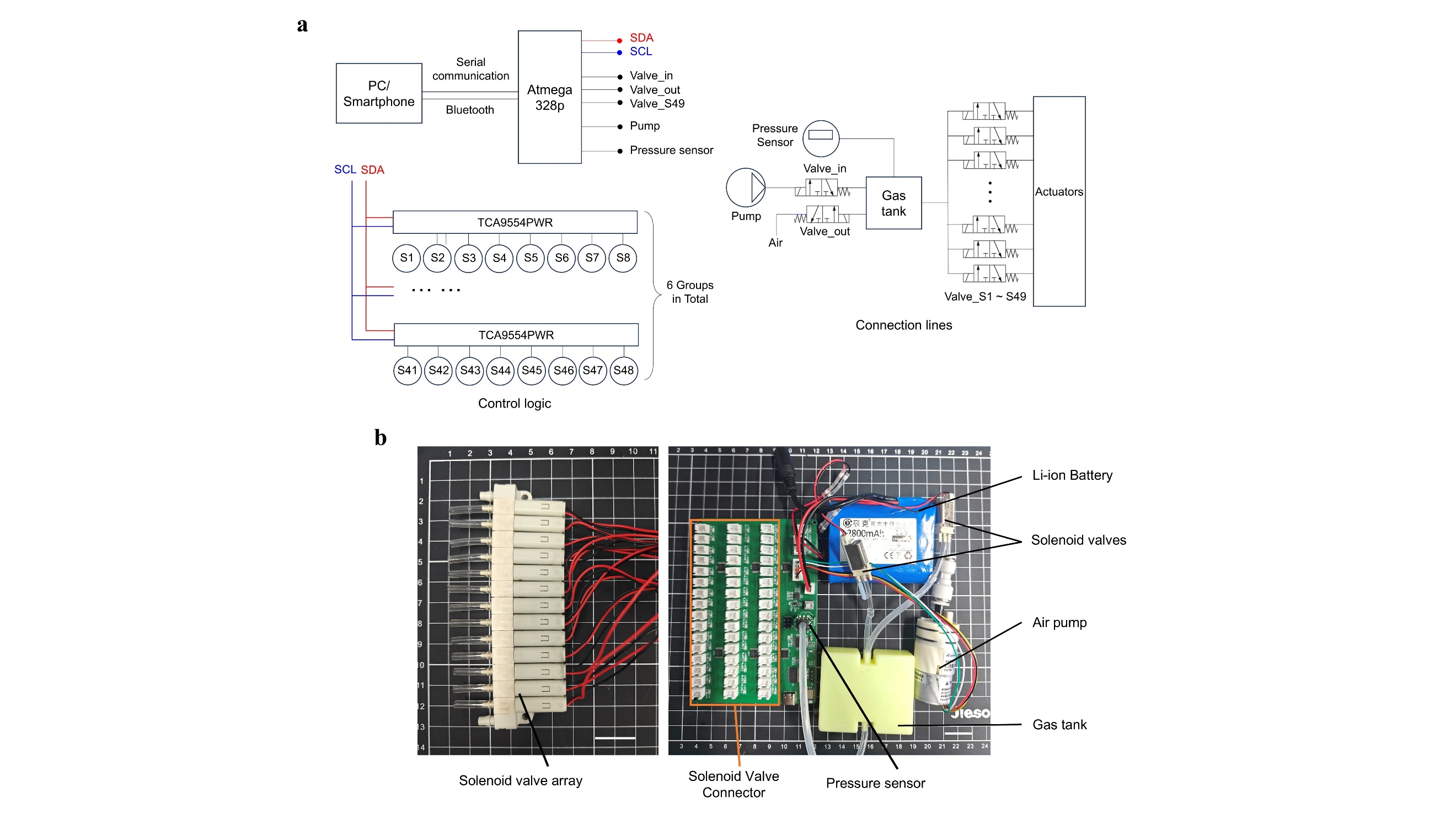


Supplementary Fig. 26 | Control system of microfluidic actuator array. a, The control system processes sensor data and manages components such as pumps and valves through the Atmega 328p microcontroller, ultimately controlling the microfluidic actuator array. Commands are sent from a PC or smartphone to the MCU via a serial port, and the MCU’s control signals regulate the valves and air pumps through I/O interfaces and MOSFETs, adjusting the air pressure and driving the actuators. b, Optical photos of the control system. The names of each part are labeled on the figure. The solenoid valve array on the left is connected to the responsive connector on the right. Scale bar, 3 mm.


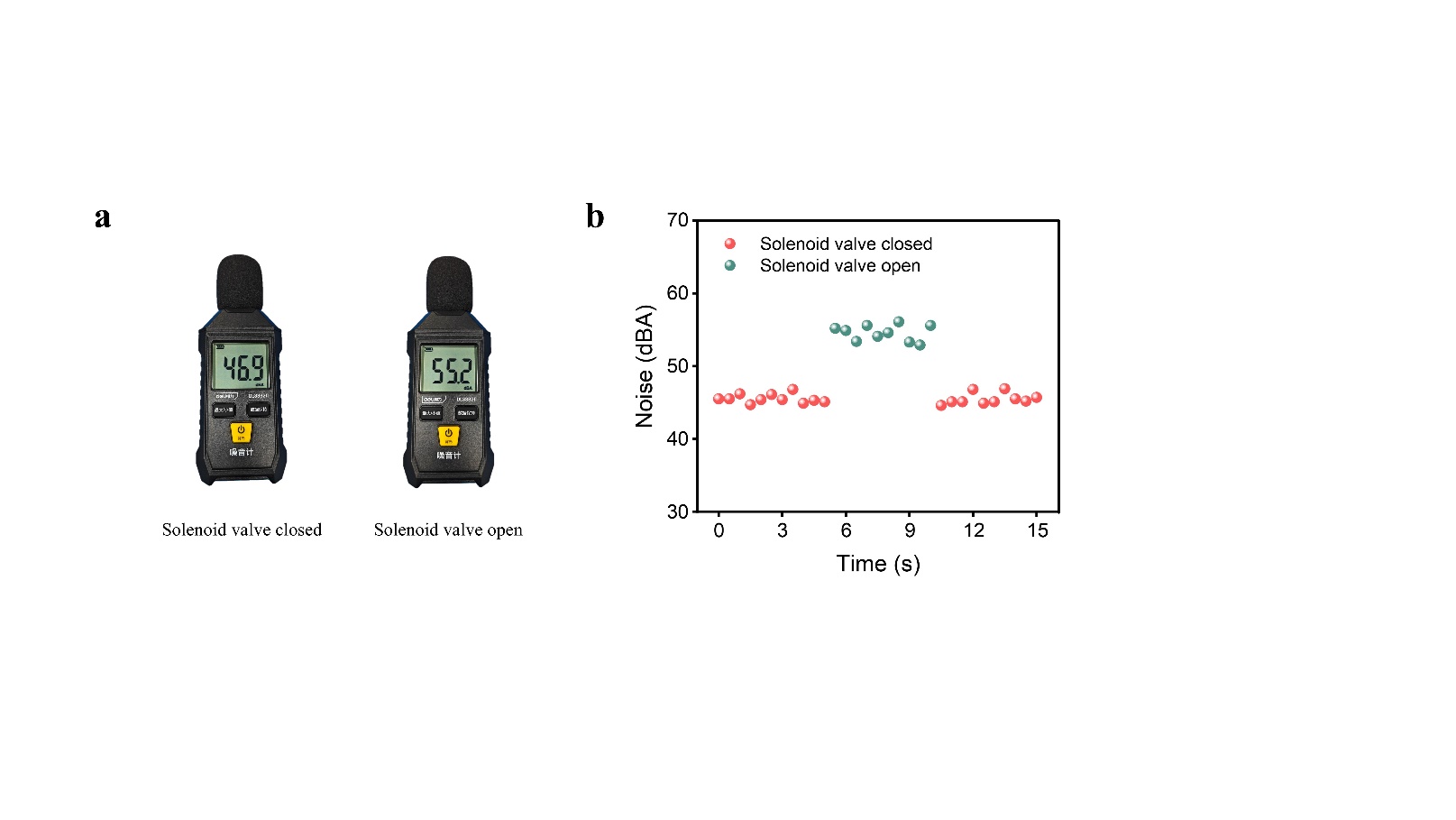


Supplementary Fig. 27 | Evaluation of audible noise generated by solenoid valve activation. a, Photographs of the sound level meter (deli digital sound level meter) during solenoid valve closed and open states, respectively. b, Time-resolved noise measurements showing the sound pressure levels (dBA) over a 15-second interval. The first 5 seconds and last 5 seconds represent ambient noise levels with solenoid valves closed, while the middle 5 seconds correspond to the solenoid valves being open. The sound level meter sampled at a rate of 2 Hz, yielding a total of 30 data points. The average noise level increased from 45.51 ± 0.65 dBA (valves closed) to 54.57 ± 1.04 dBA (valves open), indicating a perceptible but moderate increase in sound during operation.


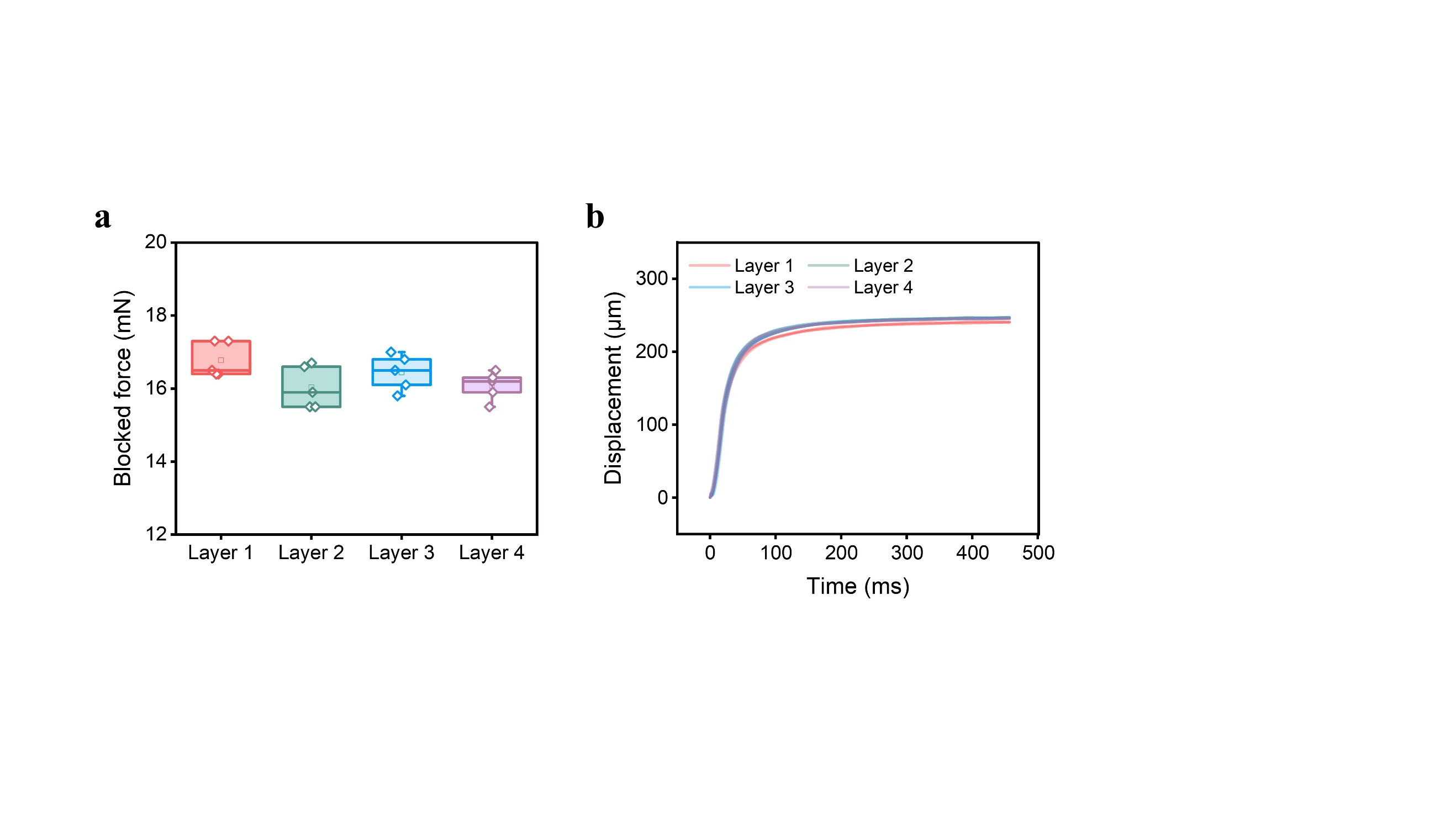


Supplementary Fig. 28 | Consistency of actuator performance across structural layers. a, Measured blocked force of actuators from four structural layers under an input pressure of 70 kPa. Square, mean; centre line, median; box limits, upper and lower quartiles; whiskers, 1.5× interquartile range; points, outliers; n = 5 independent samples. b, Displacement-time curves of actuators from the same layers, recorded under identical loading conditions. Points, mean; error bars, s.d.; n = 3 independent samples. The results show minimal variation in blocking force and highly consistent dynamic response across devices.


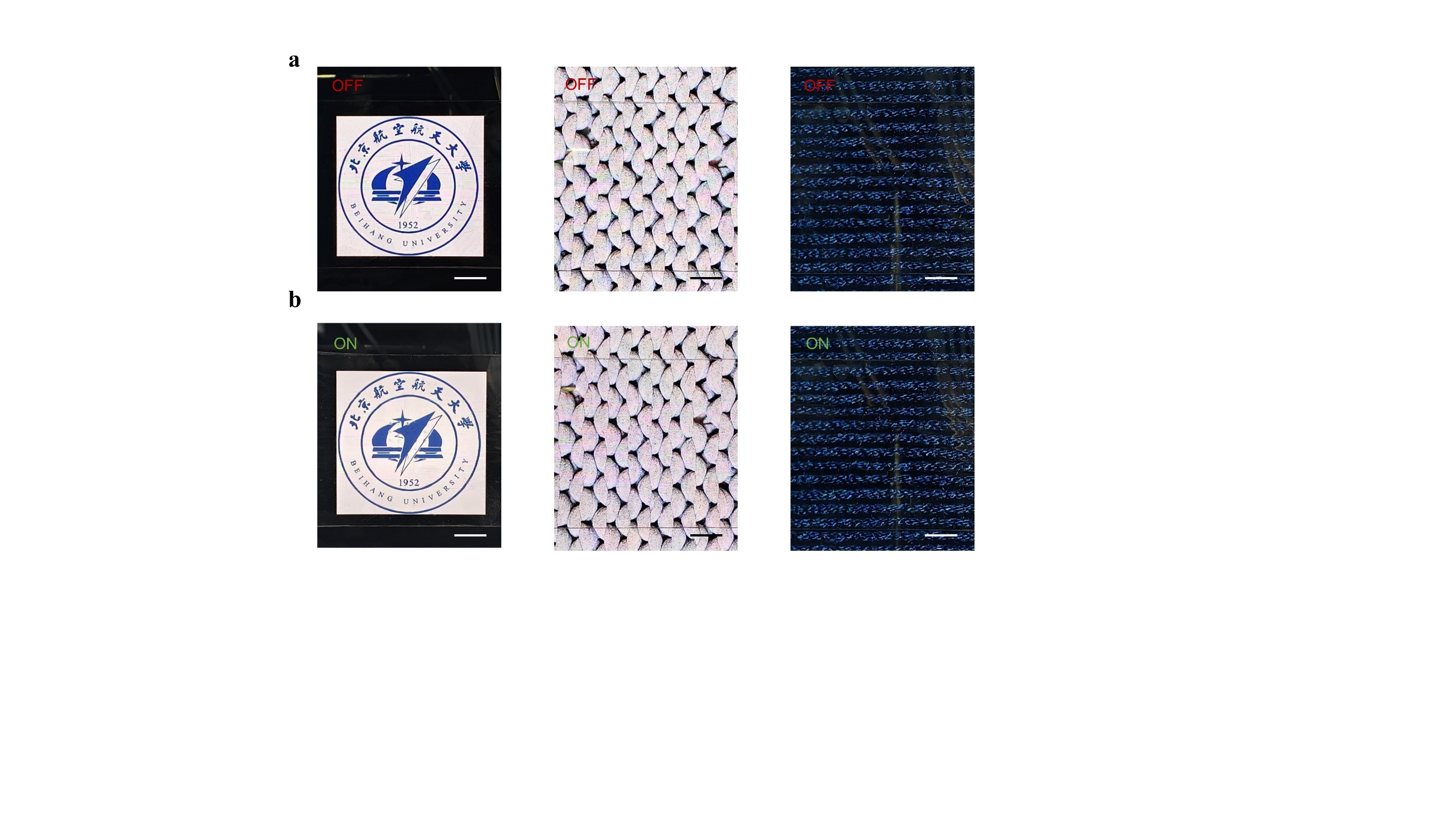


Supplementary Fig. 29 | Optical images of the haptic interface before and after actuation. a, Optical image of the touchscreen graphics with the attached haptic interface (inactive). b, Optical image of the touchscreen graphics with the attached haptic interface (actuated). Scale bar, 5 mm.


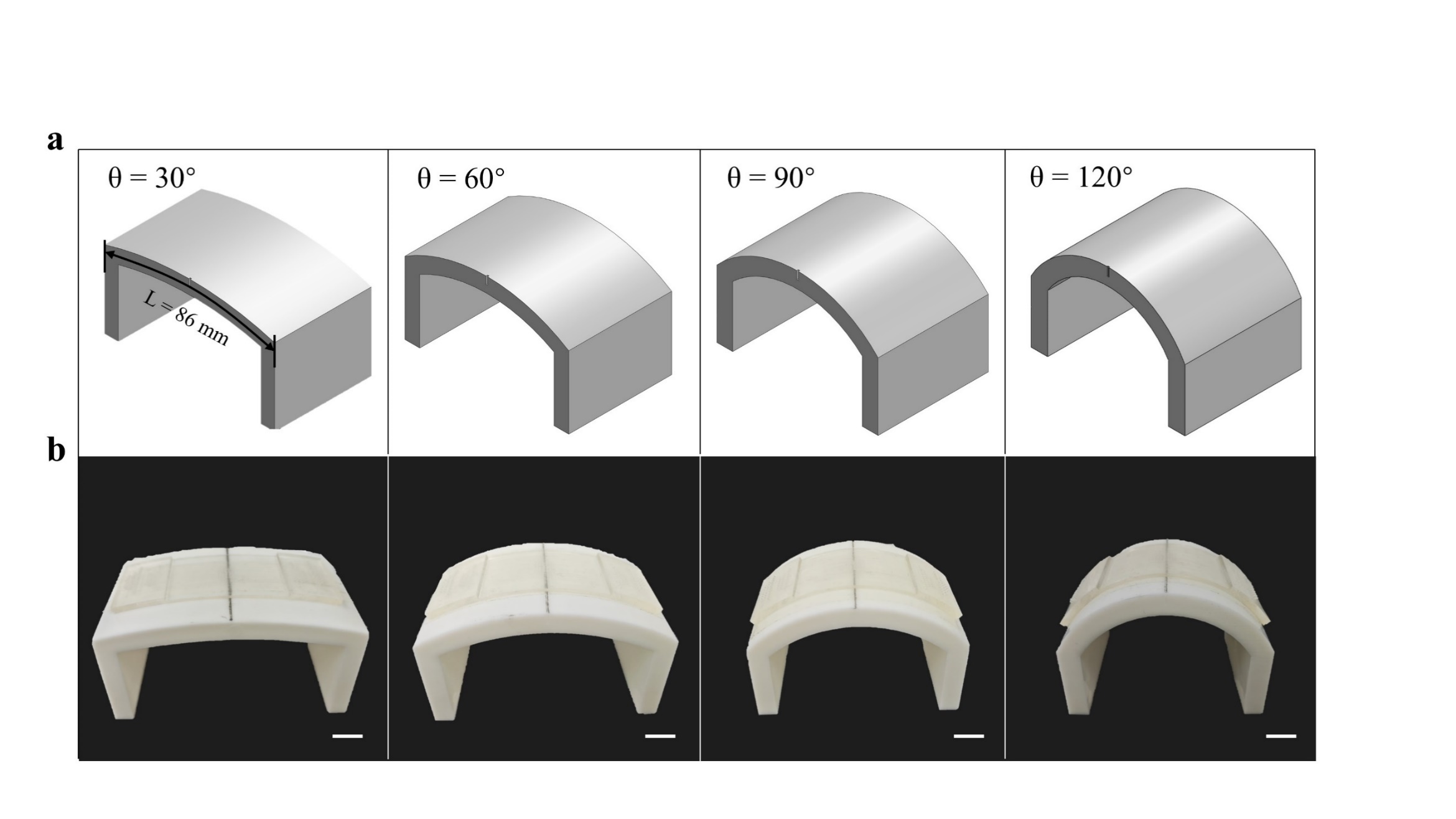


Supplementary Fig. 30 | Conformal properties of the haptic interface on different curved surfaces. a, 3D models of the experimental setups with 4 different curvatures, corresponding to the bending angles of 30°, 60°, 90° and 120° respectively. The arc length of these experimental samples is 86 mm. b, Optical images of flexible haptic interface conformally attached to surfaces of different curvatures. Scale bar, 10 mm.


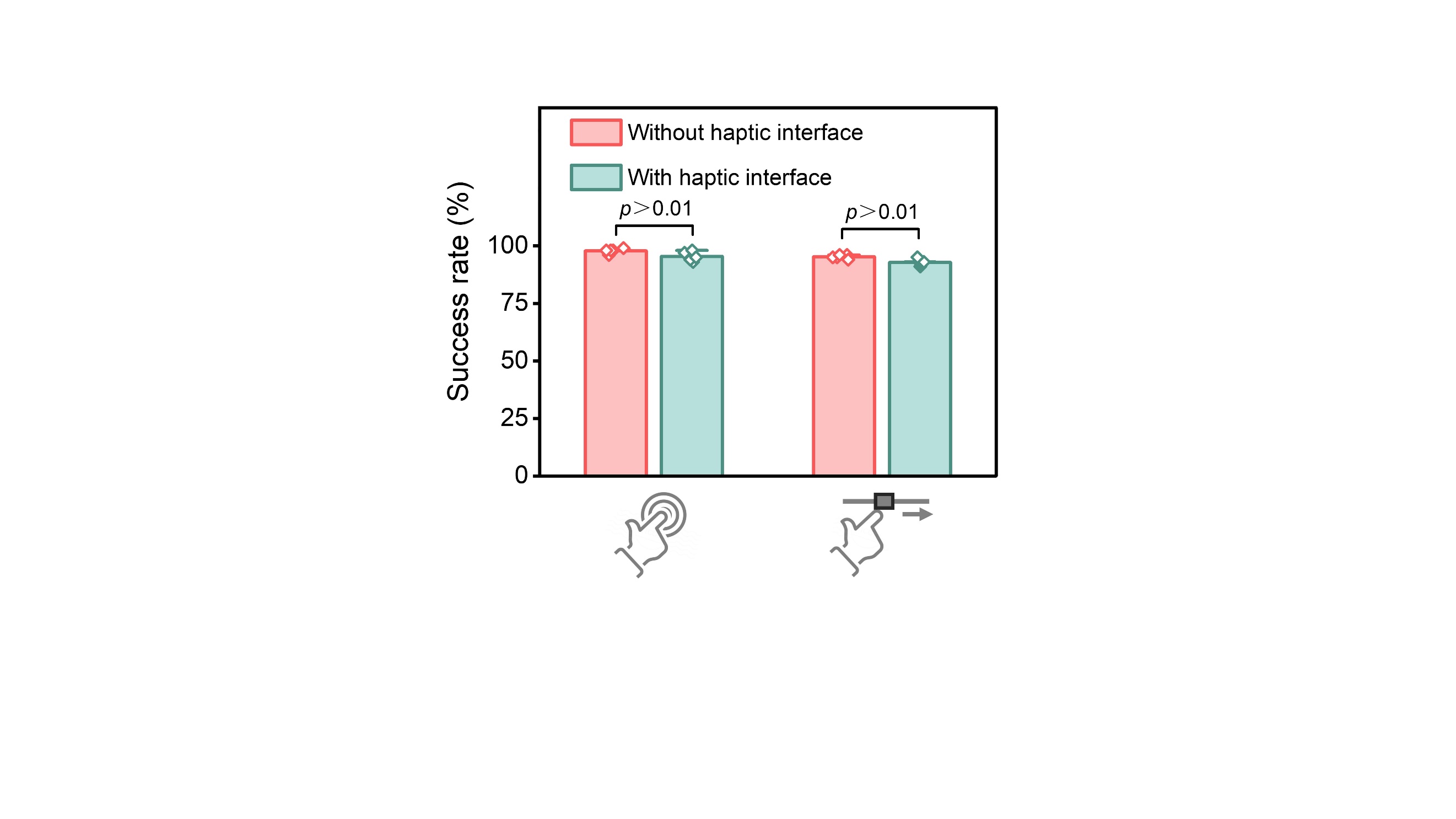


Supplementary Fig. 31 | Comparison of the operation success rates of a touchscreen with and without haptic interfaces. Bar height, mean; error bars, s.d.; *n* = 5 independent samples. Statistical significance was assessed using a paired sample t-test.


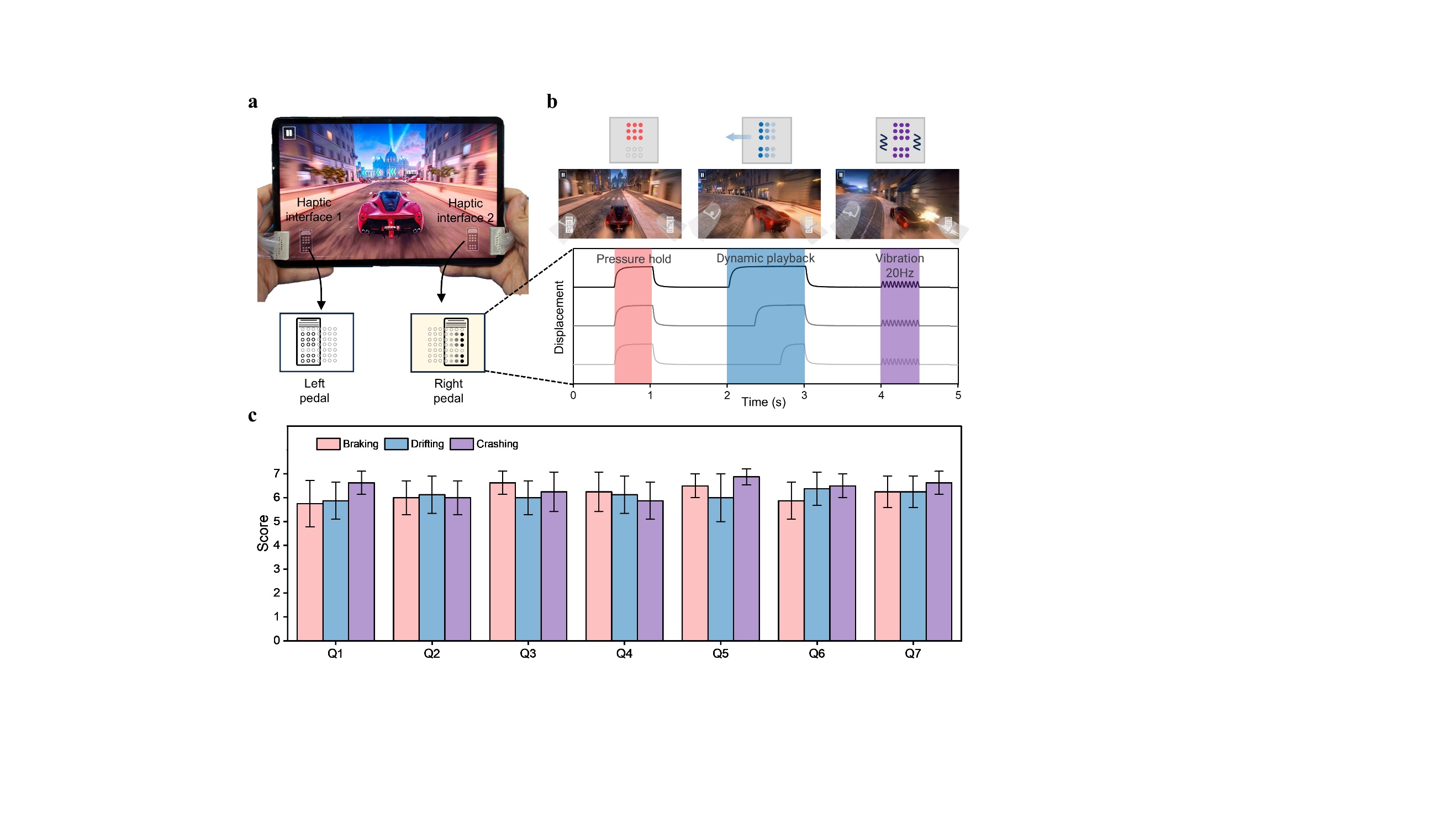


Supplementary Fig. 32 | High-resolution haptic feedback for immersive touchscreen interaction in a driving simulation. a, A tablet computer integrated with a pair of tactile interfaces. A pair of microfluidic haptic interfaces is attached to the left and right sides of the touchscreen, simulating pedals for interactive driving control. The system enables localized tactile cues corresponding to in-game events. b, Actuator displacement over time. The actuators are categorized into three groups, each represented by a different depth of color. Three distinct tactile feedback modes are demonstrated: pressure hold (braking), dynamic playback (drifting), and 20 Hz vibration (crashing). Each mode corresponds to different in-game driving maneuvers, providing spatially and temporally resolved tactile stimulation to enhance user immersion. c, User evaluation of haptic feedback effectiveness. Participants rated the perceived realism and effectiveness of haptic cues across three conditions (braking, drifting, and crashing) for different in-game scenarios (Q1–Q7) (table S4). The results indicate consistent performance across conditions, validating the system’s capability for real-time, high-resolution tactile rendering in interactive applications. Bar height, mean; error bars, s.d.; n = 8 independent samples.


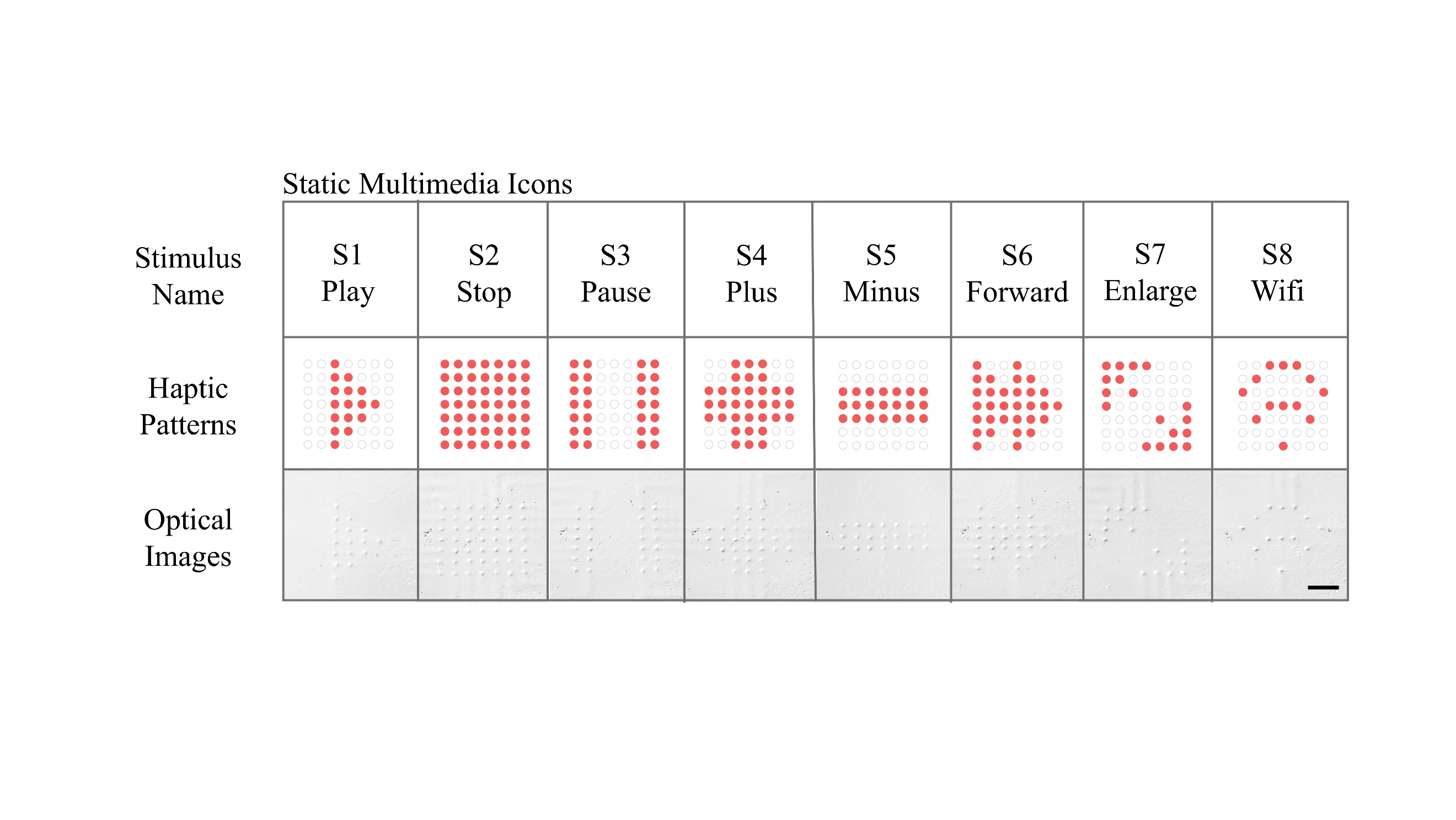


Supplementary Fig. 33 | Schematic diagrams and optical images of static multimedia icons in user study. The surface of haptic device is coated gray to highlight the transparent domes (pixels). Scale bar, 3 mm.


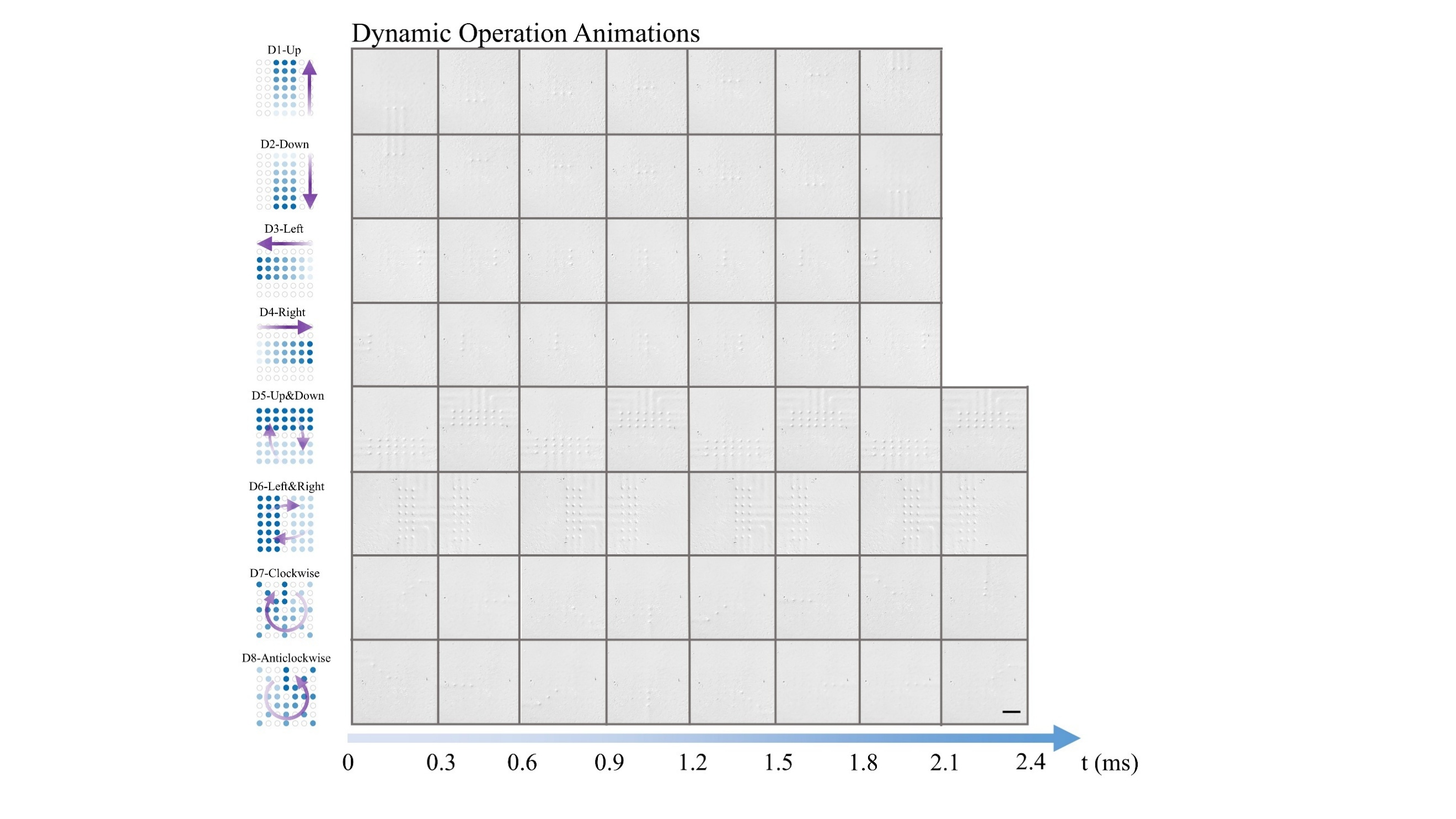


Supplementary Fig. 34 | Schematic diagrams and optical images of dynamic operation animations in user study. The surface of the haptic device is coated gray to highlight the transparent domes (pixels). Scale bar, 3 mm.


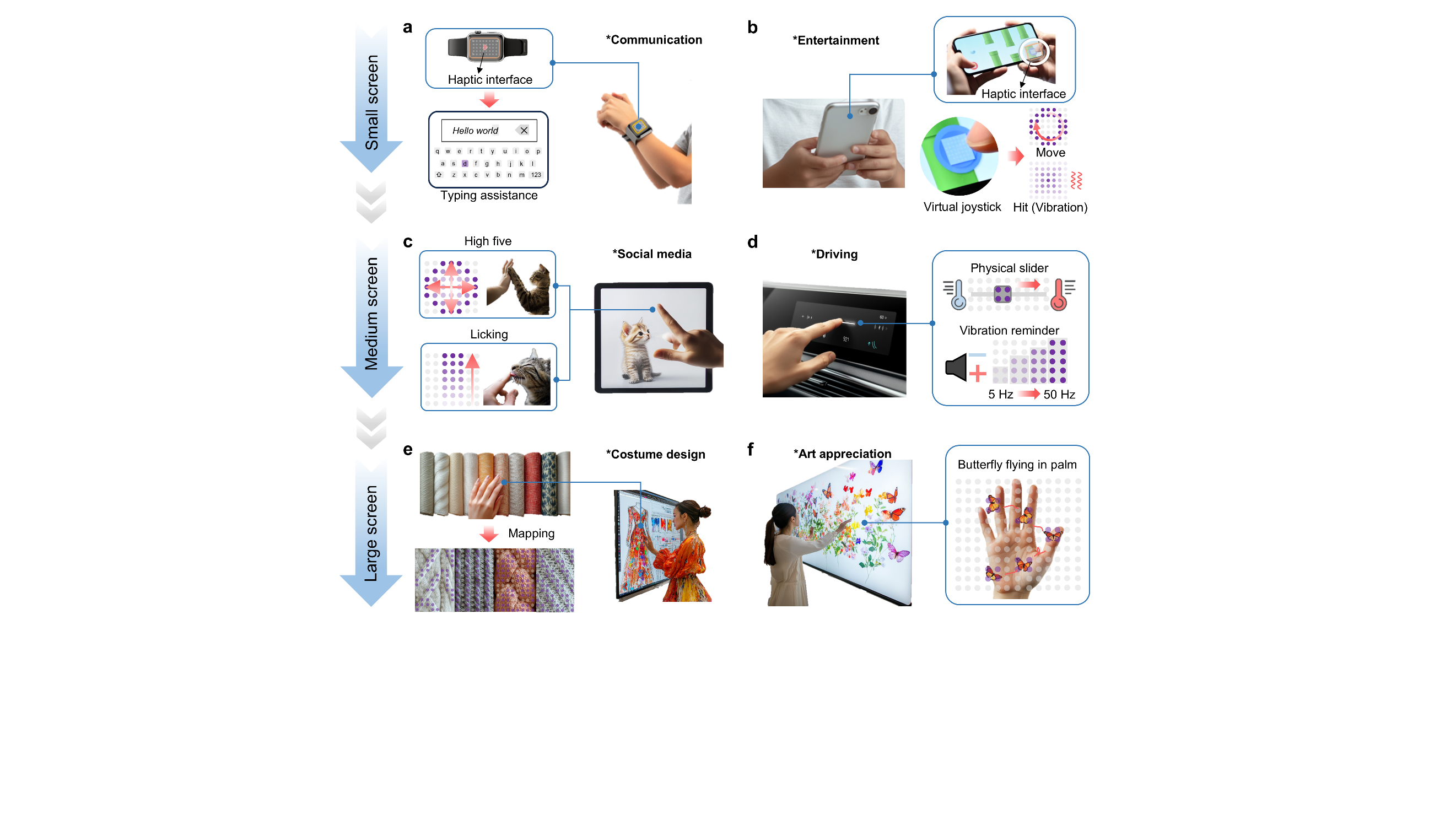


Supplementary Fig. 35 | Future applications of the morphable haptic interface for touchscreens. This interface demonstrates its versatility by integrating with various touchscreen devices, facilitating high-resolution, programmable haptic feedback across multiple interaction scenarios: a, A smartwatch featuring a transparent physical keyboard, enhancing typing accuracy by defining key boundaries through localized tactile feedback. b, A smartphone with a virtual joystick, delivering haptic cues for directional guidance and impact sensations. The color gradient represents vibration intensity. c, A tablet where users interact with a virtual cat, experiencing haptic animations corresponding to the cat’s movements. The color depth signifies actuator timing. d, A central control screen facilitating sliding interactions without visual cues, where a physical slider adjusts temperature and a vibration band modulates volume. Vibration intensity is depicted by a color gradient. e, A PC-based fashion design software, allowing designers to assess the tactile sensations of virtual fabrics, refining the creative process. f, A display screen that enhances interactive immersion by enabling users to feel butterflies fluttering among flowers, transforming visual engagement into a multi-sensory experience.

Supplementary Table 1. Comparison of our work with the morphable haptic interfaces reported recently.

| Actuation Principle | Spatial Resolution  (mm) | Function | Programmability | Transparency | Structure Invisibility | Ref |
| --- | --- | --- | --- | --- | --- | --- |
| Hydraulic | ~15 | Button | N | Y | Y | 1 |
| Electromagnetic | 8 | Button | Y | N | N | 2 |
| Electroosmotic | 10 | Button | Y | N | N | 3 |
| Pneumatic | ＞20 | Button | N | N | N | 4 |
|  | 13-14 | Button | Y | Y | N | 5 |
| Hydrogel | 4 | Button | Y | N | N | 6 |
| Electrohydraulic | ＞10 | Button | Y | Y | N | 7 |
| Hydraulic | 1.5 | Pattern, Texture,  Slider,  Virtual joystick | Y | Y | Y | This work |

Supplementary Table 2. Refractive index of common transparent liquids.

| Liquid | Refractive Index | Toxic | Conductive | Colorless | Chemical Stability |
| --- | --- | --- | --- | --- | --- |
| Water (H₂O) | 1.333 | No | No | Yes | Stable |
| Glycerol (C₃H₈O₃) | 1.474 | No | No | Yes | Stable |
| Dimethyl silicone | 1.400-1.410 | No | No | Yes | Stable |
| NaCl solution (0.9%) | 1.340 | No | Yes | Yes | Stable |
| Sucrose solution (10%) | 1.344 | No | No | Yes | Stable |
| Ethanol (C₂H₅OH) | 1.361 | Yes | No | Yes | Stable |
| Acetone (C₃H₆O) | 1.359 | Yes | No | Yes | Stable |
| Benzene (C₆H₆) | 1.501 | Yes | No | Yes | Unstable (to oxidize) |
| Toluene (C₇H₈) | 1.496 | Yes | No | Yes | Stable |
| Methanol (CH₃OH) | 1.328 | Yes | No | Yes | Stable |
| Dimethyl sulfoxide (DMSO) | 1.479 | Yes | No | Yes | Stable |

Supplementary Table 3. Comparison of our work with the fluid-driven tactile displays reported recently.

| Structure Type | Spatial Resolution  (mm) | Diameter of actuator  (mm) | Effective Surface Area(mm^2^) | Vertical Displacement (mm) | Ref |
| --- | --- | --- | --- | --- | --- |
| Chambers & Channels on the single layer | 20 | 8 | 50.27 | ~16.3 | 8 |
|  | 13-14 | 7.5 | 44.18 | 3.0 | 5 |
|  | 4 | 3 | 7.07 | 0.50 | 9 |
| Chambers & Channels on different layers | 2.5 | 1.5 | 1.77 | 0.56 | 10 |
|  | 2.5 | 1.5 | 1.77 | 0.50 | 11 |
|  | ≥2.5 |  | / | ≤1.5 | 12 |
| Channel-free structure | 4 | 3 | 7.07 | 0.50 | 13 |
|  | 22 | 4 | 12.57 | 0.87 | 14 |
| Inverted pyramid chamber array with layered channel Connection | 1.5 | 1 | 0.79 | ＞0.27 | This work |

Supplementary Table 4. A questionnaire to evaluate the various effects of tactile feedback.

| Questions | Introduction |
| --- | --- |
| 1 | I like having the haptic feedback as part of my experience |
| 2 | I felt adequate variations in the haptic feedback |
| 3 | The haptic feedback reflects varying inputs and events |
| 4 | I felt engaged with the system due to the haptic feedback |
| 5 | The haptic feedback was realistic/convincing |
| 6 | The haptic feedback felt appropriate where I felt it |
| 7 | The visual and tactile fusion experience is not exhausting or tiring |

Supplementary Video 1. Filling and actuation process of the transparent microfluidic actuator.

This video highlights the gradual invisibility of the internal structure of the transparent microfluidic actuator during the filling process. Once the outlet is sealed, the actuator surface exhibits varying degrees of out-of-plane deformation under different input pressures (0-40kPa).

Supplementary Video 2. Refractive index matching for optical invisibility.

This video shows the refractive index matching technique used for achieving optical invisibility, illustrating how the material blends into its surroundings.

Supplementary Video 3. Deformation of the flexible haptic interface.

This video captures the flexible haptic interface undergoing twisting, stretching, and bending under different forces, demonstrating its adaptability and flexibility. These characteristics enable it to conform easily to touchscreens of various formats.

Supplementary Video 4. Simultaneous display of fabric texture images and surface topographies on the touchscreen.

This video illustrates the simultaneous display of fabric texture images and corresponding surface topographies on the touchscreen, providing a multisensory experience with both visual and tactile feedback.

Supplementary Video 5. Demonstration of reconfigurable static tactile icons.

This video demonstrates how a haptic interface can reconfigure tactile icons based on the user’s interaction tasks, enabling tactile-based interaction in specific scenarios.

Supplementary Video 6. Demonstration of programmable dynamic tactile patterns.

This video showcases the functionality of programmable dynamic tactile patterns, which change in real time based on different programming inputs. The patterns provide tactile feedback, and users can receive tactile instructions by maintaining contact with the interface.

1. Barde, C. R. & Purkar, K. T. A dynamic touch screen interface like Tactus for mobile and automotive devices. *Int. J. Emerg. Technol. Adv. Eng.* **3**, 351–356 (2013).
2. Iwata, H., Yano, H., Nakaizumi, F. & Kawamura, R. Project FEELEX: adding haptic surface to graphics. *In Proceedings of the 28th Annual Conference on Computer Graphics and Interactive Techniques (SIGGRAPH 2001)* (ACM, 2001), 469–476.
3. Shultz, C. & Harrison, C. Flat panel haptics: embedded electroosmotic pumps for scalable shape displays. *Proc. 2023 CHI Conf. Hum. Factors Comput. Syst. (CHI 2023)* **745**, 1–16 (2023).
4. Harrison, C. & Hudson, S. E. Providing dynamically changeable physical buttons on a visual display. *Proc. 2009 CHI Conf. Hum. Factors Comput. Syst. (CHI 2009)* 299–308 (2009).
5. Russomanno, A., Xu, Z., O’Modhrain, S. & Gillespie, B. A pneu shape display: physical buttons with programmable touch response. *2017 IEEE World Haptics Conf. (WHC 2017)* 641–646 (2017).
6. Miruchna, V. *et al*. GelTouch: localized tactile feedback through thin, programmable gel. *UIST’15: Proceedings of the 28th Annual ACM Symposium on User Interface Software & Technology* 3–10 (2015).
7. Firouzeh, A., Mizutani, A., Groten, J., Zirkl, M. & Shea, H. PopTouch: a submillimeter thick dynamically reconfigured haptic interface with pressable buttons. *Adv. Mater.* **36**, 2307636 (2024).
8. Robinson, S. S. *et al.* Integrated soft sensors and elastomeric actuators for tactile machines with kinesthetic sense. *Extrem Mech Lett* **5**, 47–53 (2015).
9. Heisser, R. H. *et al.* Valveless microliter combustion for densely packed arrays of powerful soft actuators. *Proc Natl Acad Sci USA* **118**, e2106553118 (2021).
10. Wu, X., Kim, S. H., Zhu, H. & Allen, M. G. A refreshable Braille cell based on pneumatic microbubble actuators. *J Microelectromech Syst* **21**, 908–916 (2012).
11. Russomanno, A., Gillespie, R. B., O’Modhrain, S. & Barber, J. A tactile display using pneumatic membrane actuators. *Proc Eurohaptics*, 445–447 (IEEE, 2014).
12. HaptX. Haptic gloves for virtual reality and robotics. <https://haptx.com/technology/>
13. Velazquez, R., Pissaloux, E. E., Hafez, M. & Szewczyk, J. Tactile rendering with shape-memory-alloy pin-matrix. *IEEE Trans Instrum Meas* **57**, 1051–1057 (2008).
14. Hwang, I., Mun, S. & Youn, J. H. Height-renderable morphable tactile display enabled by programmable modulation of local stiffness in photothermally active polymer. *Nat Commun* **15**, 2554 (2024).
